# EEG emotion-recognition datasets for neuroarchitecture: an appraisal of openness, AI-readiness and ontology maturity

**DOI:** 10.3389/fninf.2026.1971441

**Published:** 2026-10-20

**Authors:** Seung Yeul Ji, Mi Kyoung Kim, Han Jong Jun

**Affiliations:** 1 LINK4EEG Lab, School of Architecture, Hanyang University, Seoul, Republic of Korea; 2 School of Built Environment, Faculty of Arts, Design & Architecture, The University of New South Wales, Sydney, NSW, Australia; 3 ADCC, School of Architecture, Hanyang University, Seoul, Republic of Korea

**Keywords:** affective computing, EEG, emotion ontology, emotion recognition, FAIR principles, neuroarchitecture, physiological computing, PRISMA 2020

## Abstract

**Introduction:**

Neuroarchitecture studies how designed space shapes emotion, often with electroencephalography (EEG), and computational models that decode emotion from EEG must be trained on labelled recordings. Yet none of the widely shared EEG emotion datasets was recorded while people moved through designed spaces, and the few spatial recordings we found, all released since 2023, are single studies, each cited at most once. Researchers therefore borrow general emotion datasets, recorded from seated participants watching films, listening to music, playing games or conversing, and must decide whether each can be found, obtained and reused for transfer learning. This review contributes a reusable method for that decision and applies it.

**Methods:**

Following PRISMA 2020 with the dataset as the unit of analysis, two search passes identified 27 publicly obtainable datasets (22 in the first, 5 in the second), none designed for spatial research. A published codebook of 52 criteria defines three instruments: openness, judged against the FAIR principles with access class recorded separately; AI-readiness, the equally weighted mean of three sub-scores (label information, fit to standard tasks and alignment with other data), which describes usefulness as a transfer source; and ontology maturity, on five levels, which describes how far emotion labels can be mapped onto other vocabularies. Every identifier and access route was checked.

**Results:**

Openness was weakest in Interoperability (2.044 against a FAIR composite of 2.857), and only 8 datasets were fully open. A dataset-level identifier was missing for 13 of 27 datasets (48.1%), and three widely used datasets could not be reached through their published routes at a re-check on 11 September 2026. AI-readiness averaged 2.684 on a 1.0–4.0 scale (median 2.60, range 2.10–3.33), and ontology levels concentrated at Level 3 (16 of 27), with none at Level 4 or 5. Openness and readiness were nearly unrelated (*ρ* = 0.08), whereas ontology level tracked readiness (*ρ* = 0.68), partly because both read the label structure.

**Discussion:**

Open access alone is therefore a poor guide to reuse. The method, codebook and scripts are published so that researchers in fields without dedicated data can test the datasets they plan to reuse.

## 1 Introduction

Architecture shapes how people feel. A daylit atrium, a narrow corridor, a legible route or a biophilic interior can each evoke comfort or stress and an urge to approach or to avoid. Neuroarchitecture studies these responses with neurophysiological measures. Electroencephalography (EEG) suits this work because it is portable and temporally precise. Mobile brain/body imaging (MoBI) records brain and body dynamics together in people who move and act in their environment (Gramann et al., 2011). In one neuroarchitecture study, EEG recorded while participants walked through virtual interiors showed effects of interior form (Banaei et al., 2017).

Computational models that decode emotion from EEG must first be trained on labelled recordings, and they work best on data like those they were trained on. A neuroarchitecture laboratory therefore needs EEG recorded while people experience designed spaces, labelled with what they felt. Such data are scarce. No dataset in the shared benchmark tier, the corpora on which the decoding literature trains and compares its models, was recorded while people moved through designed spaces. Several virtual-reality (VR) emotion datasets record no scalp EEG (VREED, Tabbaa et al., 2021; MVRS, Mousavi and Ilanloo, 2025). The spatially situated EEG releases found by our coverage check (Section 4.4) are recent single studies (Ramirez-Lechuga et al., 2023; Tomar et al., 2025; Dou, 2026; Ming and Zhang, 2026; Wu et al., 2026). By 12 September 2026 each had been cited at most once in OpenAlex, and none supplies benchmark splits, a standard data schema or code that loads the data for model training.

What exists in quantity is emotion EEG. These recordings carry established affect measures: self-ratings of valence (how pleasant a state feels) and arousal (how activated it is), or discrete emotion categories. They were made while seated participants watched films, listened to music, played games, imagined emotional scenarios or held conversations.

Russell’s circumplex model places feelings in a space defined by two dimensions, valence and arousal (Russell, 1980). This is a space of feelings, not a physical space. The link to places comes from Russell and Pratt (1980), who described the affective quality that people attribute to places on the same pleasantness and arousal dimensions. Several of the spatial EEG releases collect ratings on these dimensions. The audiovisual VR and SPACE datasets use the Self-Assessment Manikin, a pictorial rating scale, for valence or pleasure, arousal and dominance, and HERO records valence and arousal beside comfort. What might carry over from emotion EEG to a building study is therefore the learned relation between EEG and such ratings, or shared emotion categories. The stimuli themselves do not carry over.

For neuroarchitecture, the realistic use of these corpora is transfer learning, in which a model is pre-trained on a large source dataset and then adapted with a smaller dataset from the target setting. Even between two seated corpora, a model loses much of its accuracy when it is moved from one to the other (Cimtay and Ekmekcioglu, 2020). Adaptation recovers only part of that loss (Lan et al., 2019). Spatial experience adds two further differences: people move, and they choose where to look (Section 4.3). Whether the relation between EEG and affect survives these differences has not been tested. Seated emotion corpora are therefore candidate sources for pre-training, not stand-ins for spatial data. The practical question is which of them are fit for that role.

The EEG emotion-recognition literature is organised around decoding models. Reviews and benchmarks repeatedly report which classifier or deep network achieves the highest accuracy on DEAP or the SEED family (Koelstra et al., 2012; Zheng and Lu, 2015), treating the underlying datasets as fixed, interchangeable givens. Reported accuracy is not a property of a dataset, because it depends on the method, the data split and the preprocessing. A ranking by accuracy therefore cannot tell a laboratory which corpus it can legally and technically reuse.

Three properties of the data decide that question, and they form the three axes of this appraisal. The first is openness: whether a dataset has a persistent identifier, a licence, a standard format such as BIDS-EEG (Pernet et al., 2019) and a datasheet (Gebru et al., 2021). Openness is judged against the FAIR principles of findability, accessibility, interoperability and reusability (Wilkinson et al., 2016). The second is AI-readiness, treated here as a property of the data: how informative its labels are, which standard tasks it supports, and how well its labels and channels can be aligned with another dataset. The third is ontological maturity, meaning how formally the emotion labels are structured. Its five levels run from a flat label, through a basic-emotion taxonomy of named categories and a relational or dimensional affect model (for example valence-arousal-dominance), to a formal emotion ontology and an emotion knowledge graph.

Ontological maturity matters because a label learned from one corpus helps a building study only if it can be translated into what that study measures. Spatial studies measure valence and arousal, but also comfort, pleasantness and the willingness to approach or avoid (Section 4.3). A flat positive or negative label maps onto one bipolar judgement only. Named categories can be matched across corpora by name. A dimensional coordinate can be set beside any rating collected on the same axes. Only a formal ontology can declare, and let software check, how a source label relates to a concept such as comfort or approach. The levels are therefore ordered by the mappings they allow. To the best of the authors’ knowledge, no prior synthesis has appraised EEG emotion datasets jointly on all three axes, or framed that appraisal for neuroarchitecture reuse.

The review appraises the 27 persistently documented datasets found by two search passes, 22 in the first and 5 in the second (Sections 2.1 and 2.2). Persistently documented means that a dataset can be reached through a citable descriptor paper or a repository accession. The review asks three research questions of each dataset:

RQ1 (Openness): How open, findable and reusable are the datasets, and how does openness vary with access class and data format?

RQ2 (AI-readiness): How ready are the datasets to serve as sources for transfer learning, judged by annotation specificity, task alignment and ontology maturity?

RQ3 (Ontology maturity): How far is emotion knowledge structured in the datasets, and where could it be upgraded towards formal ontologies and knowledge graphs?

The appraisal makes two contributions. The first is a vetted corpus: a per-dataset appraisal that shows a researcher which datasets can be found, obtained and reused as transfer sources, and where a spatial study could start (Section 4.3). The second is a reusable appraisal method (Section 4.2). It combines PICOD scoping (the PICO question format extended with a dataset facet), an eligibility rule based on public obtainability and one published codebook of 52 criteria with scripts that recompute every score. It adds an audit of identifiers and access routes that tests whether a dataset can still be found. The method answers a question that every study reusing other people’s data must answer: should this dataset be used, and can it still be obtained? It can be applied in other fields that also lack dedicated datasets.

## 2 Methods

### 2.1 Design and scope

This review follows the PRISMA 2020 reporting framework (Page et al., 2021), adapted so that the unit of analysis is the dataset, not the paper. The appraisal is bounded to persistently documented corpora (Section 1), because only such corpora can be located, cited and obtained again by another researcher. Coverage was checked against a separately compiled landscape of affective-EEG resources (Section 4.4).

When a corpus carries several persistent identifiers, the scored deposit is the one that its own data descriptor and later reuse cite, normally the original deposit. A later mirror, or a redistribution preprocessed for a particular model input, is a different artefact and is not scored; the identifiers not scored are listed in Supplementary Section S20.

This review was not prospectively registered, and no protocol was deposited in PROSPERO or an equivalent registry.

PICO scoping was built for clinical reviews and does not cover the constraints of a dataset. This review therefore extends PICO with an explicit D (Dataset constraints) facet, yielding a five-element PICOD scoping framework (Figure 1). The D facet is the entry gate: scalp EEG with affect labels, persistent documentation and an access route open to researchers in any country (Section 2.3). The outcome is dataset readiness, and model accuracy is never used as an outcome because it is not a fixed property of a dataset. A facet-by-facet application record is provided in Supplementary Section S10.

**Figure 1:**
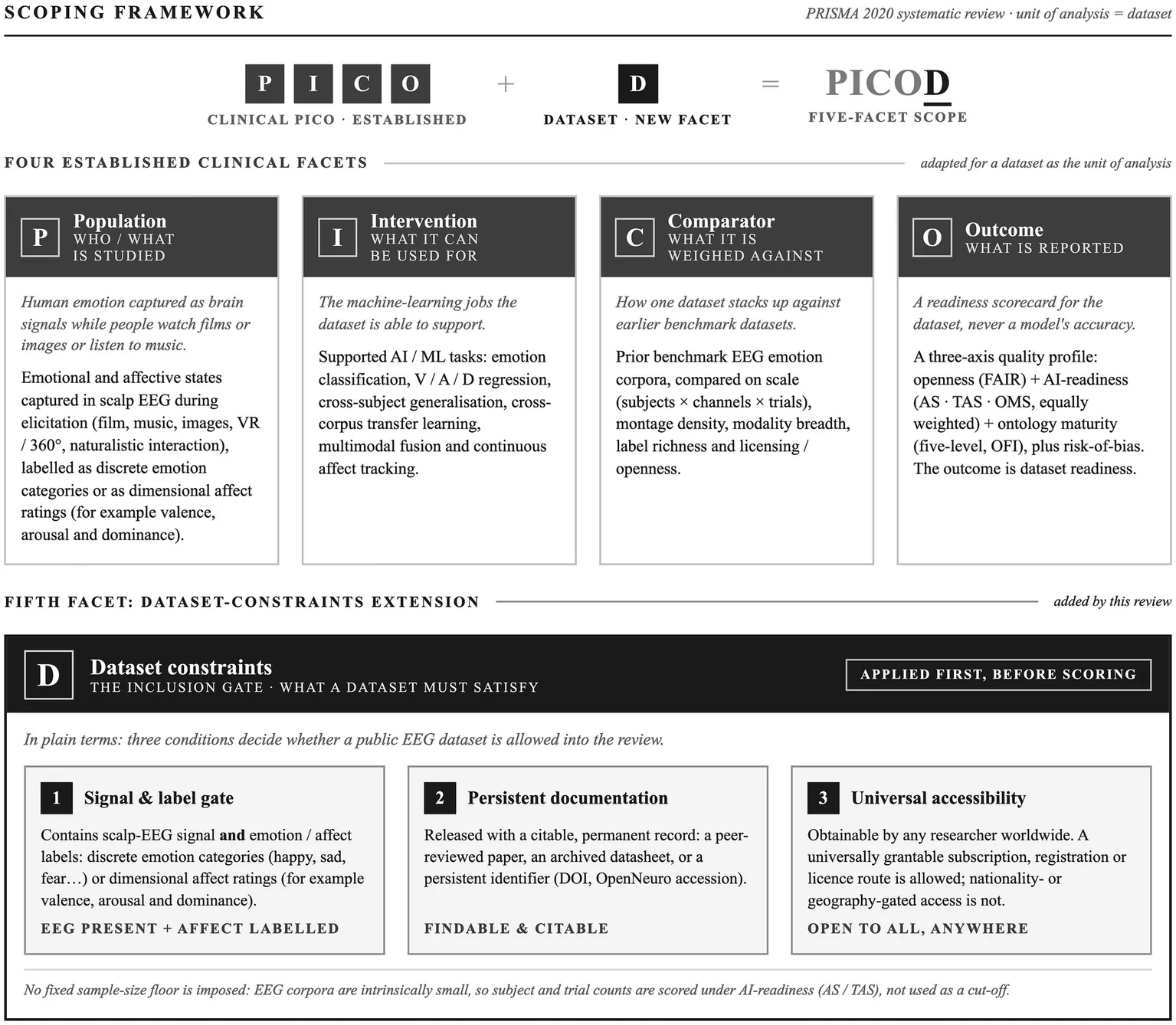
The PICOD scoping framework for dataset-focused systematic reviews, extending the four clinical PICO facets (Population, Intervention, Comparator, Outcome) with a fifth Dataset-constraints (D) facet that encodes the scalp-EEG-and-affect-label gate, persistent-documentation requirement and universal-accessibility requirement.

Figure 2 shows how the three research questions, the corpus and the three appraisal instruments connect. The corpus was assembled in two search passes: 22 datasets in the first and 5 in the second, 27 in all, listed in Table 1.

**Figure 2:**
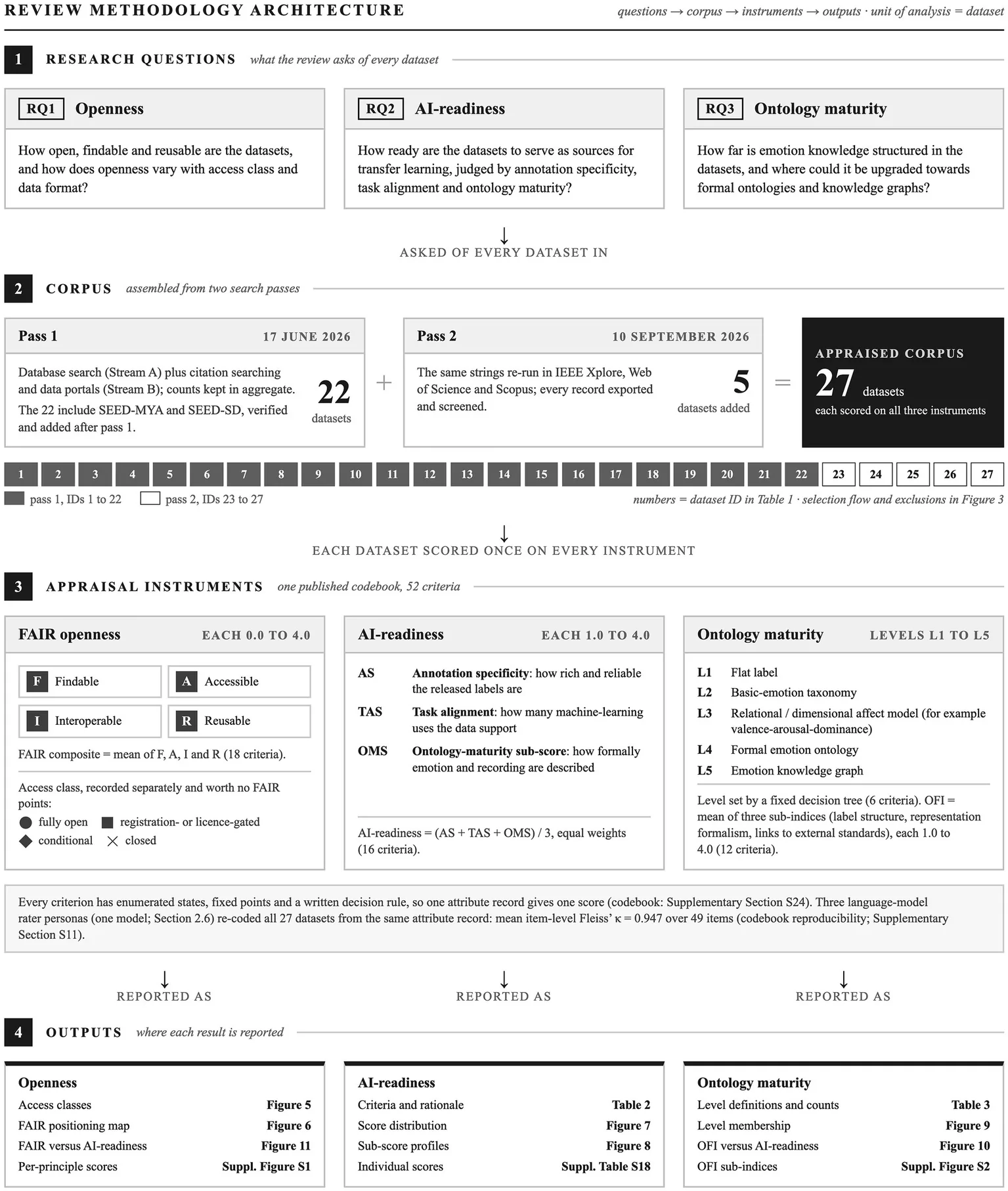
Review methodology architecture. Three research questions, on openness, AI-readiness and ontology maturity, are asked of one corpus assembled in two search passes. Before the second pass the corpus held 22 datasets (IDs 1–22 in Table 1): those kept by the first pass (17 June 2026) and SEED-MYA and SEED-SD, which were verified and added after it. The second pass (10 September 2026) added 5 (IDs 23–27), giving the 27 appraised datasets (selection flow in Figure 3). In the ID strip, filled tiles are IDs 1–22 and outlined tiles the second-pass IDs 23–27. Each dataset is scored once on three instruments defined by one published codebook of 52 criteria (Supplementary Section S24). FAIR openness scores Findability (F), Accessibility (A), Interoperability (I) and Reusability (R), each 0.0–4.0 (18 criteria); access class is recorded separately and contributes no FAIR points. AI-readiness is the unweighted mean of annotation specificity (AS), task alignment (TAS) and the ontology-maturity sub-score (OMS), each 1.0–4.0 (16 criteria). Ontology maturity is a level from L1 to L5 set by a fixed decision tree (6 criteria), complemented by the Ontology Formalisation Index (OFI), the mean of three sub-indices for label structure, representation formalism and links to external standards (12 criteria). Three language-model rater personas (one model; Section 2.6) re-coded all 27 datasets from the same attribute record: mean item-level Fleiss’ *κ* = 0.947 over 49 items (codebook reproducibility; Supplementary Section S11). The figure uses greys only; the access-class shapes in its key (circle fully open, square registration- or licence-gated, diamond conditional) mark datasets in Figures 4–7, 10, 11 and Supplementary Figure S1. The bottom row names the figure or table that reports each instrument’s outputs; the publication-year timeline of the corpus is Figure 4.

**Table 1:** The 27 appraised EEG emotion-recognition datasets, in ID order.

ID	Dataset	Year	Participants	EEG channels	Emotion labels	Task cluster^a^	Access
1	DEAP Koelstra et al. (2012)	2011^*^	32	32	Dimensional: V, A, D, liking (1–9)	Dimensional-VAD	■ Gated^†^
2	SEED Zheng and Lu (2015)	2015	15	62	Discrete: positive, neutral, negative	Discrete-multi-emotion	■ Gated
3	SEED-IV Zheng et al. (2018)	2018	15	62	Discrete: happy, sad, fear, neutral	Discrete-multi-emotion	■ Gated
4	SEED-V Liu et al. (2021)	2022^*^	16	62	Discrete: happy, sad, fear, disgust, neutral	Discrete-multi-emotion	■ Gated
5	SEED-VII Jiang et al. (2025)	2024^*^	20	62	Discrete: six basic emotions plus neutral, with intensity	Discrete-multi-emotion	■ Gated
6	SEED-GER Liu et al. (2022)	2022	8	62	Discrete: positive, neutral, negative	Discrete-multi-emotion	■ Gated
7	SEED-FRA Liu et al. (2022)	2022	8	62	Discrete: positive, neutral, negative	Discrete-multi-emotion	■ Gated
8	DREAMER Katsigiannis and Ramzan (2018)	2017^*^	23	14	Dimensional: V, A, D (1–5)	Dimensional-VAD	■ Gated
9	AMIGOS Miranda-Correa et al. (2018)	2018	40	14	Mixed: V, A, D, basic emotions; external raters	Multimodal	■ Gated^†^
10	MAHNOB-HCI Soleymani et al. (2012)	2011^*^	30 (27 analysed)	32	Mixed: nine emotion keywords plus V, A, D	Multimodal	■ Gated^†^
11	FACED Chen et al. (2023)	2023	123	32	Mixed: nine categories plus 12 self-ratings	Discrete-multi-emotion	■ Gated
12	MPED Song et al. (2019)	2019	23	62	Discrete: joy, funny, anger, fear, disgust, sadness, neutral	Discrete-multi-emotion	■ Gated
13	GAMEEMO Alakus et al. (2020)	2020	28	14	Mixed: four game conditions plus V and A	Wearable-field	● Fully open
14	DENS Mishra et al. (2022)	2021^*^	40 (34 labelled)	129 (128-electrode net plus vertex reference)	Mixed: V, A, D plus marked emotion events	Continuous-naturalistic	● Fully open
15	IESD Onton and Makeig (2022)	2022	34	250 (134–235 retained)	Discrete: 15 imagined emotion categories	Discrete-multi-emotion	● Fully open
16	EmoEEG-MC Xu et al. (2025)	2025	60	64	Mixed: seven categories plus 10 self-ratings	Multimodal	● Fully open
17	EVA-MED Huang et al. (2025)	2025	64	64	Mixed: V and A classes plus SAM ratings	Dimensional-VAD	◆ Conditional
18	EAV Lee et al. (2024)	2024	42	30	Discrete: five categories; self-rated V, A described, not released	Dialogue-social	◆ Conditional
19	Voice-UI Choi et al. (2024)	2024	44 (38 in the benchmark)	63 (57 released)	Dimensional: nine adjective-pair ratings	Dialogue-social	● Fully open
20	Daly (ds002721) Daly et al. (2020)	2020	31	19	Dimensional: eight induced-emotion ratings	Continuous-naturalistic	● Fully open
21	SEED-MYA Aung et al. (2025)	2025	20	62	Discrete: positive, neutral, negative	Discrete-multi-emotion	■ Gated
22	SEED-SD Li et al. (2025)	2025	40	62	Discrete: happiness, sadness, fear, neutral, with intensity	Discrete-multi-emotion	■ Gated
23	Emognition Saganowski et al. (2022)	2022	43	4	Mixed: nine emotion ratings plus V, A, motivation	Wearable-field	■ Gated
24	K-EmoCon Park et al. (2020)	2020	32	1 (band powers)	Mixed: V, A and categories every 5 s	Dialogue-social	◆ Conditional
25	ECSMP Gao et al. (2021)	2021	89 (87 with valid EEG)	7	Discrete: six categories with self-rated intensity	Multimodal	● Fully open
26	LLaMAC Lee and Kim (2025)	2025	108	14	Mixed: V, A, D plus five categories	Multimodal	● Fully open
27	Mixed Emotion Yang et al. (2024)	2024	73	21	Mixed: V, A, D; positive, negative or mixed	Multimodal	◆ Conditional

IDs 1–22 formed the corpus before the second search pass, and IDs 23–27 were added by it (Figure 3). The IDs identify datasets in Figures 2, 4, 5, 7–9 and Supplementary Figures S1, S2; Figures 6, 10, 11 label datasets by the short names given here. Cell shading gives the task cluster, whose colour marks that cluster in Figures 6, 7, 10, 11 and Supplementary Figure S2. Symbols give the access class (● fully open; ■ registration- or licence-gated, shown as gated; ◆ conditional; ^†^ route not functional at the 11 September 2026 re-check). Supplementary tables label the three classes FullyOpen, Academic and Conditional. Year is the publication year of the descriptor record, as in Figure 4 and the adoption ledger of Supplementary Section S22; an asterisk marks the six datasets for which it differs by 1 year from the year of the reference cited under the name. Participants give the cohort in the release, with an analysed, labelled or valid-EEG subset in brackets where the source reports one. EEG channels give the recorded scalp channels, and brackets qualify the count. Emotion labels open with the emotion-model type used in Figure 4 (discrete 12, dimensional 4, mixed 11); V, A and D are valence, arousal and dominance, and SAM is the Self-Assessment Manikin. Full descriptions, including the recorded modalities: Supplementary Table S16. ^a^Task clusters are descriptive labels recorded once in the corpus record; they enter no score, and several datasets share features with another cluster. Continuous-naturalistic: naturalistic stimuli with labels not restricted to one class per trial (participant-marked events in DENS; eight non-exclusive ratings per music excerpt in the Daly deposit). Wearable-field: consumer wearable headsets (Emotiv EPOC+ in GAMEEMO, Muse 2 in Emognition), both recorded during screen-based tasks; DREAMER, AMIGOS, K-EmoCon and LLaMAC also use consumer headsets. Multimodal: EEG released with several synchronised peripheral or video streams; DEAP, MPED, DENS, EVA-MED, EAV, Voice-UI, Emognition and K-EmoCon also release several such streams. Dimensional-VAD: labels chiefly on the valence and arousal dimensions, with dominance in DEAP and DREAMER (EVA-MED has no dominance rating). Dialogue-social: conversation or voice-interface dialogue. Discrete-multi-emotion: discrete emotion categories as the main target (FACED also releases dimensional ratings). Persistent identifiers of all 27 datasets are listed in Supplementary Table S5. Identifier audit: 41 of the 42 persistent identifiers associated with the 27 corpora resolve in at least two of Crossref, DataCite and OpenAlex with a matching title; the K-EmoCon Zenodo deposit (10.5281/zenodo.3931963) resolves in DataCite alone (Supplementary Section S20).

### 2.2 Search and selection

Datasets were identified through two additive streams in two search passes (Figure 3). Stream A searched bibliographic databases and registers; Stream B browsed data portals and followed citations. Each step is summarised below and recorded in full in the Supplementary section named.

- *Database searching (Stream A)*. Search strings combined EEG, affect and dataset terms, adapted to each database. The first pass (17 June 2026) searched six databases and registers and kept aggregate counts only. The second pass (10 September 2026) reran the strings in IEEE Xplore, Scopus and Web of Science, with a document-type limit in each database, and exported every record. It admitted five datasets once their access routes were verified: Emognition (Saganowski et al., 2022), K-EmoCon (Park et al., 2020), ECSMP (Gao et al., 2021), LLaMAC (Lee and Kim, 2025) and Mixed Emotion (Yang et al., 2024). Seven further candidates were deferred because their access routes had not been verified (Section 3.1). Strings, limits and counts are in Supplementary Table S1 and Section S4b.
- *Data portals and repositories (Stream B)*. To find corpora released without a conventional paper, we browsed OpenNeuro/NEMAR, Zenodo, Mendeley Data, IEEE DataPort, Kaggle, Hugging Face Datasets, Papers With Code and GitHub. Each visit was logged following PRISMA-S item 4 (Rethlefsen et al., 2021). Portal browsing contributed three datasets that Stream A had not returned (Supplementary Sections S2, S7).
- *Citation searching (Stream B)*. We traced citations forward and backward from seed papers. Totals returned by citation services count citation links (edges), not records, so they were never used as record counts. The edge counts were retrieved on 25 September 2026, because the counts at the time of the first search pass were not recorded. Citation searching contributed three further datasets (Supplementary Sections S3, S3b, S8).
- *Deduplication and screening*. Records were merged into one scored unit when they described the same corpus: several papers about one corpus, a mirror of a deposit, or companion deposits that the corpus descriptor presents as one data release. The Daly family of OpenNeuro deposits, for example, is scored through ds002721. Corpora that share a descriptor paper but are released as separately named datasets with their own participants, such as SEED-GER and SEED-FRA, stay separate units. Stream A was enumerated first, and Stream B contributed only resources that Stream A had not returned. A resource was retained whenever its eligibility could not yet be excluded. One resource can be counted at more than one screening step, so Supplementary Sections S4, S4b reconcile the counts with distinct resources.
- *Exclusions*. The exclusion log records 11 resources that were excluded or held, each with a reason code, and none of them enters any score (Supplementary Sections S0, S6).
- *SEED family and one held resource*. Each SEED variant was judged separately. The eight emotion-labelled variants are included, and the vigilance and perception variants are excluded because they carry no emotion labels. EEGEMO is held and not scored: its files are restricted, and no procedure for obtaining access is published (Supplementary Sections S6, S20.1).

**Figure 3:**
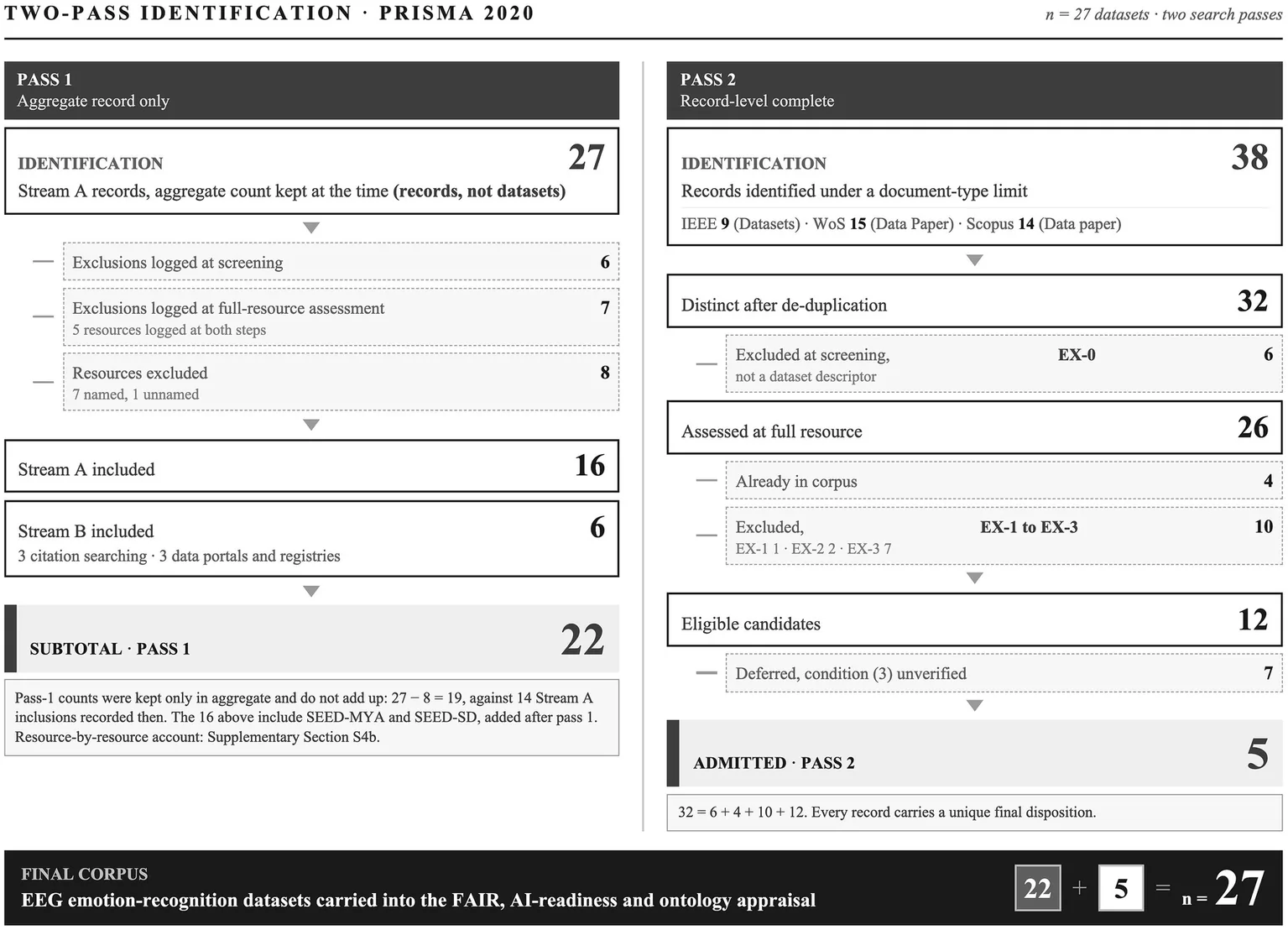
Two-pass identification of the 27 appraised datasets (PRISMA 2020). First pass (17 June 2026): the search record keeps only aggregate counts, shown as recorded; its Stream A figure of 27 counts records, not datasets, and is unrelated to the 27 appraised datasets. Before the second pass the corpus held 22 datasets: 16 from database searching (Stream A, including SEED-MYA and SEED-SD, which were verified and added after the first pass) and 6 from citation searching and data portals (Stream B, 3 each). Second pass (10 September 2026): under the document-type limits of Supplementary Table S1, the database searches returned 38 records (IEEE Xplore 9, Web of Science 15 and Scopus 14). After deduplication, 32 distinct records remained: 6 excluded at screening (EX-0, not a dataset descriptor), 4 already included, 10 excluded at full-resource assessment and 12 eligible candidates. Five candidates were admitted after access verification and 7 were deferred with condition (3) unverified; the record reconciles as 32 = 6 + 4 + 10 + 12. Final corpus: 22 + 5 = 27, drawn as a filled and an outlined box, as in Figure 2; dashed side boxes show what left the flow at each step. The first-pass counts cannot be fully reconciled; Supplementary Section S4b gives the resource-by-resource account, and data/S4b_pass2_records.csv in the reproducibility package lists the 32 second-pass records.

Locating the datasets was itself difficult; Section 2.5 describes the checks this required.

### 2.3 Eligibility and risk of bias

Eligibility turns on whether a dataset is publicly obtainable. Publicly obtainable means that a dataset has a persistent public record and can be obtained by eligible researchers worldwide under stated terms. It does not necessarily mean unrestricted download, redistribution or commercial reuse.

A resource was included when it met the three conditions of the D facet (Figure 1) jointly. Condition 1 requires scalp EEG with emotion or affect labels, whether discrete emotion categories or dimensional affect ratings (for example valence, arousal and dominance). Condition 2 requires persistent documentation (Section 1). The access condition (condition 3) requires a dataset-level persistent identifier that resolves to the data or, failing that, a published request procedure that any researcher can complete. A free account, a signed licence, a subscription that can be bought from anywhere and a disclosed approval step all pass. Only a route restricted by nationality or region, or the absence of any documented route, fails the test. Closed resources were removed before appraisal, so the absence of closed datasets is a property of the sampling frame, not a finding about the field.

The access routes of the 22 datasets held before the second search pass were checked on 1 August 2026, and the routes of all 27 were checked on 11 September 2026. At the second check, the documented routes of DEAP, AMIGOS and MAHNOB-HCI no longer worked. All three are retained and flagged in Supplementary Table S17, because eligibility when the corpus was assembled and the state of a route on a later date are separate facts. Supplementary Section S13 applies the conditions to each dataset, and Section S20 gives the route evidence and check dates.

Risk of bias was appraised with a QUADAS-2-inspired matrix (Whiting et al., 2011). This is an adaptation for dataset appraisal, not a validated use of QUADAS-2. Each dataset was rated Low, Moderate or High on four domains: D1 dataset construction, D2 annotation, D3 documentation and transparency, and D4 accessibility and reproducibility. A further judgement rates applicability to the spatial-emotion question (Supplementary Section S12). The D4 rule grades access gating, licence, identifier and format, and has no level for a route found non-functional. DEAP, AMIGOS and MAHNOB-HCI are therefore rated Moderate on D4 as registration- or licence-gated corpora, and their route failures are disclosed in Supplementary Section S12.1. Reporting bias, the subject of PRISMA 2020 items 14 and 21, takes a specific form here. Selective outcome reporting does not arise, because the appraised quantities are properties of the released resources. Its analogue, selective documentation by dataset releasers, is assessed in D3.

### 2.4 Appraisal instruments

The attributes of each dataset, including participants, montage, label model, modalities, access class, licence, format and identifiers, were extracted into one source-verified record (AI assistance and human checks: Section 2.6). Each value in the record carries its source and check date, with the exceptions listed in Supplementary Section S24.1.

One published codebook turns the record into scores (Supplementary Section S24). It has 52 criteria, and each criterion has enumerated states, fixed points, a decision rule and a missing-data policy, so one record gives one score. Scripts in the reproducibility package recompute every score and score-based summary statistic from the coded states. Choosing a state from the record is the coding step, whose reproducibility Section 2.5 reports.

Two conventions apply to every index. A property that cannot exist for a design is set aside, whereas one that could exist but is not reported scores the floor, so reporting less never raises a score. Each index is rounded once, at the end, with Python’s round() function, which sends an exact halfway value to the even digit (2.25 becomes 2.2).

#### 2.4.1 FAIR openness and access class

Openness was scored against the FAIR principles (Wilkinson et al., 2016), from 0.0 to 4.0 for each principle. Findability (F) asks whether a dataset-level identifier exists, resolves and is unique, and whether a descriptor record is registered. Accessibility (A) asks whether the access route and the eligibility requirements are clearly described, open to researchers in any country and working on the check date; free download is not required. Interoperability (I) ranks the container of the signal files (standard EEG formats such as BIDS-EEG, EDF or EEGLAB, then generic MATLAB or text files, then device-native binaries) and credits standard label files and loaders. Reusability (R) reads the licence, reuse terms, variable-level documentation and provenance. The criteria are in Supplementary Section S24.2 and the scores in Supplementary Table S17.

Access class was recorded on a separate axis, checked against the live repository. Fully open means anonymous self-service download; registration- or licence-gated means a free account, an institutional approval or an end-user licence agreement; conditional means a data-use agreement or a discretionary approval decision. Access class adds no FAIR points, because a well-documented dataset can still require registration and an open download can still be poorly described. Keeping the two apart also lets Section 3.5 test whether openness and readiness go together.

#### 2.4.2 AI-readiness

AI-readiness scores how useful a dataset is as a source for transfer learning, judged from what the release contains, not from reported accuracy. A reuser who pre-trains a model on the dataset and adapts it to new data needs three things. The labels must be ones a model can learn from, the release must run standard tasks without being rebuilt, and its labels and channels must line up with another dataset. The three sub-scores measure these three conditions. The criteria follow one principle: each names something a reuser must find in the release to complete one step of that work, such as loading the files, reading the labels, matching the channels or splitting the records by participant. Table 2 says in plain words why each condition matters.

**Table 2:** AI-readiness criteria, component definitions and applied weights.

Criterion	What it measures	Component inputs	Weight	Scale	Why it matters for transfer
AS (Annotation Specificity)	Richness and reliability of the released emotion labels	Label type + rater design + labelled units per participant + inter-annotator agreement, each scored on enumerated criteria, summed, then normalised affinely onto the scale. Shares of the raw maximum: label type 41.7% (label scheme plus temporal granularity), rater design 25.0%, labelled units per participant 16.7%, inter-annotator agreement 16.7%. Where one rater per released item makes agreement undefinable, that criterion leaves the denominator and the other three shares rescale	1/3 exact	1.0–4.0	A model can learn only what the labels say. Richer label schemes carry more information per trial, and labels resolved in time allow time-resolved learning. A participant’s own report says what was felt, and several independent annotators allow the labels to be checked. Per-person models need enough labelled units from each participant, and published agreement shows how reliable the labels are.
TAS (Task Alignment)	Breadth of machine-learning use the corpus can support	Supported-task breadth (classification and dimensional regression, continuous tracking, multimodal fusion, cross-subject and cross-corpus transfer) + benchmark-split availability + training-framework support, each scored on enumerated criteria, summed, then normalised affinely onto the scale	1/3 exact	1.0–4.0	Transfer works only if the dataset plugs into standard tasks and tools. A numeric affect scale allows regression, labels within a trial allow tracking over time, and a time-locked second signal allows fusion. Records from at least 10 participants allow cross-subject tests, and standard labels and electrodes shared with other corpora allow cross-corpus tests. A documented split with runnable code makes results comparable, and a reader in an existing EEG software library (TorchEEG, EEGain, braindecode or MNE) removes bespoke parsing.
OMS (Ontology-Maturity Sub-score)	Formality of the emotion representation	Representation structure + montage nomenclature and density + BIDS-EEG compliance + controlled vocabulary, each scored on enumerated criteria, summed, then normalised affinely onto the scale	1/3 exact	1.0–4.0	A source dataset helps a new study only if its labels and channels can be lined up with the new data. Structured labels and a controlled vocabulary state what each label means. Standard electrode names with positions, and a montage dense enough to be reduced to a sparser target cap, let the channels be matched. BIDS-EEG organisation lets software read both datasets in the same way.
Composite	AIR = (AS + TAS + OMS)/3	Equal weighting asserts no priority among the criteria	1.000	1.0–4.0	No evidence ranks the three conditions, so each counts equally.

Each sub-score is rated 1.0–4.0 and the composite is their unweighted mean. Within each sub-score the named inputs are scored on enumerated criteria carrying fixed points, the points are summed, and the sum is mapped affinely onto the 1.0–4.0 scale; the percentages shown for AS are each input’s share of the raw maximum, and the full point ladders, decision rules and missing-data policies for all three sub-scores are given in Supplementary Section S24. The last column says in plain words why each condition matters when a dataset is used as a source for transfer learning. Each composite is reported with its rank and its three sub-scores in Supplementary Tables S15, S18. Equal weighting asserts no priority among the three criteria; the rank ordering of the 27 datasets is stable under ±0.10 perturbation of these weights (Spearman *ρ* ≥ 0.982, worst case 13 discordant pairs of 351; Supplementary Section S14.1).

Annotation Specificity (AS) scores the released labels. It credits a rich label scheme, labels resolved finely in time, labels reported by participants or cross-checked by several annotators, enough labelled units per participant, and published agreement between annotators. The Task Alignment Score (TAS) scores six checks that a transfer pipeline needs. Three ask which tasks the labels and signals support: regression on a numeric affect scale, tracking with labels resolved within a trial, and fusion with a second signal time-locked to the EEG. Three ask whether the data can enter a transfer pipeline: records from at least 10 participants with a label target shared with other corpora, a documented split with runnable code, and an existing reader. The reader must belong to an open-source EEG library (TorchEEG, EEGain, braindecode or MNE). The Ontology-Maturity Sub-score (OMS) scores whether labels and channels can be aligned with another dataset: label structure, standard electrode names with positions, channel density, BIDS-EEG organisation and a controlled vocabulary. Each sub-score maps its criterion points linearly onto 1.0–4.0. AI-readiness is their unweighted mean, AIR = (AS + TAS + OMS)/3 (Table 2; Supplementary Section S24.2).

Equal weights are a no-preference default, because no evidence ranks the three conditions, and the ordering is stable when any weight moves by 0.10 (Section 3.5; Supplementary Section S14). Section 4.2 sets out why the score should indicate suitability for transfer and what supports the instrument. Scores are relative positions under published criteria, not validated thresholds. Top states that no dataset reaches are kept, so that the gap stays visible (Supplementary Section S14.2).

#### 2.4.3 Ontology maturity

Ontology maturity describes how formally a dataset structures its emotion labels. Table 3 defines its five levels: L1 flat label, L2 basic-emotion taxonomy (named emotion categories), L3 relational or dimensional affect model (for example valence-arousal-dominance), L4 formal emotion ontology and L5 emotion knowledge graph.

**Table 3:** Five-level ontology-maturity model for EEG emotion-recognition datasets, with operational definitions and the corpus distribution (*n* = 27).

Level	Name	Operational definition (EEG emotion datasets)	Datasets, *n* (%)
L1	Flat label	Bipolar or categorical emotion labels with no internal structure (e.g., positive/neutral/negative)	4 (14.8%)
L2	Basic-emotion taxonomy	Hierarchical discrete categories (is-a), such as happy/sad/fear/disgust/neutral	7 (25.9%)
L3	Relational/dimensional affect model	Coordinates in a dimensional affect model (for example valence-arousal-dominance) or explicit inter-label relations	16 (59.3%)
L4	Formal emotion ontology	Explicit emotion ontology, formal semantic mapping to one, or equivalent machine-interpretable relation model over the affect labels (a standard data schema alone does not qualify)	0 (0.0%)
L5	Emotion knowledge graph	Instance-linked, reasoning-capable affect graph relating emotion, stimulus and signal	0 (0.0%)

Levels are assigned by the decision tree published in Supplementary Section S24, which reads the released label model and the presence of a vocabulary binding and returns the highest level whose test the dataset passes. The realised range in this corpus is L1–L3; L4 and L5 are empty, which is a finding about the appraised corpus and not a limit of the instrument, and the scale should not be read as one with five occupied levels.

The criteria follow one principle: levels are ordered by the mappings they allow, and a dataset takes the highest level whose test it passes. An L1 label, such as positive, neutral or negative, maps onto one bipolar judgement. L2 names emotion categories that can be matched across corpora by name. L3 relates labels to one another, usually as coordinates on affect dimensions, so labels from different corpora and ratings collected in a building can be placed side by side. L4 declares these relations in a machine-readable ontology, so software can carry out and check a mapping. L5 links emotion, stimulus and signal instances in a queryable graph.

The L3 test has three routes: a released affect dimension such as valence, graded ratings on nine or more affect terms, or more than eight discrete categories. The threshold is eight because the largest standard basic-emotion set has eight categories, so a larger inventory is read as naming blends or intensities of those terms (Supplementary Section S24.2). This is the weakest of the three routes: the relations between categories are inferred from the size of the inventory, not released with it, and Section 3.4 shows what this means for a reuser. Two rules decide most borderline cases. A rating counts only if it is released aligned to the records; ratings described only in a paper do not count. An intensity attached to a category is not an affect coordinate.

A fixed decision tree assigns the level from the released label model and any binding to an external vocabulary, so no level is entered by hand. BIDS organises files but does not say what labels mean, so it is credited in FAIR Interoperability and OMS, not in the level. Because five levels are coarse, the Ontology Formalisation Index (OFI) measures upgrade potential more finely. It is the mean of three sub-indices, each 1.0–4.0, for label structure, representation formalism and links to external standards (Supplementary Sections S19, S24.2).

### 2.5 Verification of identifiers, coverage and codebook reproducibility

Finding the datasets was a large part of the work. One corpus can be described in several papers, deposited under several identifiers, mirrored with different metadata, or reachable only through a laboratory web page. Run again, the recorded search strings found five eligible corpora that the first pass had not carried forward. Three documented access routes stopped working between two checks. Supplementary Sections S4b, S20, S21 record each problem and how it was handled.

Every persistent identifier of the 27 corpora (each descriptor record and any dataset deposit) was submitted to Crossref, DataCite and OpenAlex. An identifier counted as verified when it resolved in at least two of the three with a matching title. The audit separates a descriptor identifier, which points to the paper, from a dataset-level identifier, which points to the data. It records the absence of the latter and checks whether two corpora share one identifier (Supplementary Section S20; results in Sections 3.1 and 3.2).

To check coverage, the 27 datasets were matched against a landscape of 102 affective-EEG resources. The authors compiled it separately, with a different protocol and a cut-off of 31 July 2026, so it is not independent. Its unmatched units were screened against the eligibility conditions of Section 2.3 (Section 4.4; Supplementary Section S21).

Three language-model rater personas (one model; Section 2.6) tested whether the rules of the codebook are reproducible. Working in separate contexts from one fixed attribute record, with point values masked, each selected a state for every criterion of all 27 datasets. Mean item-level Fleiss’ *κ* was 0.947 over the 49 items with defined coefficients, with a percentile-bootstrap 95% interval of 0.918–0.963 (Landis and Koch, 1977). The estimate describes codebook reproducibility given one shared record. It does not measure human inter-rater reliability or the reliability of extraction (Supplementary Section S11; Section 4.6).

### 2.6 Use of AI tools

We used two generative AI tools, Anthropic Claude and OpenAI Codex. Claude, accessed through the Claude Code environment (2026), extracted the attribute record. It ran as several agents, separate instances of the model with defined roles, each of which recorded the source of every value it extracted, except for the fields listed in Supplementary Section S24.1. Claude also applied the three appraisal instruments under the codebook and served as the three language-model rater personas of Section 2.5. OpenAI Codex was used for language editing of an earlier draft of this manuscript. Claude (Claude Code) assisted with condensing and reorganising the text, revising figure layouts, building the reproducibility package and preparing the correspondence with the journal. The authors designed the review and its eligibility rules. They fixed the codebook and the reproducibility protocol before the three-rater coding, verified the agent outputs against the cited primary sources, and take responsibility for all content and reported values. The rater codings are published in full (Supplementary Section S11).

## 3 Results

### 3.1 The appraised corpus

Table 1 lists the 27 appraised datasets with their year, participants, EEG channels, emotion labels, task cluster and access class. IDs 1–22 formed the corpus before the second search pass and IDs 23–27 were added by it; the figures identify datasets by these IDs or by the short names of Table 1. The designs vary widely: cohorts run from 8 participants (SEED-GER and SEED-FRA) to 123 (FACED), and montages from a single channel (K-EmoCon) to 250 (IESD). Each dataset also carries one of six task-cluster labels (Table 1). The labels are descriptive: they were assigned once in the corpus record, several datasets share features with another cluster (Table 1, footnote a), and they enter no score. Discrete-multi-emotion is the largest cluster with 11 datasets, followed by multimodal (6), dimensional-VAD (3), dialogue-social (3), continuous-naturalistic (2) and wearable-field (2).

The corpus is less independent than its count suggests. The SEED line alone supplies 8 of the 27 datasets, all from one laboratory and all recorded on the same 62-channel montage. Section 3.5 tests whether this concentration changes any result.

Figure 3 shows how the corpus was assembled. Before the second pass the corpus held 22 datasets: 16 from database searching, including SEED-MYA and SEED-SD, which were verified and added after the first-pass census, and 6 from citation searching and portals. Citation searching yielded MPED, GAMEEMO and EAV. Portal and repository searching yielded IESD, EmoEEG-MC and the Daly deposit ds002721, whose companion deposit ds002722 is recorded in Supplementary Section S20 but not scored. The second pass, which reran the database searches only, added 5 datasets (Section 2.2). Their retrieval at the second pass demonstrates that the first-pass enumeration was incomplete. Seven further second-pass candidates passed the signal and label gates but were deferred, not appraised, because the access condition could not be verified. The first-pass counts are aggregates that cannot be fully reconciled; Supplementary Section S4b gives the resource-by-resource account.

A total of 11 resources were removed and recorded with a reason (Supplementary Section S6). Four carry no scalp EEG: CASE (Sharma et al., 2019), WESAD (Schmidt et al., 2018), VREED (Tabbaa et al., 2021) and MVRS (Mousavi and Ilanloo, 2025). Two use another neuroimaging modality: magnetoencephalography in DECAF (Abadi et al., 2015) and functional magnetic resonance imaging in NeuroEmo (Abgeena et al., 2025). Three SEED-line variants carry vigilance or perception labels and no emotion labels: SEED-VIG (Zheng and Lu, 2017), SEED-VLA/VRW (Luo et al., 2024) and SEED-DV (Liu et al., 2024). EEGEMO (Moontaha et al., 2022) is held because universal access could not be confirmed, and one further name denotes no distinct corpus.

The persistent identifiers that the corpora do have are sound. The identifier audit resolved 41 of the 42 persistent identifiers associated with the 27 corpora in at least two of Crossref, DataCite and OpenAlex, with no conflicting title. The one exception, the K-EmoCon Zenodo deposit (10.5281/zenodo.3931963), resolves in DataCite alone. The weakness lies in identifiers that were never minted, which Section 3.2 reports (ledger: Supplementary Section S20).

Fully open release begins only in 2020, and the timeline shows no later move towards open, standard-format release (Figure 4). The Year shown in Figure 4 and Table 1 is the publication year of the corpus descriptor record, the dated reference by which the corpus is cited; it is not the repository release year. IESD shows the difference, with an acquisition dating from 2009 but a descriptor record from 2022 (Supplementary Section S16). On this definition the 27 datasets span 2011–2025. Every release up to 2019, the seven corpora from DEAP and MAHNOB-HCI (2011) to MPED (2019), is a registration- or licence-gated.mat/.bdf corpus. From 2020 onwards fully open and registration- or licence-gated releases appear side by side. Only 3 of the 10 releases from 2023 to 2025 are fully open, about the same share as in the corpus as a whole. A validated standard structure holds for only two of the 27, EmoEEG-MC (2025) and the Daly deposit (2020).

**Figure 4:**
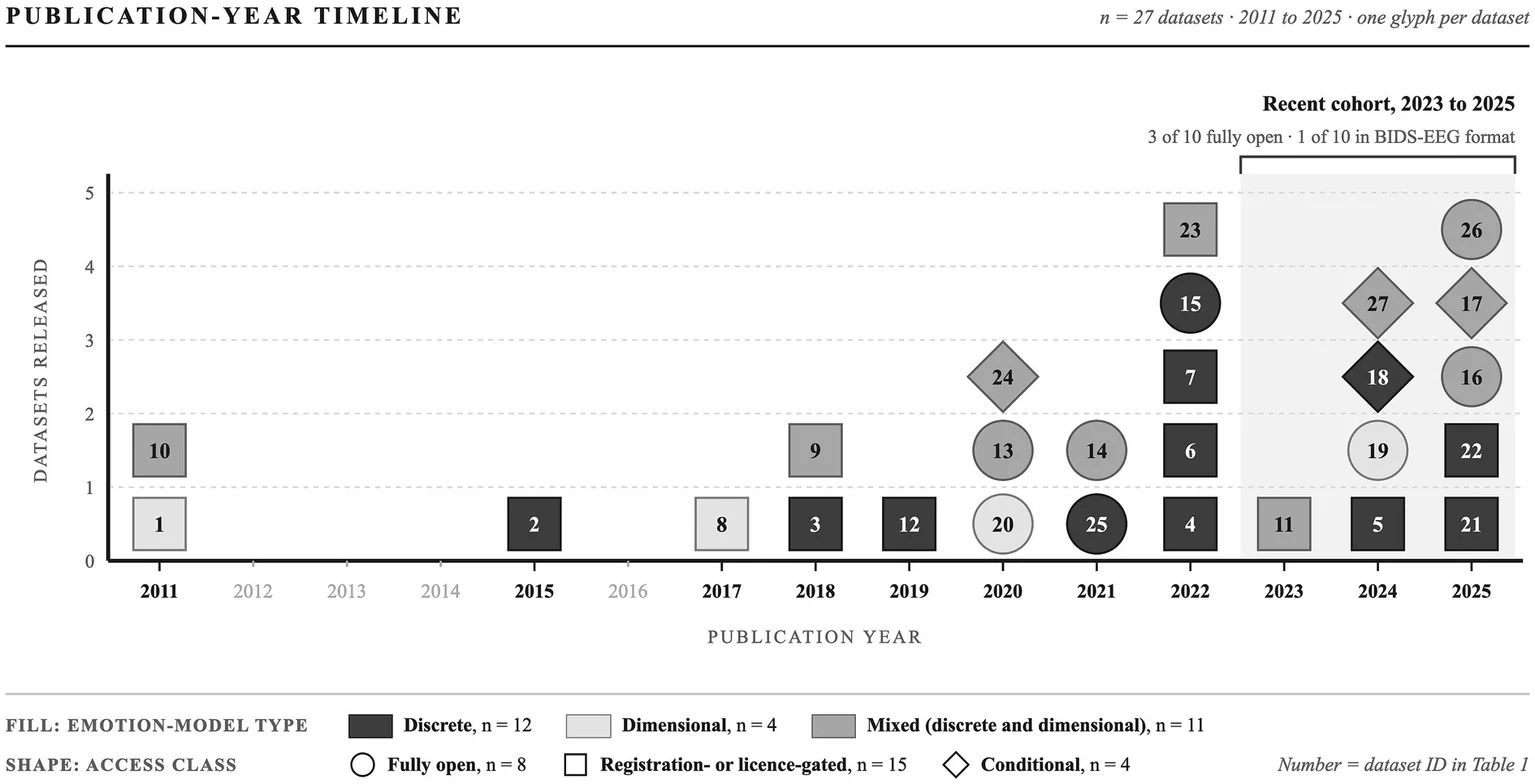
Publication-year timeline of the 27 appraised datasets (2011–2025; year of the citable descriptor record, as in Table 1 and the adoption ledger of Supplementary Section S22). Each glyph is one dataset, numbered by its ID in Table 1, and each column holds the datasets whose descriptor record appeared in that year. Glyph shape gives the access class (circle, fully open, *n* = 8; square, registration- or licence-gated, *n* = 15; diamond, conditional, *n* = 4). Glyph fill gives the emotion-model type that opens each Emotion labels entry of Table 1: dark grey, discrete, *n* = 12; light grey, dimensional, *n* = 4; mid grey, mixed discrete and dimensional, *n* = 11. Dimensional labels are ratings on valence-arousal-dominance or on study-specific scales such as the nine adjective-pair ratings of Voice-UI. Fills are grey because colour is reserved for the task cluster. In the recent cohort (2023–2025, grey band), 3 of the 10 releases are fully open and 1 is distributed in BIDS-EEG format.

### 3.2 Openness (RQ1)

Access classes describe what a researcher must do to obtain a corpus, not what the corpus supports once obtained. The distribution is fully open 8 (29.6%), registration- or licence-gated 15 (55.6%), conditional 4 (14.8%) and closed 0 (Figure 5); closed resources were excluded at eligibility (Section 2.3). The four conditional datasets are EAV, which requires a data-use agreement, EVA-MED, whose current version is released only on approved request, and K-EmoCon and Mixed Emotion, each released under an approval step of its own.

**Figure 5:**
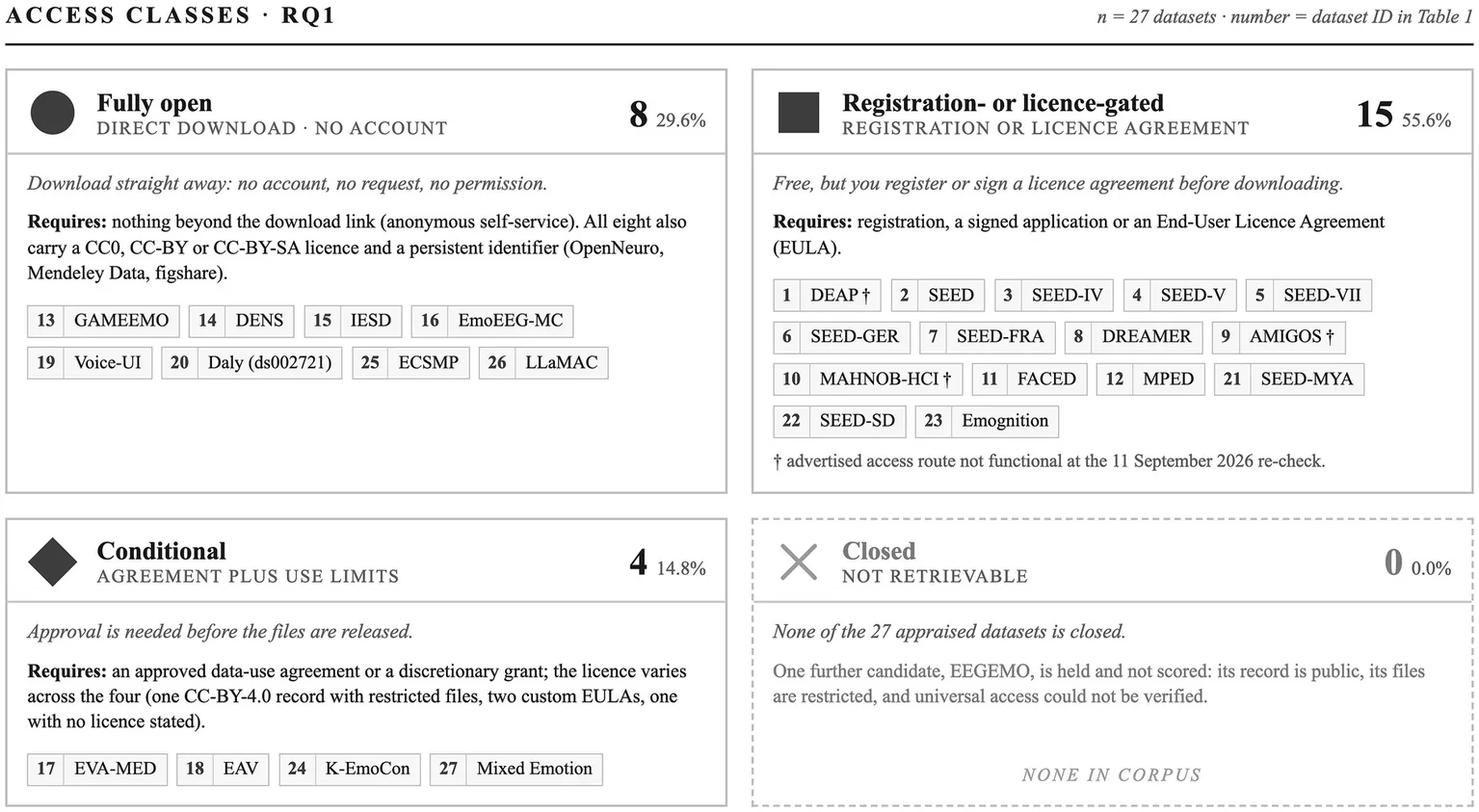
Access classes of the 27 appraised datasets; each card states what the class requires and lists its members by their ID in Table 1. Fully open 8 (29.6%), registration- or licence-gated 15 (55.6%), conditional 4 (14.8%) and closed 0 (0.0%). Eligibility required a documented route grantable irrespective of nationality or region, so all 27 routes met that condition when eligibility was assessed. The advertised routes of DEAP, AMIGOS and MAHNOB-HCI (dagger) were not functional at the 11 September 2026 re-check (Section 2.3). One further candidate, EEGEMO, is held and not scored. The class is shown by shape alone (circle, square, diamond, and a cross for the empty closed class), without colour. Access class is recorded separately from the FAIR score and contributes no points to it; the same shapes mark access class in Figures 4, 6, 7, 10, 11 and Supplementary Figure S1.

The corpora are easy to find and to request but hard to combine. Corpus means were *F* = 3.311, A = 3.511, I = 2.044 and R = 2.563, with a composite of 2.857 (per-dataset scores in Supplementary Table S17). Interoperability is weakest because the usual release is a MATLAB .mat file or another non-BIDS format. Only EmoEEG-MC and the Daly deposit validate against BIDS-EEG; DENS and IESD are partially compliant, and no dataset reaches the top Interoperability score (Supplementary Figure S1). Interoperability follows the format of a deposit, not its access class: FACED, which is registration- or licence-gated, and GAMEEMO, which is fully open, both score I = 1.5.

A missing licence counts against a dataset, because a corpus that states no licence is not thereby permissive. DREAMER, FACED and EVA-MED state no data licence at their deposits, and they hold the three lowest Reusability scores in the corpus. Licence and access are separate attributes: EAV carries a CC-BY-4.0 licence yet conditional access (Supplementary Section S20.1).

The four OpenNeuro deposits, EmoEEG-MC, the Daly deposit, IESD and DENS, hold the top of the FAIR ordering, and all four sit in a repository that enforces a data standard (Figure 6). Openly licensed releases held elsewhere score lower. Every fully open dataset has a higher FAIR composite than every dataset in the other two classes (Supplementary Figure S1). This separation is partly built in. Access class adds no FAIR points, but the open licence that every fully open release carries also counts towards its Accessibility and Reusability scores (Supplementary Section S23).

**Figure 6:**
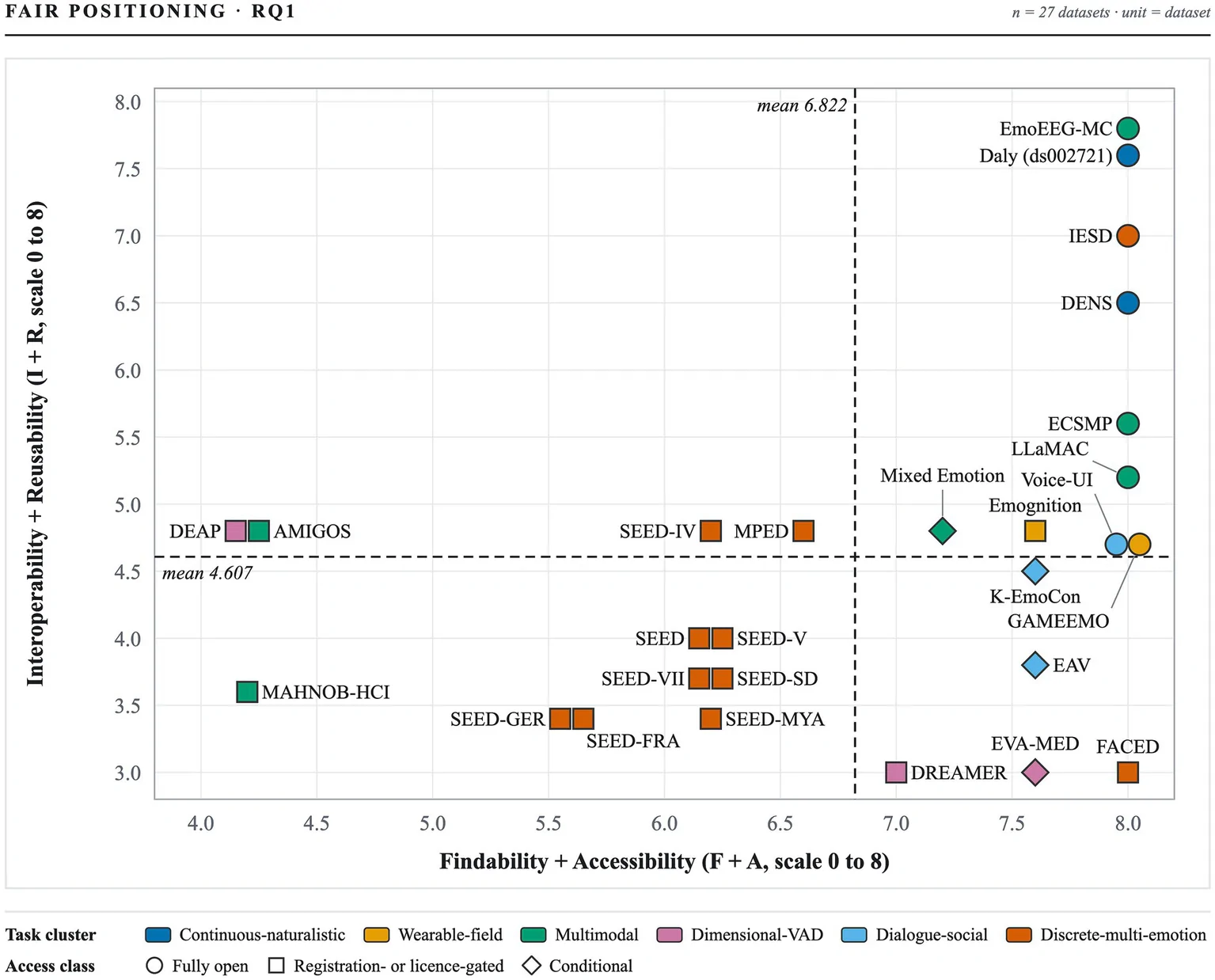
FAIR positioning of the 27 datasets: Findability + Accessibility (F + A) against Interoperability + Reusability (I + R). Each axis is the sum of two FAIR principles scored 0.0–4.0 (corpus means F = 3.311, A = 3.511, I = 2.044 and R = 2.563). Fill shows the task cluster and shape the access class (circle fully open, square registration- or licence-gated, diamond conditional), as listed in Table 1. Quadrants are not shaded, and the axes show the occupied part of the 0–8 range. Dashed lines mark the corpus means (F + A 6.822; I + R 4.607). Ten datasets lie above both means: all 8 fully open releases, Emognition and Mixed Emotion; 14 of the 15 registration- or licence-gated corpora lie outside that quadrant. Five pairs share identical coordinates (DEAP and AMIGOS, SEED and SEED-V, SEED-VII and SEED-SD, SEED-GER and SEED-FRA, Voice-UI and GAMEEMO) and are drawn side by side, centred on the shared value (display offset 0.05 on the F + A axis).

More-cited corpora tend to be less open. Adoption was measured as the OpenAlex citation count of each descriptor record on 12 September 2026 (Supplementary Section S22). Every corpus has an indexed record; IESD, which has no descriptor paper, is counted through its OpenNeuro accession. Adoption correlates negatively with FAIR (Spearman *ρ* = −0.504 on raw counts and −0.571 on counts per year since publication). It shows no material association with AI-readiness (*ρ* = +0.176, *p* = 0.38, and +0.203, *p* = 0.31). Split at the median citation count, the more-cited half also has the lower mean FAIR composite. The negative association survives normalisation by age, so it is not only an effect of older corpora deposited under laxer publisher data-sharing requirements. A correlation across 27 corpora at one check date establishes no mechanism.

Nearly half of the corpus, including its most-cited members, can be reached only through a descriptor paper or a laboratory web page. In all, 13 of the 27 corpora (48.1%) have no dataset-level persistent identifier: DEAP, the eight SEED variants, DREAMER, AMIGOS, MAHNOB-HCI and MPED. Their documented routes rely on descriptor-paper identifiers or laboratory landing pages; the Zenodo record associated with DREAMER carries the journal article’s DOI. All five datasets added at the second pass have a dataset-level identifier, so all 13 are first-pass corpora. Recency does not close the gap: SEED-MYA and SEED-SD, both described in 2025, also lack one. SEED-GER and SEED-FRA share a single descriptor DOI and are told apart only by their laboratory pages.

Three of these corpora, DEAP, AMIGOS and MAHNOB-HCI, could not be reached through their published routes at the 11 September 2026 re-check (Section 2.3). Each therefore scores A = 1.6, the lowest Accessibility in the corpus, and none has a dataset-level identifier to fall back on (Supplementary Table S17 and Section S20.1). Section 4.1 discusses what this means for the durability of the benchmark literature.

Access class is also a poor guide to what a corpus offers or allows. The two richest annotation records belong to AMIGOS, which is registration- or licence-gated, and K-EmoCon, which is conditional. Obtaining a corpus also does not mean that its data can be shared. The end-user licences of DEAP, AMIGOS and the SEED family permit academic analysis but forbid distributing the dataset or portions of it, except small portions used to illustrate publications. Section 3.5 tests whether openness tracks readiness across the whole corpus.

### 3.3 AI-readiness (RQ2)

AI-readiness scores sit in the middle of the scale: mean 2.684, median 2.60, range 2.10–3.33 (Figure 7; individual scores in Supplementary Tables S15, S18). No current release can reach the top of the scale. The top states of the codebook require a controlled vocabulary or a formal emotion ontology for the labels, and no dataset has either (Supplementary Section S14.2). Of the three conditions that AI-readiness scores, alignment is the weakest. The corpus mean of OMS is below those of AS and TAS (Figure 8 gives all three), and OMS is lower than AS in all but five of the 27 datasets.

**Figure 7:**
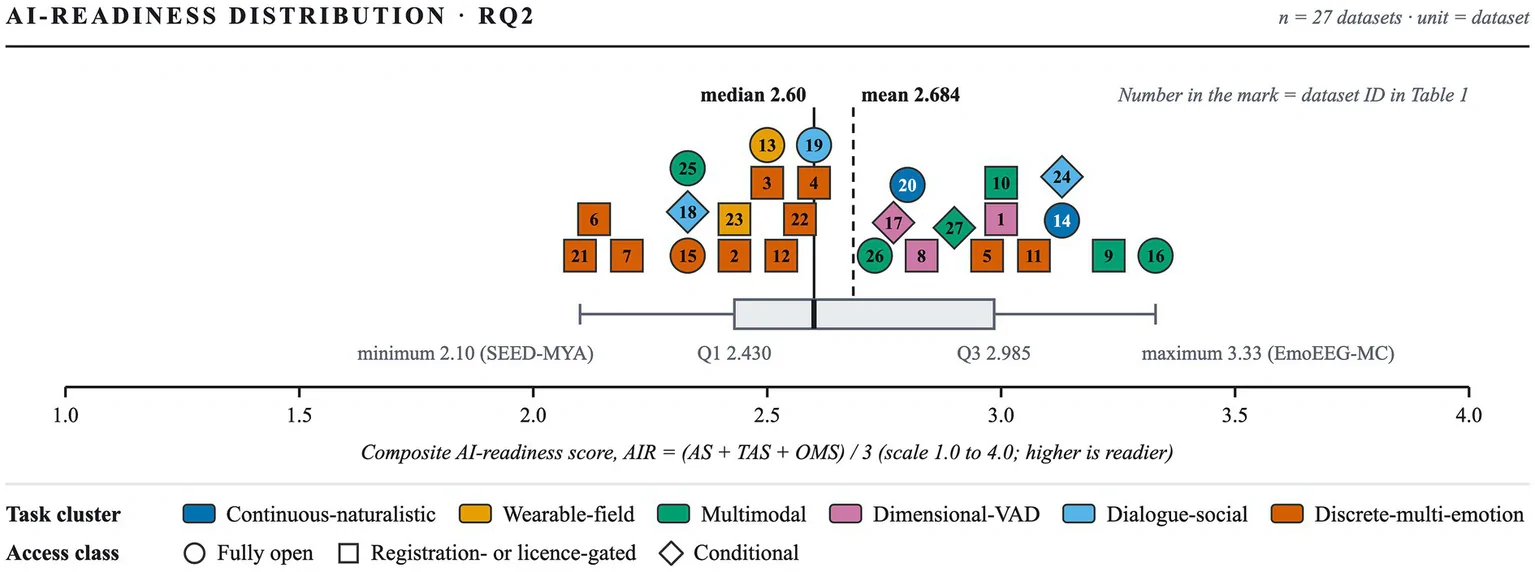
Distribution of the composite AI-readiness score (AIR = (AS + TAS + OMS)/3, scale 1.0–4.0) across the 27 datasets. Each mark is one dataset, placed by its score and numbered by its ID in Table 1; fill shows the task cluster and shape the access class, as in Figure 6. Marks with close or equal scores are stacked, and their height carries no value. Solid line, median; dashed line, mean: mean 2.684, median 2.60, range 2.10–3.33. The grey box spans the first to third quartile (2.430–2.985; linear interpolation between order statistics) and the whiskers the full range. Individual scores and ranks are listed in Supplementary Tables S15, S18.

**Figure 8:**
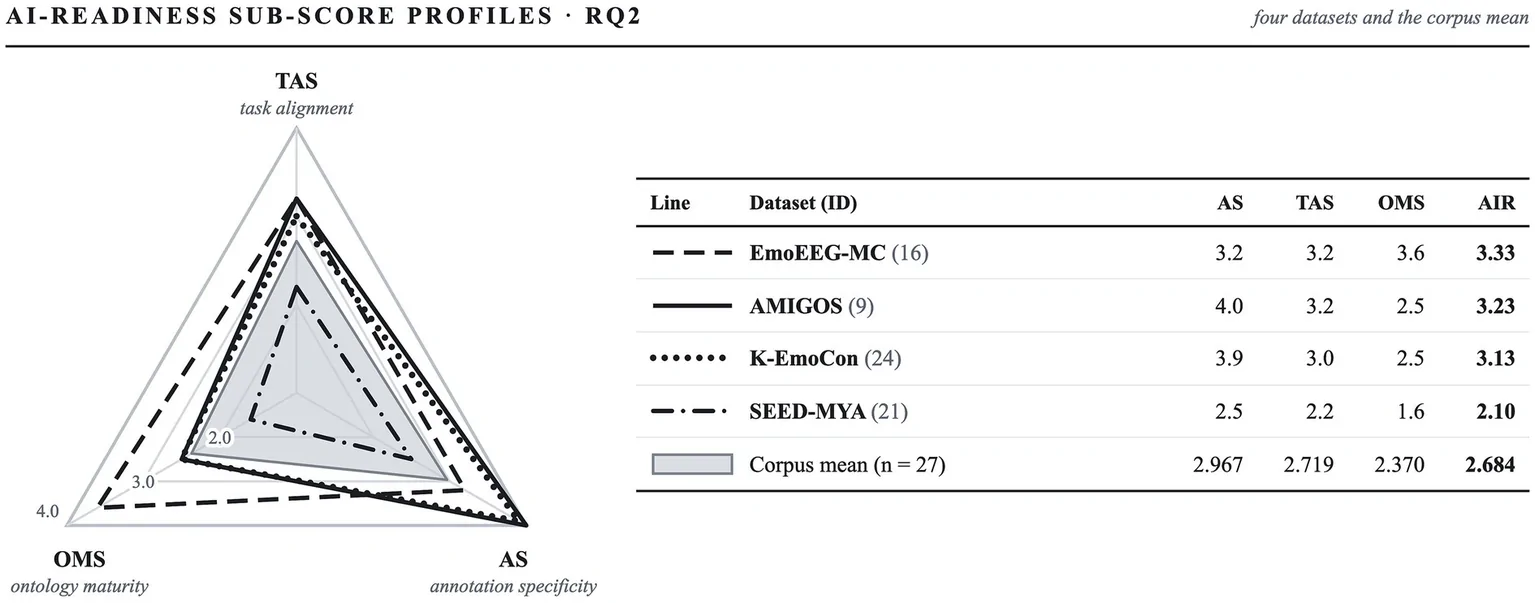
AI-readiness sub-score profiles of four datasets on the three axes (TAS, task alignment; AS, annotation specificity; OMS, ontology maturity), each running from 1.0 at the centre to 4.0 at the outer ring. The profiles are EmoEEG-MC (ID 16, dashed), AMIGOS (9, solid), K-EmoCon (24, dotted) and SEED-MYA (21, dash-dot), shown against the 27-dataset mean (grey area; AS 2.967, TAS 2.719, OMS 2.370). Each profile is drawn with exactly the stroke shown for it in the table, which gives its sub-scores and composite. The strokes are grey, not cluster colours, because EmoEEG-MC and AMIGOS share a task cluster. AMIGOS and K-EmoCon score at or near the maximum on AS (4.0 and 3.9) with mid-scale OMS (2.5), so their lines nearly coincide along the lower edge. EmoEEG-MC has the highest OMS in the corpus (3.6). SEED-MYA, the lowest composite (2.10), lies below the mean on all three axes, and no dataset scores 3.5 or above on all three.

A composite hides which condition a dataset meets, so the sub-scores need to be read (Figure 8). The two highest composites are reached by opposite routes. AMIGOS gets there through annotation: its AS of 4.0, the only one in the corpus, rests on three independent annotators of its released labels, while its structure is mid-scale. EmoEEG-MC gets there through structure: it has the highest OMS, with AS and TAS in the upper range. K-EmoCon follows the AMIGOS pattern. DENS, like EmoEEG-MC, is above the corpus mean on both AS and TAS. SEED-MYA, the lowest composite, is below the mean on all three axes, and no dataset scores 3.5 or above on all three.

Readiness follows label design more than access class (Figure 7). Nine of the 14 datasets at or below the median are discrete-multi-emotion corpora, and of that cluster’s 11 members only SEED-VII and FACED score above the median. Fully open, registration- or licence-gated and conditional datasets, by contrast, spread across most of the range, and their class means differ by less than 0.15. Within the SEED line, readiness follows labels and task support, not hardware. The discrete SEED variants share an identical 62-channel montage, yet seven of the eight sit at or below the corpus median of 2.60. SEED-VII tops the family on the richest labels in it, six basic emotions plus neutral with intensity ratings. SEED-MYA, with flat three-class labels, is the corpus minimum.

Cohort size, recording hardware and sensor breadth do not decide readiness either. The three largest cohorts spread from near the top to near the bottom of the ordering. FACED, the largest, has the highest TAS in the corpus but ranks fifth, because its annotation score is below the corpus mean. LLaMAC sits in the middle, and ECSMP falls into the bottom quarter on thin task support. Consumer headsets also appear across the whole ordering. AMIGOS, second overall, and K-EmoCon, equal third on a single-channel headset, were recorded with consumer devices, as were Emognition and GAMEEMO in the lower half (Table 1 gives cohorts and montages). Datasets that release several physiological streams beside the EEG likewise range from the top, with EmoEEG-MC and AMIGOS, to the bottom quarter, with ECSMP. What the composite rewards is annotation design and task support.

Openness and task support can also come apart. IESD is among the most findable, openly licensed deposits, yet it has the lowest TAS in the corpus. The Daly deposit has strong label structure but weak task support, and its labels are ratings on eight induced-emotion dimensions, including pleasantness and energy, from which valence and arousal must be derived. Voice-UI’s self-reported ratings on nine bipolar adjective pairs place it above the corpus mean on annotation, but the absence of benchmark splits and of any library reader holds its task alignment down.

Agreement between annotators is rarely published. AMIGOS reports it across three annotators, and K-EmoCon reports agreement that its authors describe as low. LLaMAC’s statistic compares intended with reported emotion, a different construct, so it is recorded but not scored.

### 3.4 Ontology maturity (RQ3)

Most datasets have moved past flat labels, but none binds its affect labels to an external vocabulary or ontology. The level distribution is L1 4 (14.8%), L2 7 (25.9%), L3 16 (59.3%), L4 0 (0.0%), L5 0 (0.0%) (Figure 9; level definitions in Table 3). The typical dataset sits at Level 3, the relational or dimensional affect model (for example valence-arousal-dominance). None reaches the formal emotion ontology of Level 4 or the emotion knowledge graph of Level 5.

**Figure 9:**
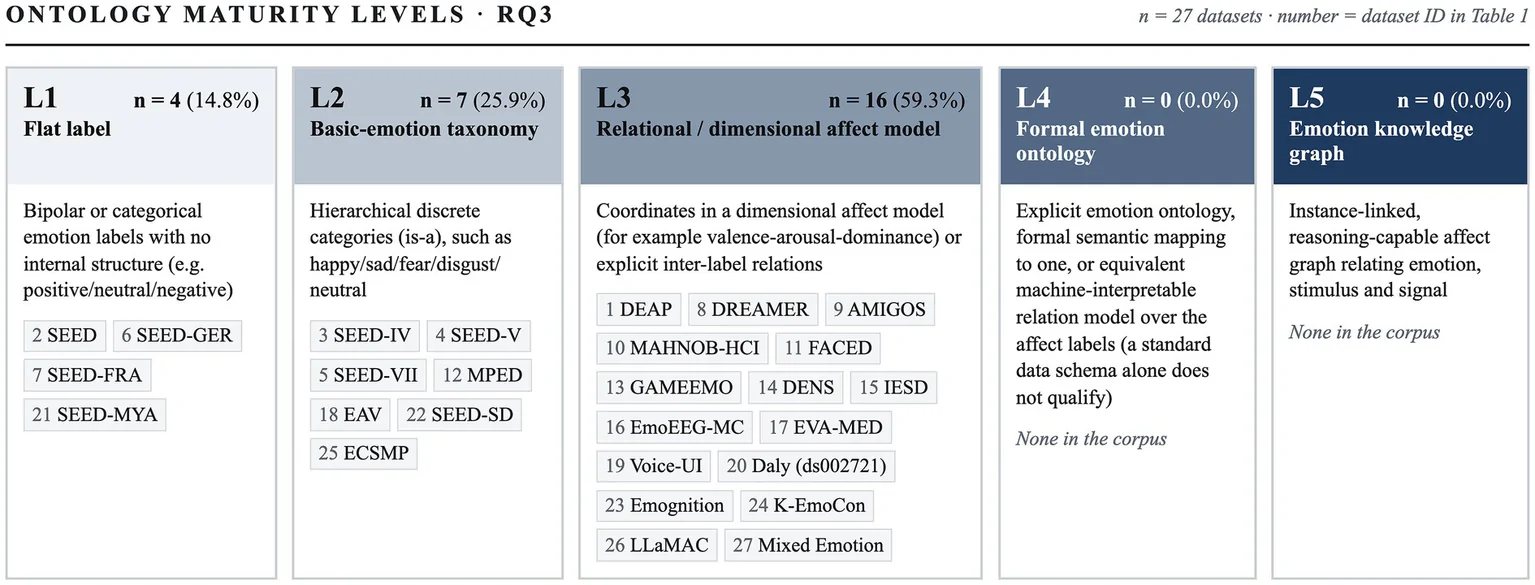
Ontology-maturity levels of the 27 datasets, from L1 (flat label) to L5 (emotion knowledge graph): L1 4 (14.8%), L2 7 (25.9%), L3 16 (59.3%), L4 0 (0.0%), L5 0 (0.0%). Each card gives the level’s operational definition (as in Table 3), its count and share, and its member datasets by Table 1 ID. Level 3 is the relational or dimensional affect model (for example valence-arousal-dominance). The cards are shaded on one light-to-dark ramp, darker for a higher level; the figure uses no task-cluster colour.

Level 3 is not uniform, because its test has three routes (Section 2.4.3). DEAP and DREAMER reach it through released valence, arousal and dominance ratings, and Voice-UI and the Daly deposit through other rated affect dimensions; none of the four releases emotion categories. IESD reaches it only through its 15 imagined-emotion categories and releases no dimensional rating. A reuser who needs valence and arousal labels therefore has to check the route, not only the level (Table 1).

The absence of Level 4 rests on positive evidence, not on a failure to search. The one deposit whose descriptor ships resolvable ontology terms, the Daly deposit, attaches them to the study record (measurement and technology types), not to its affect labels. Its event files contain no ontology terms and none from the Hierarchical Event Descriptors (HED), the Emotion Markup Language (EmotionML) or the Mental Functioning Ontology of Emotions (MFOEM) (Supplementary Section S7.3).

The ceiling on the SEED line is semantic, not instrumental. The four SEED variants with flat three-class labels are at Level 1, and SEED-IV, SEED-V and SEED-SD are at Level 2 with MPED, EAV and ECSMP. SEED-VII is also at Level 2, although it is above the corpus mean on all three AI-readiness sub-scores. Its intensity ratings attach to an assigned category and name no affect dimension, and an intensity attached to a category is not an affect coordinate (Section 2.4.3). Its above-mean OMS reflects montage and schema properties, which OMS credits and the level excludes. No SEED variant reaches Level 3 despite the shared 62-channel montage. The rule that a rating counts only if it is released aligned to the records holds two more datasets at Level 2. EAV and MPED describe valence and arousal ratings in their papers, but neither release was found to distribute them as labels.

EmoEEG-MC comes closest to Level 4. It combines a validated BIDS-EEG schema, rich mixed labels, a peer-reviewed data descriptor with a detailed README and the highest OMS in the corpus. A data schema, however, is a distribution property, not a semantic model, so EmoEEG-MC stays at Level 3. No dataset would reach Level 4 by adding a linking layer alone; each would first need a semantic model of its labels.

On the finer OFI scale, links to standards and external vocabularies separate the top of the ordering from the middle; label richness does not. The corpus OFI mean is 2.385 (Supplementary Figure S2; per-dataset values in Supplementary Table S19). Label structure reaches its observed maximum in nine datasets. Only EmoEEG-MC and the Daly deposit reach the top observed score for links to external standards. The non-BIDS corpora distributed from laboratory pages, DEAP, the SEED line, AMIGOS, MAHNOB-HCI and MPED, hold the 12 lowest scores on those links. The flat-label SEED variants sit lowest on the OFI as a whole, because their label structure and representation formalism are also at the floor. The OFI also separates datasets that share a level: within Level 3 it runs from 2.200 (DREAMER) to 3.433 (EmoEEG-MC), which is why Section 3.5 uses it.

### 3.5 How openness, ontology maturity and readiness relate

Ontology level tracks readiness, partly because the two share inputs. Ontology level correlates with AI-readiness at *ρ* = 0.68, and at *ρ* = 0.40 when OMS is left out of the composite (Supplementary Section S23). Part of the association is therefore shared measurement, because the label structure feeds AS, OMS, the OFI and the level. Even the reduced composite keeps label type through AS and TAS, so this design cannot separate how much of the association is empirical. Figure 10 plots the OFI against readiness, because 16 of the 27 datasets share Level 3 and the level alone would stack them. Access class does not decide the position: the 12 datasets above both means include all three classes. The 10 below both means all release discrete labels only: seven SEED variants, MPED, IESD and EAV. The exceptions show that task support is a separate lever. GAMEEMO, Emognition, Voice-UI and ECSMP combine an above-mean OFI with thin task support, and DREAMER, strong on task support, is the only dataset with above-mean readiness but a below-mean OFI.

**Figure 10:**
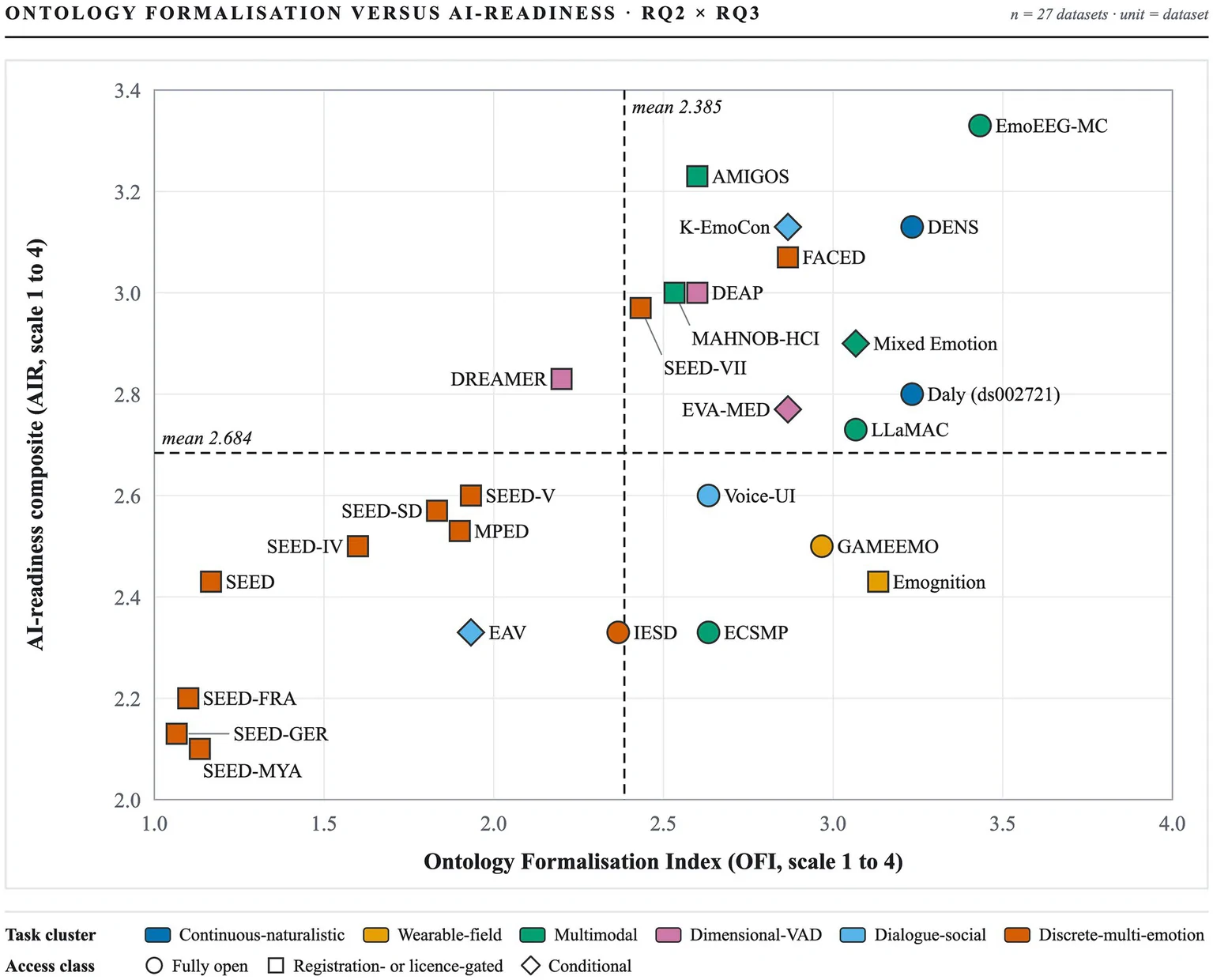
Ontology Formalisation Index (OFI, the mean of three sub-indices for label structure, representation formalism and links to external standards, scale 1.0–4.0) against the AI-readiness composite (AIR, scale 1.0–4.0) for the 27 datasets. Fill shows the task cluster and shape the access class, as in Figure 6; the vertical axis shows the occupied part of its scale (2.0–3.4). Dashed lines mark the corpus means (OFI 2.385; AIR 2.684). SEED-GER, SEED-FRA and SEED-MYA, which share an OFI of 1.100, are drawn side by side (display offset 0.034 on the OFI axis). The OFI is plotted because 16 of the 27 datasets share Level 3, so the level alone would stack them. The correlations reported in Section 3.5 are for the ontology level, not the plotted OFI: level correlates with AI-readiness at *ρ* = 0.68, and at *ρ* = 0.40 when OMS is left out of the composite, partly because the label structure feeds both (Supplementary Section S23). Per-dataset values: Supplementary Table S19 and Figure S2.

Openness does not track readiness. Across the 27 datasets, FAIR openness and AI-readiness are almost unrelated (*ρ* = 0.08), and the association turns negative once ontology level is held constant (partial *ρ* = −0.32; Figure 11). Among datasets at the same ontology level, the more open ones tend to be the less ready. Seven datasets lie below the FAIR mean but above the readiness mean: DEAP, SEED-VII, DREAMER, AMIGOS, MAHNOB-HCI, FACED and EVA-MED. They include all three dimensional-VAD corpora, and six of the seven release valence and arousal self-ratings; SEED-VII is the exception. Three of them, DEAP, AMIGOS and MAHNOB-HCI, are the corpora whose published routes could not be followed at the re-check (Section 3.2). Fully open datasets fall on both sides of the readiness mean, and no registration- or licence-gated corpus is both more open and more ready than the corpus average. These cross-sectional associations do not establish causation, and the small sample limits interpretation of their direction and magnitude.

**Figure 11:**
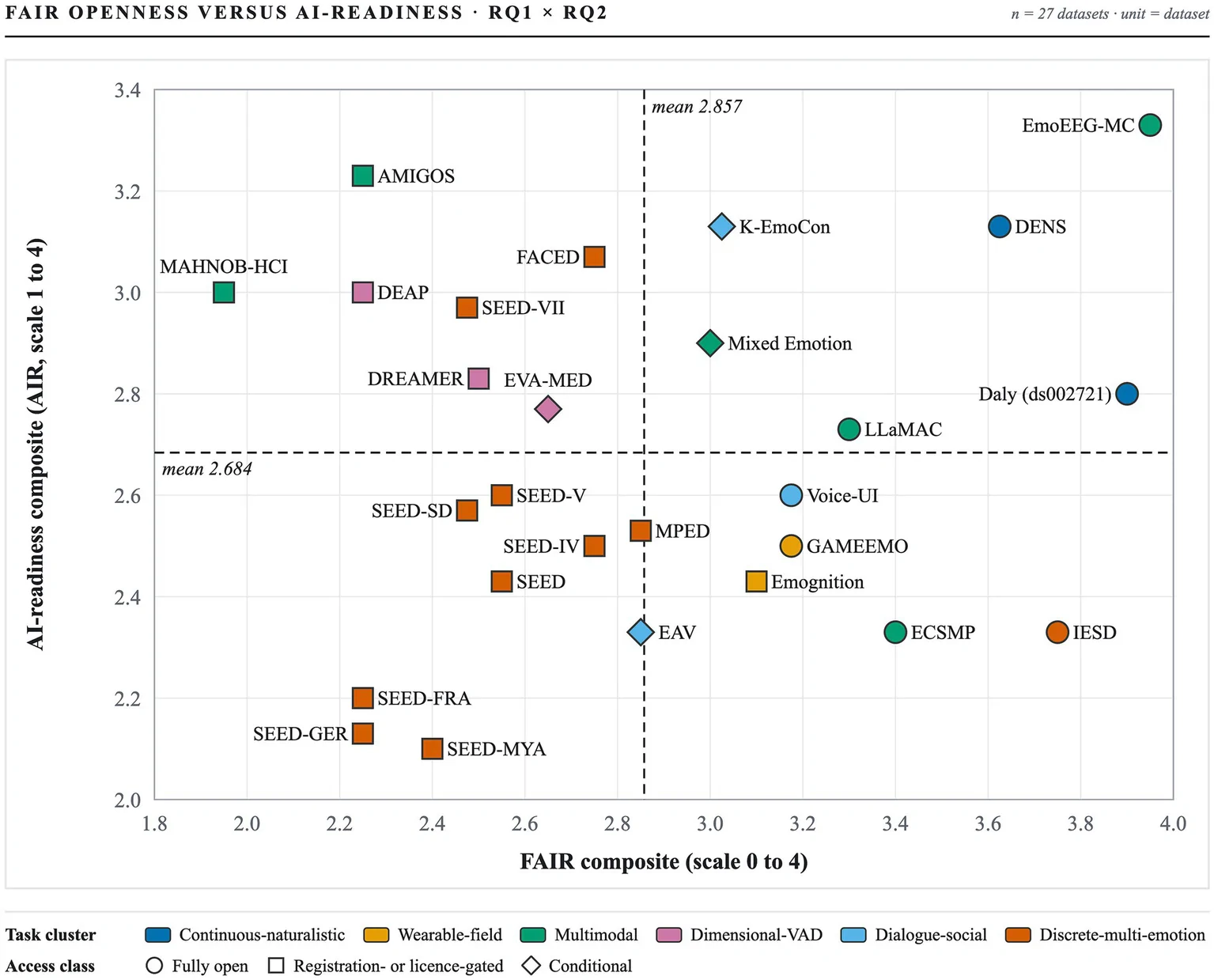
FAIR composite (the mean of F, A, I and R, scale 0.0–4.0) against the AI-readiness composite (AIR, scale 1.0–4.0) for the 27 datasets. Fill shows the task cluster and shape the access class, as in Figure 6; the axes show the occupied part of each scale. Dashed lines mark the corpus means (FAIR 2.857; AI-readiness 2.684). The Spearman correlation between the two composites is *ρ* = 0.08, and partial *ρ* = −0.32 with ontology level held constant (Section 3.5). Fully open datasets span AI-readiness 2.33–3.33, against 2.10–3.33 for all 27. The five spatially situated resources identified in the external coverage check are not plotted because they were not scored on either axis.

Part of the openness-readiness comparison is built into the instruments. BIDS-EEG compliance feeds FAIR Interoperability, OMS and the OFI, and distribution format feeds FAIR Interoperability and the OFI (Supplementary Section S23). When OMS is removed, FAIR correlates with the reduced composite (AS + TAS)/2 at *ρ* = −0.21, although training-framework support still feeds both FAIR Interoperability and TAS. This reduced comparison also provides no evidence of a positive openness-readiness association in the appraised corpus. The near-zero coefficient for the full composite therefore owes part of its value to OMS, whose BIDS-EEG input also feeds FAIR Interoperability. The coefficients in this section describe how the three instruments behave on this corpus; they are not fully independent measurements of three separate properties.

The ordering of datasets does not depend on the equal weights. Each weight in turn was raised or lowered by 0.10, with half of that change taken from or given to each of the other two, giving six alternative schemes. Every scheme gave a Spearman *ρ* of at least 0.982 against the primary ordering, with a worst case of 13 discordant pairs of 351 (96.3% concordance; Supplementary Section S14.1). The largest change comes from down-weighting task alignment.

Corpus composition does not reverse any conclusion either. The eight SEED variants share one laboratory and protocol (Section 3.1). Collapsing them into one averaged unit leaves 20 units and moves mean AI-readiness from 2.684 to 2.77 and the median from 2.60 to 2.785, because the family mean is 2.44. The ontology-readiness correlation falls from *ρ* = 0.68 to *ρ* = 0.56. Sixteen of the 20 units stay at Level 3, none reaches Level 4 or 5, and every association keeps its direction. The 27-dataset enumeration remains the primary analysis, but the effective number of independent units is nearer 20, and the correlations should be read against that count (Supplementary Section S14.3).

## 4 Discussion

### 4.1 What the appraisal shows

The three research questions point to one diagnosis. Emotion EEG has been recorded in quantity, but its metadata, file formats and access terms do not yet let the recordings be pooled for pre-training and transfer. The appraisal also separates three questions that a reuser usually runs together. Can I obtain this dataset, can a model learn from it, and can its labels be mapped onto mine? The answers diverge from dataset to dataset, and that divergence is the main finding. A dataset can be easy to download and weak as a training source, or rich in labels and hard to obtain (Figure 11).

The strongest evidence concerns durability: the eight most-cited appraised corpora all lack a dataset-level identifier. Three of the six most cited, DEAP, AMIGOS and MAHNOB-HCI, could not be reached through their published routes at the re-check (Supplementary Sections S20, S22). A corpus without a dataset-level identifier has delegated its persistence to one web server and to the institution that runs it, so heavy citation confers no durability. The literature built on these corpora now faces an obstacle to new retrieval: its results can still be read, but the data behind them may no longer be obtainable to check or extend them. Citation counts are also negatively associated with openness (Section 3.2), so the datasets the field reuses most tend to be the least FAIR. For anyone who reuses data, the implication is practical: the identifier and the live access route should be checked before a study is built on a dataset (Section 4.2).

Interoperability and semantics are one agenda. Pooling corpora for transfer now needs a bespoke ingestion step for each non-BIDS corpus. Each such step is a place where channel names, event codes or label scales can be misread, and the errors are hard to see once the data are merged. A standard file schema such as BIDS-EEG is also where controlled emotion vocabularies and valence-arousal-dominance coordinates can be encoded so that software can read them. The two problems therefore share one path: a schema first, then a vocabulary bound to it. EmoEEG-MC shows that the schema is within reach for a new release, but no appraised dataset has reached the semantics (Section 3.4). A group planning a new recording can close both gaps at release, which is cheaper than converting a corpus afterwards.

Access terms set the ethical and legal envelope for reuse. The redistribution clauses reported in Section 3.2 mean that a pooled benchmark cannot simply pass on the data of the most-used corpora; every user has to obtain each corpus separately, under its own terms. Emotion is among the most sensitive inferences that can be drawn from neural data. Datasheet-level documentation of consent scope, de-identification and permitted secondary use (Gebru et al., 2021) should therefore be a quality requirement of any release. Only 11 of the 27 corpora are rated Low risk on documentation (risk-of-bias domain D3; Supplementary Section S12). A spatial recording adds position and movement traces, and may add video of the person or the site, which widens what consent and de-identification must cover.

### 4.2 A reusable appraisal method and codebook

The second contribution is a method that other researchers can reuse, and its parts differ in how far they travel. Eligibility, which includes whether a dataset can be obtained at all, is decided before any scoring, so effort goes only to datasets that a reuser could obtain and use. Access is recorded apart from openness, so a reuser sees both what a dataset offers and what obtaining it requires. The FAIR scoring, the access class, the identifier and route audit, and AS transfer as they stand to other uses of labelled EEG. For other data types, the format and loader criteria would need that type’s own standards. The TAS task list and the OMS and ontology-level criteria need adapting: they should name the target field’s own tasks, label vocabulary and sensor conventions. The codebook format makes this adaptation traceable. Each criterion states its evidence, its permitted states and their points, so a new field can replace criteria one at a time and still produce one score from one record. Fields without dedicated datasets, such as neuroarchitecture, need such a method most, because their researchers must borrow data collected for other purposes.

Why should AI-readiness indicate how well a dataset can serve transfer learning? The realistic use of a source corpus is pre-training followed by adaptation with a small target dataset. Adaptation works on what the two datasets share: label dimensions or categories that can be matched, channels that overlap, and data that load into a standard pipeline with splits that allow a fair comparison. AS, TAS and OMS score these properties of the release: labels a model can learn from, fit to standard tasks and tools, and alignment of labels and channels (Table 2). Each can be read from the release itself, before any model is trained. Reported accuracy is not scored, because it depends on the method, the split and the preprocessing, and so describes a study, not a dataset. The published cross-dataset record shows what misalignment costs even between two seated corpora (Section 4.3), which is why alignment is scored and not assumed. The score is therefore a statement about preconditions. A dataset that lacks them needs rework before transfer can be attempted, whereas a dataset that has them can be tried at once. The score does not predict how large the gain from transfer will be.

Is the codebook a sound instrument? Four kinds of evidence bear on this, and each has limits. The first is content: each criterion names a property that a transfer pipeline needs and the recorded field from which it is scored (Supplementary Section S24.2). The second is reproducibility of the rules. Three language-model rater personas reached almost the same criterion states from one shared record (*κ* = 0.947; Section 2.5). This shows that the rules can be applied consistently from one record. Because the three personas share one model (Section 2.6), it does not show that the rules are unambiguous to other coders or that human raters would agree. The third is robustness: the ordering survives other weights, and no conclusion reverses when the SEED line is collapsed into one unit (Section 3.5). The fourth is divergence: readiness is nearly independent of openness (*ρ* = 0.08 with FAIR), so it measures something that openness does not. It also shows no material association with citation counts (Section 3.2), so it does not simply track popularity. That could equally mean that it misses what users value, which only the test proposed below can settle. Some properties are left out on purpose. Signal quality cannot be judged from a release without re-analysis, so only the documentation of acquisition and preprocessing is appraised, in risk-of-bias domain D1. In the readiness score, cohort size counts only up to the 10 participants that a cross-subject split needs; beyond that, AS rewards the number of labelled units each participant contributes. The missing-data rule means that a dataset cannot raise its score by reporting less.

Three things are not yet shown. The first is criterion validity against measured transfer performance. The second is generality: the TAS point shares were set partly by how the benchmark-split criterion discriminated in this corpus, so their fit to other corpora is untested. The third is a spatial-transfer criterion, since TAS does not assess locomotion artefacts, sparse mobile montages or event structures for continuous navigation. A corpus can therefore score well while remaining unsuitable for a particular neuroarchitecture application. The first gap can be tested directly: pre-train models on datasets spread across the readiness range, adapt each to one fixed target dataset, and check whether readiness predicts the gain. We propose this test; we have not run it. If readiness failed to predict the gain, the score would still describe what a release contains, but its claim to index transfer suitability would fall.

The audit also makes the method a test of credibility. Readers cannot check a secondary study whose data cannot be obtained again, however careful its analysis, and in this corpus that risk is concentrated in the most-cited datasets (Section 4.1). Recording whether each dataset can still be found and obtained therefore belongs in any study that reuses data, beside the quality score.

### 4.3 From screen-based to spatial emotion: gaps for neuroarchitecture

The appraised datasets share one paradigm. In the emotion-labelled recordings of all 27 datasets, the participant stays in one place and the emotional material is delivered. Most datasets show films or music videos. Other contexts are music listening (the Daly deposit), computer-game play (GAMEEMO), a screen-based stress test (EVA-MED) and eyes-closed guided imagery (IESD and EmoEEG-MC). Three use conversation, scripted (EAV), unscripted (K-EmoCon) or with a voice interface (Voice-UI). None records EEG while a person moves through a space or chooses what to look at within it. This includes the two wearable-field datasets, recorded with consumer headsets at a screen.

The first gap is self-directed viewing. In a film dataset the experimenter decides what is shown and when, so every label attaches to a known stimulus epoch. In a building the person decides where to look, when and for how long. The eliciting event is then chosen by the participant and must be recovered from gaze and position records time-locked to the EEG, with labels attached to moments the participant selected. The difference is likely to reach the brain response itself, not only how the data are organised. In a film the editor changes the view. In a building each change of view is made by the viewer’s own eyes, head and body, so attention is steered from within. A feeling can then build over many such views instead of following a known cut. A decoder trained on film epochs locked to the stimulus has not seen this regime. Part of the infrastructure exists: nine datasets release eye-tracking data recorded with the EEG (the eight SEED variants and MAHNOB-HCI). All were recorded during experimenter-timed screen viewing, and none records position. Labels that follow the participant have few analogues. In DENS, participants mark when an emotion occurred, and K-EmoCon labels every 5 s of an unscripted conversation from three perspectives. In the imagery designs, participants generate the emotional content themselves, and a button press marks when the feeling begins (IESD) or ends the imagery period (EmoEEG-MC); in GAMEEMO, they act on what they see. None of these involves moving through space, and none defines an epoch by where a person chose to look.

The second gap is movement. MoBI makes it possible to record EEG while people walk (Gramann et al., 2011), and walking changes both the signal and the experience. In the walking study cited in Section 1, posterior cingulate and occipital activity was linked to the changing room perspectives during the walk (Banaei et al., 2017). Those changes of view come from the walker’s own movement, which no seated dataset contains. Walking also adds motion and muscle artefacts and changes posture and head position. A model trained on seated data has seen neither these artefacts nor the brain activity that accompanies active exploration. Four appraised datasets release accelerometer or gyroscope streams (AMIGOS, Emognition, K-EmoCon and ECSMP), but all from stationary sessions. The consumer-headset datasets use sparse montages of the kind worn in mobile recording, yet they too involve no locomotion. Movement could change the EEG-affect relation in two ways: by adding noise that a model must learn to ignore, and by adding brain activity that belongs to the experience itself. The appraised corpora cannot show how large either effect is.

Even the easiest transfer is costly. Cimtay and Ekmekcioglu (2020) report 86.56% cross-subject accuracy within SEED for two emotion classes, but 58.1% when the SEED-trained model is tested on DEAP, close to chance. Lan et al. (2019) compared domain-adaptation methods on the same pair and report gains of 7.25–13.40% over an unadapted baseline, which reduce the penalty but do not remove it. The two corpora share modality, seated posture and screen elicitation. Even so, the comparison required both label sets to be reduced to two classes, because SEED gives flat positive, neutral and negative labels (Level 1) and DEAP gives valence, arousal and dominance ratings (Level 3). By analogy, seated-to-seated transfer is a best case for transfer to walking or immersion, where posture, movement and the eliciting event also differ.

The third gap concerns labels for place. Beyond the valence, arousal and dominance ratings described in Section 1, HERO rates comfort and workload (Supplementary Section S21.1). The stream-sound dataset collects ISO soundscape pleasantness and eight bipolar ratings, and the VR navigation dataset rates anxiety and willingness under approach, interaction and avoidance conditions. The valence and arousal ratings share coordinates with the Level 3 source corpora, so a mapping can be built now. Comfort, workload and approach or avoidance appraise a place or an action within it, and no appraised label set names them. Some words recur, since the Daly deposit rates the pleasantness of music-induced feeling and MAHNOB-HCI offers anxiety as a category. Yet the same word applied to a soundscape or a virtual route need not name the same state. Relating a source label to such a target needs declared relations between terms, which is what Level 4 provides. That is the practical reason ontology maturity is part of the appraisal.

The coverage check found five releases in which the built or spatial environment itself elicits the response. They are HERO (Tomar et al., 2025), a VR urban-square soundscape study (Ming and Zhang, 2026) and a VR navigation dataset (Wu et al., 2026). The other two are an audiovisual VR emotion-induction dataset (Ramirez-Lechuga et al., 2023) and SPACE, whose immersive virtual spaces vary in colour, form and area proportion (Dou, 2026). All five appeared between 2023 and 2026 and share the limits noted in Section 1: each is a single study without benchmark splits, a standard schema or a training harness. Only the VR navigation dataset is described as involving navigation, so movement through space is barely represented even at the frontier. Three are openly released under CC0 or CC-BY; the stream-sound study publishes derived EEG indices with raw EEG on request, and HERO is distributed through an access request. Spatial affective-EEG data therefore exist, but they have not yet entered the shared benchmark tier.

The vetted corpus still offers places to start, although none has been tested for spatial transfer. Sparse consumer or dry-electrode montages close to mobile set-ups are found in DREAMER, AMIGOS, GAMEEMO, Emognition, K-EmoCon, LLaMAC and Mixed Emotion. DENS, IESD and EmoEEG-MC are high-density, fully open OpenNeuro deposits suited to pre-training. The SEED line and MAHNOB-HCI offer gaze-coupled recordings, but all are registration- or licence-gated, and the route to MAHNOB-HCI did not work at the re-check. Released valence and arousal self-ratings of the kind spatial studies collect are found in, for example, DEAP, DREAMER, AMIGOS, DENS, LLaMAC and Mixed Emotion. Not every Level 3 dataset has them, since IESD reaches Level 3 through its number of emotion categories (Table 1; Supplementary Tables S16, S17).

Until spatial datasets exist at benchmark scale, decoders for spatial studies will be pre-trained on seated data and adapted with spatial recordings, a workable but untested bridge. Its weak points are the three gaps above: who chooses the view, whether the body moves, and what the labels mean. The codebook can be extended to these gaps in its own format. New criteria would credit gaze and position time-locked to the EEG, recording during locomotion, events defined by where the participant looked or went, and place-specific labels. On this corpus, such criteria would credit only the screen-based eye-tracking data of nine datasets. The bridge can also be tested now, by adapting a model pre-trained on the high-density open corpora to a spatial release that collects valence and arousal ratings. Section 4.5 sets out what new spatial recordings should contain.

### 4.4 Coverage beyond the appraised corpus

The external coverage check compared the appraised corpus with the authors’ separately compiled landscape of 102 acquisition-level affective-EEG resources. Twenty-six of the 27 appraised corpora were represented in that landscape, through 30 of its units. The absent corpus is K-EmoCon, whose single-electrode NeuroSky MindWave signal was acquired at 125 Hz, while the release contains eight relative band-power series at 32 Hz. It is included here and excluded by the landscape’s distributed-signal criterion. Its scores are retained under this review’s stated rules, while its representation limits must be considered before reuse. Future reviews should explicitly assess the distributed signal representation as well as the acquired modality. Of the 72 landscape units that match no appraised corpus, 69 met this review’s eligibility criteria and 3 failed the access criterion (Supplementary Section S21.2). That criterion was tested on the access class the landscape records. The landscape’s own access audit of 1 August 2026 records 13 of the 69 as lacking a verified route to the EEG, and 2 of the 3 as obtainable.

Retrieval was not exhaustive. Five eligible datasets surfaced only in the second search pass, and seven further second-pass candidates were deferred because the access condition could not be verified (Section 3.1). The second pass used data-paper or dataset document-type limits, which can miss corpora described in conventional articles or conference papers. Without that limit, the recorded IEEE Xplore query returns 2,373 records, including 2,091 dated through 2025, which were not screened (Supplementary Table S1). The authors’ separately compiled 102-unit landscape is not an independent third-party validation source.

The bound still marks a distinct part of the field. The appraised corpora have a median OpenAlex citation count of 94 and a maximum of 5,044, whereas the eligible unmatched resources have a median of 0 and none exceeds one hundred citations (Supplementary Section S21.2). The 27 are therefore the tier that dominates reuse. This difference describes the composition of the selected sample; it does not remove the coverage limitation. Counts and proportions in this article describe the 27 appraised corpora, not the entire affective-EEG landscape. For a reuser, the unappraised eligible resources, which include all five spatially situated releases of Section 4.3, are candidates that the published codebook can score in the same way once their attributes are extracted.

### 4.5 Recommendations for future datasets

New spatial datasets should be born FAIR. They should be released in BIDS-EEG under CC0 or CC-BY, with a persistent dataset-level accession and a datasheet. The target is the profile of EmoEEG-MC and the Daly deposit, although no existing dataset reaches the top Interoperability score. Spatial releases should also record gaze and position time-locked to the EEG and mark the events that participants select, so that labels can follow where people look and move. Participants should rate valence and arousal as well as the place-specific scales, so that new data share coordinates with the Level 3 source corpora.

The second need is a spatial-affect ontology layer. Affect should be annotated with a controlled vocabulary mapped both to valence-arousal-dominance and to architectural concepts such as comfort and approach, stored as machine-readable BIDS event metadata. Existing label sets could be mapped to a shared upper ontology such as MFOEM (Hastings et al., 2011). A “fear” label in one corpus and a high-arousal, negative-valence coordinate in another would then become formally relatable. This is the only route to Level 4 or Level 5.

Transfer also needs scaffolding. New releases should include standard cross-corpus splits and readers for open EEG libraries such as TorchEEG and EEGain. They should also include at least one high-density montage, so that a dense cap can be reduced to a sparse mobile one and the loss measured. The SEED line offers a within-family transfer testbed (Zheng and Lu, 2015), and the high-FAIR OpenNeuro datasets can serve as pre-training anchors.

Table 4 links each recommendation to the result that motivates it. It is meant as a checklist for groups that produce EEG emotion datasets, and in particular for neuroarchitecture groups planning their first spatial release, who can act on it before the first participant is recorded.

**Table 4:** Synthesised recommendations for future EEG emotion-recognition datasets, framed for neuroarchitecture/spatial-emotion reuse.

#	Recommendation	Evidence (this review)	Primary beneficiary
R1	Release in BIDS-EEG with CC0/CC-BY and a persistent accession by default	Interoperability is the weakest FAIR principle (I = 2.044); only the four OpenNeuro corpora (IESD, EmoEEG-MC, Daly, DENS) use BIDS-EEG, and only EmoEEG-MC and Daly validate against it; fully open access reaches only 29.6%, and 13 of 27 corpora (48.1%) carry no dataset-level persistent identifier (Section 3.2)	RQ1 openness
R2	Publish a Gebru-style datasheet documenting consent scope, de-identification, and permissible secondary use	Only 11 of 27 corpora are rated Low risk on documentation/transparency (D3), the weakest risk-of-bias domain after accessibility/reproducibility (D4, 5 of 27); ethical sensitivity of neural affect data (Section 4.1)	RQ1/ethics
R3	Annotate with a controlled emotion vocabulary mapped to V-A-D and to an architectural-emotion concept set	16/27 (59.3%) stall at L3; none reaches L4 or L5; links to external standards form the weakest OFI sub-index	RQ3 ontology
R4	Provide multi-rater annotation, continuous-intensity labels, and standard cross-subject splits	The corpora at the head of the ordering (EmoEEG-MC, AMIGOS, DENS, K-EmoCon) all score above the corpus mean on both AS (3.0–4.0) and TAS (3.0–3.2)	RQ2 AI-readiness
R5	Record EEG with time-locked gaze and position during VR architectural walkthroughs and on-site MoBI walking, and mark self-selected events	Field-application gap: 0 of the 27 appraised corpora use spatial or immersive locomotion paradigms (Section 4.3), and the five spatially situated affect-EEG resources identified in the external coverage check (Section 4.4) all sit outside the benchmark tier	Neuroarchitecture
R6	Include a high-density montage and concurrent multimodal channels (eye/IMU/ECG) for embodied-affect fusion	14 of the 27 record 62 or more EEG channels; nine release eye-tracking data, all during screen viewing; four release accelerometry, all from stationary sessions; none records position (Table 1; Supplementary Table S16)	Multimodal/VR-MoBI
R7	Publish standard transfer splits and a training-framework reader of the TorchEEG or EEGain kind to enable pre-train-on-seated/fine-tune-on-spatial reuse	Strong open seated-paradigm anchors exist but no spatial data in the benchmark tier; transfer is the only near-term route (Sections 4.3 and 4.5)	Cross-corpus reuse

### 4.6 Limitations

Scores are determined by the published codebook and the source-verified attribute record (Supplementary Section S24). They describe documented resource properties at the recorded check dates. Repository contents, licence statements and access routes can change, so the appraisal is time-specific. The Year field is the descriptor-record publication year; recording both original-study and public-release years would better support analyses of infrastructure adoption. The criteria are ordinal, so small differences in score are not practical differences.

AI-readiness is an inferred composite, not a measure of model accuracy or of validated spatial-transfer performance. Its stability under the tested weights does not validate the criteria or set thresholds for any application, and the TAS point shares, set on this corpus, may not generalise (Section 4.2).

The reproducibility test used three language-model rater personas (Section 2.6). They share one underlying language model, so their errors are not independent in the way assumed by Fleiss’ *κ*. They also use the same extracted record, meaning that an upstream error about a licence, label model, access route or montage can be repeated by all three. Excluding unanimous items, the mean coefficient was 0.895. This second summary shows how unanimity affects the overall mean; it is not a statistical lower confidence bound. Four invariant items have undefined *κ*. Human authors adjudicated the extracted attributes against the cited sources, but independent dual-human screening, a human consensus exercise and a human inter-rater coefficient were not obtained. Automated agreement does not establish human-equivalent reliability.

The sample is small (*N* = 27), and the eight related SEED variants reduce the effective independence of observations (Section 3.5). Several scores also share inputs, so part of each association is shared measurement. These estimates describe this corpus and should not be interpreted causally. The six task clusters are descriptive labels recorded once in the corpus record, and several datasets share features with another cluster (Table 1); they enter no score, and no conclusion here depends on them.

The risk-of-bias audit points the same way as the FAIR results. Low ratings number 18, 20, 11 and 5 across domains D1–D4. The audit is strongest at annotation and dataset construction and weakest at accessibility, where the single High rating falls (MPED, D4; Supplementary Section S12). This convergence is not independent corroboration in the measurement sense: the adapted D3 and D4 domains draw on the same evidence about persistent identifiers, formats, documentation, licensing and retrievability that the FAIR score uses.

The absence of closed datasets follows from the eligibility rule (Section 2.3).

## 5 Conclusion

Neuroarchitecture has no dedicated EEG emotion datasets in the shared benchmark tier, so its computational models must start from general emotion datasets recorded from seated participants. This review asked which of those datasets can serve as transfer sources. Its first contribution is a vetted corpus: 27 persistently documented datasets, none designed for spatial research, identified and reported under PRISMA 2020 and each appraised with the published codebook, with starting points for spatial studies (Section 4.3). Its second contribution is a reusable method. It combines PICOD scoping, a public-obtainability rule for eligibility, three instruments driven by one published codebook with scripts, and audits of identifiers, access routes and risk of bias (recommendations in Table 4). Three language-model rater personas (one model; Section 2.6) reproduced the coding, with a mean item-level Fleiss’ *κ* of 0.947 over the 49 items with defined coefficients. This value describes how reproducible the codebook is given one shared record, not agreement between human raters.

For a researcher choosing a source dataset, three results matter most. First, a dataset that can be downloaded cannot necessarily be combined with others: Interoperability averaged 2.044 against 3.511 for Accessibility, and only two releases validate against BIDS-EEG. Second, heavy use does not keep a dataset obtainable. A dataset-level identifier is missing for 13 of the 27 datasets, and the published routes of three heavily cited corpora did not work at the re-check. Third, openness says little about readiness (*ρ* = 0.08), whereas ontology level tracks it (*ρ* = 0.68), in part through shared inputs. Openness and readiness therefore have to be judged separately, and whether a dataset can still be obtained has to be checked before a study is built on it. No dataset reaches ontology Level 4, so no current label set can yet be related formally to the comfort or approach judgements that spatial studies collect.

The conclusions apply to a bounded sample of 27 corpora. Five eligible resources surfaced only in the second search pass, and further eligible resources lie outside the appraisal, so retrieval was not exhaustive. The correlations rest on a small sample with related SEED variants. The ordering is stable within the tested weight perturbations, but the scores are not validated measures of neuroarchitecture transfer performance. The reproducibility package allows every reported score to be recomputed. The larger gap lies outside the corpus. None of the shared datasets records EEG while people move through designed space, and the five spatial releases found by our coverage check, all from 2023 to 2026, remain single studies outside the benchmark tier. The next step is spatial datasets released with persistent identifiers, open licences and a standard format, with labels linked to an emotion ontology, and with records of where people look and move. Such datasets would let computational models of how people respond to designed space be trained and tested in the settings they are meant to serve.

## Data Availability

The original contributions presented in the study are included in the article/Supplementary material, further inquiries can be directed to the corresponding author/s.
