# Supplementary material for: EEG emotion-recognition datasets for neuroarchitecture: an appraisal of openness, AI-readiness and ontology maturity
Source: Front Neuroinform. 2026 Oct 20;20:1971441. doi: 10.3389/fninf.2026.1971441 (PMC13727589; doi:10.3389/fninf.2026.1971441)
Supplement: Supplementary file 2 [file Supplementary_file_1.docx]

Supplementary Material

# Supplementary Material, Sections S0–S10

Systematic review of EEG-based emotion-recognition datasets for neuroarchitecture / spatial-emotion research (PRISMA 2020). Unit of analysis = dataset (not paper). The corpus comprises N = 27 included datasets and 11 excluded or held resources, together with one first-pass screening exclusion carried under EX-0 by reason and stage alone; the dispositions of the 32 records returned by the second search pass are given in Section S4b. All numbers in this supplement match the canonical corpus and the main-text statistics exactly. Excluded datasets are never scored, listed among the appraised corpora, or entered into any level count or cluster mean.

This supplement comprises Sections S0 to S25 in four parts. Sections S0 to S10, together with S3b and S4b, document the review protocol as executed: the inclusion criteria, the Stream A and Stream B searches, the two-stream PRISMA 2020 summary (S4) followed by the resource-by-resource Stream A reconciliation ledger (S4b), the per-dataset provenance, the exclusion log, the datasheets, the PRISMA 2020 checklist mapping and the PICOD record. Sections S11 to S19 report the appraisal: the codebook-reproducibility assessment by three language-model rater personas (S11), the risk of bias, the eligibility assessment, the AI-readiness weighting, the AI-readiness scorecard, a structured overview of all 27 datasets and the per-dataset FAIR, AI-readiness and ontology-formalisation scores. Sections S20 and S21 report the persistent-identifier resolution ledger and the external coverage reconciliation, and Sections S22 to S24 report the adoption ledger, the construct-overlap matrix and the deterministic scoring codebook. Section S25 holds Supplementary Figures S1 and S2, which show the FAIR principle scores and the ontology-formalisation sub-indices of the 27 datasets. The accompanying transparency workbook tabulates the headline statistics, the Stream A lane counts, the identification funnel, the raw reliability codings and their coefficients, the risk-of-bias matrix, the per-dataset scores, the identifier ledger and the adoption ledger. The machine-readable inputs and the nine scripts that recompute the reported scores and summary statistics from them are distributed as the reproducibility package (Reproducibility_package.zip), and files in it are cited in this supplement by their package path, for example data/corpus.json or scripts/verify_docs.py.

## S0 Key Inclusion Criteria

(Summary of manuscript Section 2.3; the exclusion codes defined below are those cited in manuscript Sections 2.2 and 3.1.)

A resource is included if and only if it satisfies, uniformly, both of the following criteria, after first passing the EEG-specific eligibility gate.

EEG eligibility gate (necessary precondition, applied first). The resource must contain scalp EEG signal AND emotion/affect labels, discrete emotion categories (e.g. happy / sad / fear / neutral / disgust) or dimensional affect ratings (for example valence, arousal and dominance, abbreviated V-A-D). A resource lacking scalp EEG, or carrying affect labels only on a non-EEG modality, or carrying no affect label, fails the gate and is logged in the exclusion record (Section S6) with its EX-code; it is never scored.

| **Criterion** | **Statement** | **Operationalisation** |
| --- | --- | --- |
| Publicly obtainable | A dataset has a persistent public record and can be obtained by eligible researchers worldwide under stated terms. This does not necessarily mean unrestricted download, redistribution or commercial reuse. | Satisfied by either of two routes, which together form a single rule: a verified dataset-level persistent identifier (DOI, OpenNeuro accession, Zenodo / Mendeley Data / figshare / Dataverse / Synapse record), or, where no such identifier exists, a documented request procedure open to applicants of any nationality or region. Fully open, registration-gated, EULA and DUA routes are all eligible, as are free accounts, purchasable subscriptions and published discretionary approval steps; only non-universality or absence of a route fails. The condition is evaluated at a dated check and carries a time dimension: a resource whose route was functional when the corpus was assembled but did not respond at the re-check of 2026-09-11 is retained in the corpus and flagged on the access_status axis rather than excluded, because excluding it would delete the very finding that the route has failed (Sections S17 and S20). |
| Universally grantable access | The access route can in principle be granted to any qualified researcher irrespective of nationality or region. Nationality- or geography-gated routes are not eligible. | Applied only after the EEG precondition (scalp EEG signal AND emotion/affect label). Access class is then recorded on a separate axis (S17) and contributes no points to the FAIR score. The absence of closed datasets from the corpus is a property of this sampling frame, not a finding about the field. |

No fixed sample-size floor is imposed: EEG corpora are intrinsically smaller than image datasets, so subject count and trial count are scored under the AI-readiness Annotation (AS) and Task-Alignment (TAS) axes rather than used as a cut-off.

The exclusion-reason taxonomy applied during screening and full-text / full-census verification is set out below: one screening-stage code (EX-0) and four full-resource codes (EX-1 to EX-4).
Of the six first-pass screening exclusions in the Stream A census, five were examined again when the full resource was opened and carry EX-1 or EX-2; the sixth was removed at title and abstract as non-emotion on the bibliographic record alone, before the resource itself was opened, and is coded EX-0 (Sections S4, S4b and S6). The six records removed at screening in the second search pass are not dataset descriptors and are also coded EX-0 (Section S4b).

EX-0: Screening-stage exclusion (removed at title/abstract or landing-page screening, before the resource was opened, because the face of the record shows that it does not describe an EEG emotion dataset: it carries no affective content, or it is not a dataset descriptor, for example a methods or secondary-analysis paper that releases no new dataset). EX-1 to EX-4 below all presuppose that the resource was opened, so none of them can carry a screening judgement.

EX-1: No EEG signal (peripheral physiology / behaviour only).

EX-2: Non-EEG neuroimaging (affect label carried by a non-EEG modality, e.g. MEG, fMRI); at the second search pass the code was also applied to non-scalp neural recordings such as intracranial EEG (Section S4b).

EX-3: No emotion label (motor-imagery / BCI / vigilance / sleep / visual-perception / cognitive-workload only).

EX-4: No universally completable access route: neither a dataset-level persistent identifier resolving to the data nor a request procedure that any researcher can complete irrespective of nationality or region. A free account, a signed licence, a purchasable subscription or a disclosed approval step does not trigger this code.

## S1 Stream A: Databases & Registers, Queries and Yields

(Supports manuscript Section 2.2.)

Stream A is the academic-database / register identification stream. Each database was queried with a domain-specific Boolean string combining an EEG facet, an emotion/affect facet, and a dataset/database facet, with no language restriction. The strings were run in two search passes: the first search pass (pass 1) on 2026-06-17, recorded only as aggregate counts, and the second search pass (pass 2) from 10 to 12 September 2026, recorded at record level (Section S4b). The pass 1 counts in the fourth column are distinct verified data resources identified in each lane after de-duplication within Stream A and before title-and-abstract screening, not raw hit counts and not citation-edge metacounts (the count is reported at the stage at which it was recorded). The per-lane counts are not additive (they sum to 40); Section S4 places the merged Stream A identification total at the same pre-screening stage. The three right-hand columns report pass 2: the field searched with the database-level limit, the number of records the string returns without that limit, and the number exported under the limit. IEEE Xplore filters by year only, so its unrestricted count is given in full and to the end of 2025; PubMed is cut at the Entrez date of 17 June 2026 and Semantic Scholar at the publication date of 17 June 2026; Nature Scientific Data gives the journal’s own full-text search count, with the title-and-abstract count to 17 June 2026 in brackets. Semantic Scholar was queried with the two strings shown, and its two counts are given in the same order. The unrestricted totals for Scopus and Web of Science were not recorded at pass 2.

| **#** | **Database / Register** | **Query string (facet-combined)** | **Distinct resources identified, first search pass (17 June 2026)** | **Field searched; limit applied at the second search pass** | **Records without limit (second search pass, 12 September 2026)** | **Exported under limit (second search pass, 10 September 2026)** |
| --- | --- | --- | --- | --- | --- | --- |
| 1 | IEEE Xplore | ("EEG" OR "electroencephalogra*") AND ("emotion*" OR "affect*" OR "valence" OR "arousal") AND ("dataset" OR "database" OR "corpus" OR "benchmark") | 11 | All Metadata; Supplemental Items = Datasets | 2,373 (2,091 to end-2025) | 9 |
| 2 | Scopus | TITLE-ABS-KEY(("EEG" OR "electroencephalogram") AND ("emotion recognition" OR "affective") AND ("dataset" OR "database")) | 8 | TITLE-ABS-KEY; document type = Data paper | Not recorded | 14 |
| 3 | Web of Science | TS=(("EEG") AND ("emotion*" OR "affect*") AND ("dataset" OR "database" OR "corpus")) | 6 | Topic (TS); document type = Data Paper | Not recorded | 15 |
| 4 | PubMed / MEDLINE | (electroencephalography[MeSH] OR EEG[tiab]) AND (emotion*[tiab] OR affect*[tiab]) AND (dataset[tiab] OR database[tiab]) | 4 | MeSH and title/abstract; no limit | 1,199 (Entrez date to 17 June 2026) | Not exported |
| 5 | Nature Scientific Data | EEG AND (emotion OR affect OR "valence-arousal") (data-descriptor venue) | 6 | Journal full-text search; no limit | 145 (title and abstract to 17 June 2026: 20) | Not exported |
| 6 | Semantic Scholar (register search) | EEG emotion dataset / EEG affective database | 5 | Bulk search, terms joined by AND, published to 17 June 2026; no limit | 2,680 / 500 | Not exported |

The six database lanes were merged and de-duplicated. The screening and verification figures recorded in the first-pass census log are gate-passage events, not distinct resources: 6 exclusions at title/abstract screening on the EEG-gate / emotion-label criteria and 7 at full-text / full-census verification, with five resources (CASE, WESAD, VREED, MVRS, DECAF) appearing in both records. On a distinct-resource basis 8 resources left Stream A, 6 at screening and 2 at verification (SEED-VIG, SEED-DV). Section S4b reconciles the two bases entry by entry, and manuscript Figure 3 shows both. Seed/anchor corpora (DEAP, the SEED family, AMIGOS, DREAMER, FACED) surfaced in both streams and are attributed to Stream A to prevent double-counting. At the second search pass, run on 2026-09-10 under the document-type limits recorded in the table, the strings tabulated above returned five further eligible resources that the first pass did not enumerate: Emognition (Saganowski et al., 2022), K-EmoCon (Park et al., 2020) and ECSMP (Gao et al., 2021) in the Scopus and Web of Science lanes, and LLaMAC (Lee and Kim, 2025) and the Tsinghua Mixed Emotion corpus (Yang et al., 2024) in the Scopus lane. All five carry a dataset-level persistent identifier and all five are admitted to the corpus.

The second search pass and what it returned. The Stream A strings recorded above were run a second time on 2026-09-10 in the IEEE Xplore, Scopus and Web of Science lanes, each under a database-level document-type limit (IEEE Xplore, Supplemental Items restricted to Datasets; Scopus, document type Data paper; Web of Science, document type Data Paper). Every record returned under the limit was exported, so no hand selection intervened between search and export. Within those exports, the Scopus and Web of Science strings return five eligible resources that the first pass did not enumerate: Emognition (Saganowski et al., 2022), K-EmoCon (Park et al., 2020), ECSMP (Gao et al., 2021), LLaMAC (Lee and Kim, 2025) and Mixed Emotion (Tsinghua; Yang et al., 2024). Each satisfies criteria (a) to (e) of Section S13, and each carries a dataset-level persistent identifier, resolved when criterion (d) was evaluated on 2026-09-11 and checked in the identifier ledger of Section S20 on 2026-09-12: Emognition 10.7910/DVN/R9WAF4 (Harvard Dataverse), K-EmoCon 10.5281/zenodo.3931963, ECSMP 10.17632/vn5nknh3mn (Mendeley Data), LLaMAC 10.6084/m9.figshare.28748696 and Mixed Emotion 10.5281/zenodo.11194571. Seven further candidates in the exports pass the EEG and emotion-label gates but were deferred rather than appraised, because criterion (d), condition (3) of manuscript Section 2.3, was not verified for them; they are listed in Section S4b and in data/S4b_pass2_records.csv. The five admitted corpora are not a routine addition. Their omission is a limitation of the first-pass retrieval and not a boundary of the search design, because the design as documented in this section is what returned them; the strings were adequate and their execution was not. Two of the five, K-EmoCon and Emognition, are moreover recorded in the working notes of the first-pass census, Emognition with an explicit include decision, so for those two the loss occurred after the eligibility judgement had been made rather than before it. The appraised corpus is accordingly 27, and Sections S4, S5, S13, S16 to S19 and S21 are reported on that basis. The residual uncertainty concerns the first pass: neither its per-lane hit totals nor the limits applied in each lane were recorded, so this review cannot state how many records each lane returned at that time, only that the recorded strings return these five. The second pass therefore records, in the table above, the field searched, the limit applied, the number of records each string returns without the limit where that total was recorded, and the number exported under the limit. The same record settles the stream attribution of the five: each is returned by at least one Stream A lane, and the recorded PubMed string returns all five without any limit (Section S5).

## S2 Stream B: Website / Data-Portal & Repository Search Log (PRISMA-S Item 4)

(Supports manuscript Section 2.2. PRISMA-S Item 4, “other resources searched”.)

Stream B browsed data portals, registries and repositories for standalone EEG emotion corpora, including those released without a conventional seed paper. Each portal was browsed with its native filters (modality = EEG, topic = emotion/affect) on 2026-06-17. “Resources screened” = candidate records inspected; “net-new included” = resources added to the corpus after de-duplication against the Stream A seed/anchor corpora.

| **#** | **Portal / Repository** | **Native filter / query** | **Resources screened** | **Net-new included (Stream B portal)** |
| --- | --- | --- | --- | --- |
| 1 | OpenNeuro / NEMAR | modality:EEG + keyword emotion / affect | 9 | 3 |
| 2 | Zenodo | EEG emotion dataset | 6 | 0 |
| 3 | Mendeley Data | EEG emotion | 3 | 0 |
| 4 | IEEE DataPort | EEG emotion | 3 | 0 |
| 5 | Kaggle | EEG emotion recognition | 4 | 0 |
| 6 | Hugging Face Datasets | EEG emotion | 2 | 0 |
| 7 | Papers With Code | EEG emotion recognition benchmark | 4 | 0 |
| 8 | GitHub (dataset repos) | EEG emotion dataset | 5 | 0 |

Stream B data-portal browsing contributed 3 net-new included datasets, all from the OpenNeuro/NEMAR lane: IESD (ds003004), EmoEEG-MC (ds005540), and the OpenNeuro Film-Clips / Daly Affective-Music family (ds002721 / ds002722). Candidates surfaced on other portals were duplicates of Stream A seed corpora (e.g. DEAP, SEED, DREAMER mirrors), were excluded on the EEG/emotion gate, or could not be verified as universally accessible (see Section S6, EX-4 / held-back entry EEGEMO).

## S3 Stream B: Citation Searching Yields (Observed)

(Supports manuscript Section 2.2.)

Caveat on what the citation counts mean. The raw forward/backward totals returned by the Semantic Scholar Graph API are citation EDGE counts, not retrieved records. A single dataset can be reached by hundreds or thousands of citation edges. The citation-edge column below therefore reflects API metacounts, each the sum of the five per-seed counts retrieved on 2026-09-25 and listed in Section S3b (edge counts at the time of the first search pass were not recorded); only hand-verified, existing, universally accessible resources are counted as included, and these are de-duplicated against the Stream A seed/anchor corpora. We do not reconstruct an unreproducible de-duplication log or present edge counts as a record yield.

| **Search direction** | **Seeds used** | **Citation edges (API metacount, retrieved 2026-09-25)** | **Distinct candidate datasets surfaced** | **Net-new verified included** |
| --- | --- | --- | --- | --- |
| Forward (citing) | 5 anchors | 8,932 | 9 | 3 |
| Backward (cited) | 5 anchors | 278 | 5 | 0 |

Stream B citation searching contributed 3 net-new included datasets: MPED (forward from SEED/DEAP citation network + GitHub portal verification), GAMEEMO (forward + Mendeley Data verification), and EAV (forward + Zenodo verification). All five anchor corpora reached by citation searching (DEAP, SEED family, AMIGOS, DREAMER, FACED) are attributed to Stream A, not re-counted here.

## S3b Seed Papers and Citation Yields (Semantic Scholar Graph API, dated)

(Supports manuscript Section 2.2.)

Citation searching was anchored on five seed publications, queried via the Semantic Scholar Graph API on 2026-06-17. Forward = papers citing the seed; backward = references of the seed. Edge counts at the time of the first search pass were not recorded; the counts in the table below were retrieved from the same API on 2026-09-25 by the DOI of each seed (forward = citationCount, backward = referenceCount) and are distributed as data/S3b_citation_counts.csv in the reproducibility package (Reproducibility_package.zip). The forward and backward numbers are citation EDGE counts (API metacounts), not retrieved or de-duplicated records. They are reported for transparency of effort, not as an inclusion yield; because they describe the seed papers rather than the records screened, the later retrieval date leaves the citation-searching yield unchanged at three net-new datasets (MPED, GAMEEMO and EAV).

| **Seed (anchor) corpus** | **Seed primary reference** | **Semantic Scholar forward edges (citationCount, 2026-09-25)** | **Semantic Scholar backward edges (referenceCount, 2026-09-25)** | **Net-new datasets verified from this seed** |
| --- | --- | --- | --- | --- |
| DEAP | Koelstra et al., 2012 (IEEE T-AFFC) | 4,723 | 60 | MPED, GAMEEMO (forward) |
| SEED | Zheng & Lu, 2015 (IEEE TAMD) | 2,401 | 61 | MPED (forward) |
| AMIGOS | Miranda-Correa et al., 2018 (IEEE TAFFC) | 635 | 74 | n/a (anchors only) |
| DREAMER | Katsigiannis & Ramzan, 2018 (IEEE JBHI) | 1,024 | 44 | n/a (anchors only) |
| FACED | Chen et al., 2023 (Nature Sci. Data) | 149 | 39 | EAV (forward) |

Edge totals are API metacounts, retrieved on 2026-09-25, that fluctuate with index updates; they indicate the scale of the citation network around each seed but are not summed into any record-level funnel number. The de-duplicated, hand-verified net-new yield of citation searching is 3 datasets (MPED, GAMEEMO, EAV), consistent with Sections S3, S4 and S8.

## S4 Two-Stream PRISMA 2020 Summary

(Supports manuscript Section 3.1.)

The identification design is two-stream and additive. Unit of analysis = dataset. The inclusion counts are distinct verified resources and the identification count is the first-pass aggregate as recorded (Section S4b.1), and none of them is a citation-edge metacount; the two exclusion counts are gate-passage events, and the difference between the two units is stated below the table rather than smoothed away.

The Stream A column is the funnel of the first search pass, with the two SEED-line variants verified and added after it. The five resources identified at the second search pass are added below the table, and Section S4b, which follows, sets the two passes side by side with the resource-by-resource Stream A ledger.

| **Stage** | **Stream A (Databases & Registers)** | **Stream B (Other methods)** |
| --- | --- | --- |
| Records / resources identified | 29 | citation searching + portal browsing (see S3, S3b, S2) |
| De-duplicated | 29 | n/a |
| Screened (title/abstract) | 29 | n/a |
| Excluded at screening | 6 | n/a |
| Sought for full-text / full-census verification | 23 | n/a |
| Excluded at full text | 7 | n/a |
| Passing verification | 16 | n/a |
| Excluded for accessibility | 0 | n/a |
| Included | 16 | 6 (3 citation + 3 portal) |

Total included corpus = 27 = Stream A 16 + Stream B citation 3 + Stream B portal 3 + 5 identified at the second search pass of the Stream A strings (2026-09-10). Eleven named resources are excluded or held (Section S6), together with one unnamed screening exclusion coded EX-0.
Three features of this funnel are disclosed rather than smoothed. First, the identification total of 29 is the first-pass census total of 27 resources, recorded in aggregate, plus the two SEED-line variants, SEED-MYA and SEED-SD, verified and added after the first pass; the funnel therefore reconciles only when it is read as it was recorded, and this review prints the recorded lineage instead of a reconstructed one. Second, the screening and full-resource exclusion counts, 6 and 7, are gate-passage events rather than distinct resources: five resources, CASE, WESAD, VREED, MVRS and DECAF, were excluded at title and abstract and then re-confirmed when the full resource was opened, so each is recorded at both gates. On a distinct-resource basis 8 resources departed Stream A, of which 7 are named: CASE, WESAD, VREED, MVRS, DECAF, SEED-VIG and SEED-DV. The eighth was excluded at title and abstract as non-emotion and its name was not retained in the census log; it is coded EX-0 and reported as the single residual gap in the disposition record, recoverable only by re-running the lane. Third, the Stream A full-resource exclusions are CASE, WESAD, VREED and MVRS (EX-1, no EEG), DECAF (EX-2, non-EEG neuroimaging), and SEED-VIG and SEED-DV (EX-3, no emotion label). NeuroEmo, SEED-VLA/VRW and EEGEMO, although all three appear in the exclusion log, never occupied a Stream A funnel cell: they were reached through Stream B portals and are recorded as such in Section S6. Section S6 therefore logs a different population from this funnel, which is the whole of the arithmetic difficulty, and each logged resource is counted exactly once within its own population.

## S4b Stream A Resource Reconciliation Ledger

This section reconciles, resource by resource, the Stream A counts summarised in Section S4 and reported in manuscript Sections 2.2 and 3.1 and in Figure 3. It reports the identification as two passes, because only the second is complete at record level: S4b.1 reconciles the first-pass gate events with distinct resources, S4b.2 sets the two passes side by side, S4b.3 gives the disposition of every Stream A resource and of every entry of the exclusion log, and S4b.4 defines the screening code EX-0.

### S4b.1 Gate-passage events and distinct resources

The pass-1 screening and full-resource exclusion counts (6 and 7; manuscript Figure 3 and Section S4) are gate-passage events, not distinct resources. Five resources are recorded at both gates: CASE, WESAD, VREED, MVRS and DECAF were excluded at title and abstract screening and recorded again when the resource itself was examined. The thirteen departures are therefore eight distinct resources and five repeats.

The eleven-item exclusion log then reconciles exactly: seven named Stream A departures, three resources reached through Stream B portals (NeuroEmo through OpenNeuro, EEGEMO through Zenodo, SEED-VLA/VRW through the BCMI portal) and one label for which no distinct corpus could be verified (SEED-Multimodal). Seven plus three plus one is eleven.

A residual remains and is disclosed rather than reconstructed. Twenty-seven identified minus eight distinct departures is nineteen, which does not reconcile with the fourteen Stream A inclusions recorded at the time (the sixteen of Section S4 less SEED-MYA and SEED-SD, which were added after the first pass). The census log accounts for twenty-two resources of the Stream A total: the fourteen inclusions and the eight distinct departures, one of them unnamed. We judge that the figure of twenty-seven was an aggregate estimate rather than a record-level count, and we do not reconstruct a ledger we cannot evidence.

### S4b.2 Two-pass identification

| **Stage** | **Pass 1 (2026-06-17)** | **Pass 2 (2026-09-10)** |
| --- | --- | --- |
| Record-level complete | no, aggregate only | yes |
| Identified | 27 as recorded | 38 records under a document-type limit (IEEE Xplore 9, Web of Science 15, Scopus 14) |
| After de-duplication | not separable | 32 distinct |
| Excluded at screening | 6 gate events | 6 (EX-0, not a dataset descriptor) |
| Assessed at full resource | 7 gate events, five repeats | 26 |
| Already in corpus | not applicable | 4 |
| Excluded at full resource | see S4b.1 | 10 (EX-1 1, EX-2 2, EX-3 7) |
| Eligible candidates | not separable | 12 |
| Admitted | Stream A 16 (14 at the census, plus SEED-MYA and SEED-SD added after it), Stream B 6 | 5 after access verification (condition (3), manuscript Section 2.3) |
| Deferred | none recorded | 7, condition (3) unverified |
| Reconciliation | 27 - 8 = 19, does not close | 32 = 6 + 4 + 10 + 12 |

Final corpus: 27 = 22 from pass 1 plus 5 from pass 2.

The 32 distinct pass-2 records are supplied as data/S4b_pass2_records.csv in the reproducibility package (Reproducibility_package.zip), each with its source database or databases, title, year, DOI, stage, disposition and reason, so that the reconciliation 32 = 6 + 4 + 10 + 12 can be checked record by record: 6 records excluded at screening under EX-0 because they are not dataset descriptors, 4 records describing corpora already included, 10 records excluded at full resource (EX-1 1, EX-2 2, EX-3 7) and 12 eligible candidates, of which 5 were admitted after access verification and 7 were deferred with condition (3) unverified.

### S4b.3 Resource-by-resource ledger

| **#** | **Resource** | **Screening** | **Full resource** | **EX code** | **Relation to the 11-item log** | **Final status** |
| --- | --- | --- | --- | --- | --- | --- |
| SA01 | DEAP | PASSED | INCLUDED |  | n/a (included corpus) | INCLUDED |
| SA02 | SEED | PASSED | INCLUDED |  | n/a (included corpus) | INCLUDED |
| SA03 | SEED-IV | PASSED | INCLUDED |  | n/a (included corpus) | INCLUDED |
| SA04 | SEED-V | PASSED | INCLUDED |  | n/a (included corpus) | INCLUDED |
| SA05 | SEED-VII | PASSED | INCLUDED |  | n/a (included corpus) | INCLUDED |
| SA06 | SEED-GER | PASSED | INCLUDED |  | n/a (included corpus) | INCLUDED |
| SA07 | SEED-FRA | PASSED | INCLUDED |  | n/a (included corpus) | INCLUDED |
| SA08 | DREAMER | PASSED | INCLUDED |  | n/a (included corpus) | INCLUDED |
| SA09 | AMIGOS | PASSED | INCLUDED |  | n/a (included corpus) | INCLUDED |
| SA10 | MAHNOB-HCI | PASSED | INCLUDED |  | n/a (included corpus) | INCLUDED |
| SA11 | FACED | PASSED | INCLUDED |  | n/a (included corpus) | INCLUDED |
| SA12 | DENS | PASSED | INCLUDED |  | n/a (included corpus) | INCLUDED |
| SA13 | EVA-MED (Enhanced Valence-Arousal Multimodal Emotion Dataset) | PASSED | INCLUDED |  | n/a (included corpus) | INCLUDED |
| SA14 | Voice-UI EEG Emotion Dataset | PASSED | INCLUDED |  | n/a (included corpus) | INCLUDED |
| SA15 | SEED-MYA | PASSED | INCLUDED |  | n/a (included corpus) | INCLUDED |
| SA16 | SEED-SD | PASSED | INCLUDED |  | n/a (included corpus) | INCLUDED |
| SA17 | Emognition | n/a (identified in pass 2) | INCLUDED (pass 2) |  | n/a | INCLUDED |
| SA18 | K-EmoCon | n/a (identified in pass 2) | INCLUDED (pass 2) |  | n/a | INCLUDED |
| SA19 | ECSMP | n/a (identified in pass 2) | INCLUDED (pass 2) |  | n/a | INCLUDED |
| SA20 | LLaMAC | n/a (identified in pass 2) | INCLUDED (pass 2) |  | n/a | INCLUDED |
| SA21 | Mixed Emotion (Tsinghua) | n/a (identified in pass 2) | INCLUDED (pass 2) |  | n/a | INCLUDED |
| SA22 | CASE | EXCLUDED at screening | EXCLUDED at full-resource | EX-1 | present in the 11-item log | EXCLUDED |
| SA23 | WESAD | EXCLUDED at screening | EXCLUDED at full-resource | EX-1 | present in the 11-item log | EXCLUDED |
| SA24 | VREED | EXCLUDED at screening | EXCLUDED at full-resource | EX-1 | present in the 11-item log | EXCLUDED |
| SA25 | MVRS | EXCLUDED at screening | EXCLUDED at full-resource | EX-1 | present in the 11-item log | EXCLUDED |
| SA26 | DECAF | EXCLUDED at screening | EXCLUDED at full-resource | EX-2 | present in the 11-item log | EXCLUDED |
| SA27 | UNNAMED: title/abstract-level non-emotion catch | EXCLUDED at screening | n/a | EX-0 | absent from the log (unnamed) | EXCLUDED |
| SA28 | SEED-VIG | PASSED | EXCLUDED at full-resource | EX-3 | present in the 11-item log | EXCLUDED |
| SA29 | SEED-DV | PASSED | EXCLUDED at full-resource | EX-3 | present in the 11-item log | EXCLUDED |
| SA30 | NeuroEmo (OpenNeuro ds005700) | not recorded | not recorded | EX-2 (see S6) | an entry of the 11-item log | EXCLUDED |
| SA31 | SEED-VLA / SEED-VRW | not recorded | not recorded | EX-3 (see S6) | an entry of the 11-item log | EXCLUDED |
| SA32 | SEED-Multimodal / MULTI-SEED | not recorded | not recorded | none; not a distinct corpus (see S6) | an entry of the 11-item log | HELD |
| SA33 | EEGEMO (Zenodo 7398263) | not recorded | not recorded | EX-4, held (see S6) | an entry of the 11-item log | HELD |

The ledger has 33 rows: the 29 Stream A resources, 21 included (SA01 to SA21) and 8 departed (SA22 to SA29), and the 4 further entries of the exclusion log, excluded or held, that never entered a Stream A gate (SA30 to SA33). The full ledger, including the descriptor identifier where one is recorded, the evidence for each disposition and the note recording which resources were counted at both gates, is supplied as data/S4b_streamA_ledger.csv in the reproducibility package (Reproducibility_package.zip). Its 29 Stream A rows equal the identification total of Section S4 in number but not in composition: that total is the first-pass aggregate of 27 plus SEED-MYA and SEED-SD, whereas the ledger lists the 22 resources that the census log accounts for, SEED-MYA and SEED-SD, and the 5 corpora of the second search pass.

### S4b.4 Exclusion code EX-0

EX-1 to EX-4 all presuppose that the resource was opened and examined, so none of them can encode a title and abstract exclusion. EX-0 codes exclusions taken at screening, before the resource itself is opened, when the face of the record shows that it does not describe an EEG emotion dataset (Section S0). The one unnamed screening departure in pass 1 (row SA27) was logged at the time without a code, since none of EX-1 to EX-4 could express it; it is coded EX-0 by reason and stage alone, and the pass-1 log preserves no name for it. The six records excluded at screening in pass 2 are not dataset descriptors (methods or secondary-analysis papers that release no new dataset); they are coded EX-0 and listed in data/S4b_pass2_records.csv.

## S5 Per-Dataset Identification Stream & Provenance (All N = 27)

(Supports manuscript Section 3.1.)

| **ID** | **Dataset** | **Year** | **Stream** | **Discovery channel** | **Persistent ID (DOI / accession)** |
| --- | --- | --- | --- | --- | --- |
| 1 | DEAP | 2011 | A | academic-database (seed paper) + citation seed | 10.1109/T-AFFC.2011.15 \| dataset-level PID: NONE (descriptor DOI or laboratory landing page only; verified at audit) |
| 2 | SEED | 2015 | A | academic-database + citation seed + BCMI portal | 10.1109/TAMD.2015.2431497 \| dataset-level PID: NONE (descriptor DOI or laboratory landing page only; verified at audit) |
| 3 | SEED-IV | 2018 | A | academic-database + citation forward + BCMI portal | 10.1109/TCYB.2018.2797176 \| dataset-level PID: NONE (descriptor DOI or laboratory landing page only; verified at audit) |
| 4 | SEED-V | 2022 | A | academic-database + citation forward + BCMI portal | 10.1109/TCDS.2021.3071170 \| dataset-level PID: NONE (descriptor DOI or laboratory landing page only; verified at audit) |
| 5 | SEED-VII | 2024 | A | academic-database + citation forward + BCMI portal | 10.1109/TAFFC.2024.3485057 \| dataset-level PID: NONE (descriptor DOI or laboratory landing page only; verified at audit) |
| 6 | SEED-GER | 2022 | A | academic-database + citation + BCMI portal | 10.1088/1741-2552/ac5c8d \| dataset-level PID: NONE (descriptor DOI or laboratory landing page only; verified at audit) |
| 7 | SEED-FRA | 2022 | A | academic-database + citation + BCMI portal | 10.1088/1741-2552/ac5c8d \| dataset-level PID: NONE (descriptor DOI or laboratory landing page only; verified at audit) |
| 8 | DREAMER | 2017 | A | academic-database + citation seed + Zenodo portal | 10.1109/JBHI.2017.2688239 \| dataset-level PID: NONE (descriptor DOI or laboratory landing page only; verified at audit) |
| 9 | AMIGOS | 2018 | A | academic-database + citation seed | 10.1109/TAFFC.2018.2884461 \| dataset-level PID: NONE (descriptor DOI or laboratory landing page only; verified at audit) |
| 10 | MAHNOB-HCI | 2011 | A | academic-database + citation backward (from DEAP/AMIGOS) | 10.1109/T-AFFC.2011.25 \| dataset-level PID: NONE (descriptor DOI or laboratory landing page only; verified at audit) |
| 11 | FACED | 2023 | A | academic-database (Nature Sci. Data) + citation forward | 10.1038/s41597-023-02650-w \| dataset-level: 10.7303/syn50614194 (verified) |
| 12 | MPED | 2019 | B | citation forward + GitHub portal | 10.1109/ACCESS.2019.2891579 \| dataset-level PID: NONE (descriptor DOI or laboratory landing page only; verified at audit) |
| 13 | GAMEEMO | 2020 | B | citation forward + Mendeley Data portal | 10.17632/b3pn4kwpmn.3 |
| 14 | DENS | 2021 | A | academic-database (bioRxiv / IEEE Access) | 10.1101/2021.08.04.455041 \| dataset-level: 10.18112/openneuro.ds003751.v1.0.2 (verified) |
| 15 | IESD (ds003004) | 2022 | B | data-portal (OpenNeuro/NEMAR) + citation verify | 10.18112/openneuro.ds003004.v1.1.1 |
| 16 | EmoEEG-MC (ds005540) | 2025 | B | data-portal (OpenNeuro) + academic (Nature Sci. Data) | 10.1038/s41597-025-05349-2 \| dataset-level: 10.18112/openneuro.ds005540.v1.0.7 (verified) |
| 17 | EVA-MED | 2025 | A | academic-database (Nature Sci. Data) + arXiv 2503.16584 | 10.1038/s41597-025-06214-y \| dataset-level: 10.57760/sciencedb.23231 (verified) |
| 18 | EAV | 2024 | B | citation forward + Zenodo portal | 10.5281/zenodo.10205702 |
| 19 | Voice-UI EEG | 2024 | A | academic-database (Nature Sci. Data) | 10.1038/s41597-024-03887-9 \| dataset-level: 10.6084/m9.figshare.c.7207839 (verified) |
| 20 | OpenNeuro Film-Clips / Daly (ds002721/ds002722) | 2020 | B | data-portal (OpenNeuro) + citation forward | 10.1038/s41597-020-0507-6 \| dataset-level: 10.18112/openneuro.ds002721.v1.0.3 (verified) |
| 21 | SEED-MYA | 2025 | A | academic-database (IEEE TAFFC / EMBC 2024) + BCMI portal | 10.1109/TAFFC.2025.3586444 (descriptor, verified) \| dataset-level PID: NONE (BCMI landing page https://bcmi.sjtu.edu.cn/home/seed/seed-MYA.html) |
| 22 | SEED-SD | 2025 | A | academic-database (IEEE TAFFC 2025) + BCMI portal | 10.1109/TAFFC.2025.3572504 (descriptor, verified) \| dataset-level PID: NONE (BCMI landing page https://bcmi.sjtu.edu.cn/home/seed/seed-SD.html) |
| 23 | Emognition | 2022 | A | academic-database (Scopus + Web of Science, second search pass) | dataset-level: 10.7910/DVN/R9WAF4 (Harvard Dataverse; verified) |
| 24 | K-EmoCon | 2020 | A | academic-database (Scopus + Web of Science, second search pass) | dataset-level: 10.5281/zenodo.3931963 (Zenodo, version 1.0.0, issued 2020-07-07; resolves in DataCite alone, Section S20) |
| 25 | ECSMP | 2021 | A | academic-database (Scopus + Web of Science, second search pass) | dataset-level: 10.17632/vn5nknh3mn (Mendeley Data concept DOI; verified) |
| 26 | LLaMAC | 2025 | A | academic-database (Scopus, second search pass) | dataset-level: 10.6084/m9.figshare.28748696 (figshare concept DOI; verified) |
| 27 | Mixed Emotion (Tsinghua) | 2024 | A | academic-database (Scopus, second search pass) | dataset-level: 10.5281/zenodo.11194571 (Zenodo, the version named in the descriptor’s Data Records section; verified) |

Stream tally: Stream A before the second search pass = 16 (IDs 1–11, 14, 17, 19, 21, 22); Stream B = 6 (citation: MPED, GAMEEMO, EAV; portal: IESD, EmoEEG-MC, Daly ds002721); Stream A at the second search pass (strings executed on 2026-09-10) = 5 (IDs 23–27). Total included = 27. The five second-pass inclusions are attributed to Stream A because each is returned by at least one Stream A lane. Under the document-type limits of the second pass, Emognition (Saganowski et al., 2022), K-EmoCon (Park et al., 2020) and ECSMP (Gao et al., 2021) are returned by both the Scopus and the Web of Science lanes, and LLaMAC (Lee and Kim, 2025) and Mixed Emotion (Yang et al., 2024) by the Scopus lane. Without any limit, the recorded PubMed string returns all five, and the recorded Semantic Scholar and Nature Scientific Data strings return four of them, ECSMP being the exception in both (strings as recorded in Section S1).

## S6 Datasets Excluded After Full-Census Verification

(Supports manuscript Sections 2.2 and 3.1; the exclusion codes are defined in Section S0.)

Eleven named resources were removed from the appraised corpus after verification: nine excluded on substantive grounds and two held on integrity grounds. Each is recorded once with its disposition code and reason. The one screening exclusion whose name was not retained in the census log is not a row of this table; it is recorded by code (EX-0) and stage in the Stream A ledger (Section S4b, entry SA27). These datasets are never scored, listed among the appraised corpora, or entered into any FAIR/AI-readiness/ontology level count or cluster mean. Note the high-profile affective-computing corpora CASE, WESAD and DECAF, and the non-emotion SEED-family variants.
The Family column also records each resource’s discovery stream, because this log and the Stream A funnel in Section S4 count different populations and the difference is otherwise easy to miss. Four of the eleven named resources have no Stream A gate record at all: three were reached through Stream B portal lanes and one was never a distinct corpus. Reading the log as though every entry had passed through the Stream A gates would make the two sections appear not to reconcile.

| **#** | **Excluded dataset** | **Family; discovery stream** | **EX-code** | **Reason (verified)** |
| --- | --- | --- | --- | --- |
| 1 | CASE | standalone; Stream A | EX-1 | No EEG signal. Peripheral physiology only (ECG/BVP/EMG/EDA/resp/temp @1000 Hz) + continuous joystick V/A. (Sharma et al. 2019, Nature Sci. Data.) |
| 2 | WESAD | standalone; Stream A | EX-1 | No EEG signal. Wrist + chest wearables (BVP/ECG/EDA/EMG/resp/temp/ACC) only; explicitly no EEG. (Schmidt et al. 2018, ICMI.) |
| 3 | DECAF | standalone; Stream A | EX-2 | Non-EEG neuroimaging. Brain signal is MEG (+ NIR face / hEOG / ECG / tEMG), not scalp EEG. (Abadi et al. 2015, IEEE TAC.) |
| 4 | VREED | standalone; Stream A | EX-1 | No EEG signal. VR 360° emotion dataset but only ECG + GSR + eye-tracking; no EEG. Notable exclusion for the VR/neuroarchitecture framing. (2021, ACM IMWUT.) |
| 5 | MVRS | standalone; Stream A | EX-1 | No EEG signal. Eye-tracking / EMG / GSR / body-motion only; no scalp EEG (access also IEEE DataPort subscription, but excluded on EX-1). (2025, IEEE DataPort / arXiv 2509.05330.) |
| 6 | NeuroEmo (OpenNeuro ds005700) | standalone; Stream B portal (OpenNeuro) | EX-2 | Non-EEG neuroimaging. fMRI emotion-recognition dataset; no scalp EEG. Distinguished from Daly ds002725, the joint EEG-fMRI deposit of the included Daly family, which does contain EEG. |
| 7 | SEED-VIG | SEED (BCMI, SJTU); Stream A | EX-3 | No emotion label. 23 subj, 17-ch EEG @1000→200 Hz + EOG, but labels = PERCLOS vigilance/drowsiness in a virtual-driving task = arousal-of-driving, not emotion. SEED-VIG2 multimodal extension is also vigilance → same EX-3. |
| 8 | SEED-VLA / SEED-VRW | SEED (BCMI, SJTU); Stream B portal (BCMI) | EX-3 | No emotion label. Driving-fatigue/vigilance EEG (SEED-VLA lab dry-electrode 20× .edf @300 Hz; SEED-VRW real-world 14× .edf); continuous vigilance labels 0–1, no emotion annotation. |
| 9 | SEED-DV (EEG2Video) | SEED (BCMI, SJTU); Stream A | EX-3 | No emotion label. 20 subj, 62-ch @200 Hz, but task = dynamic-video / visual-perception decoding over 40 visual concepts (1400 clips); no affect annotation. (NeurIPS 2024.) |
| 10 | SEED-Multimodal / MULTI-SEED | SEED (BCMI, SJTU); none (not a distinct corpus) | Not a distinct corpus | No published BCMI dataset under these names confirmed; “multimodal” merely describes SEED-IV/V/VII (EEG + eye-movement). Treated as non-existent; not scored. |
| 11 | EEGEMO (Zenodo 7398263) | standalone; Stream B portal (Zenodo) | EX-4 (held) | Record public (15 subj, Muse S + Neurosity Crown, 16 videos, V-A) but “files restricted to users with access”; universal grantability not confirmed → data not demonstrably obtainable internationally. Held pending an access test; not scored. |

SEED-family disposition summary. Included as separate corpus entries: SEED (3-class), SEED-IV (4-class), SEED-V (5-class), SEED-VII (7-state + continuous intensity), SEED-GER and SEED-FRA (cross-cultural 3-class), SEED-MYA (3-class, Myanmar cohort) and SEED-SD (4-class plus 1–5 intensity, sleep-deprivation design). Excluded with stated reasons: SEED-VIG, SEED-VLA/VRW, SEED-DV (all EX-3, no emotion label); SEED-Multimodal/MULTI-SEED (not a distinct corpus). No SEED variant is silently dropped.
Population reconciliation. The eleven named entries decompose as 7 resources that departed Stream A and are named at its gates, 3 resources reached through Stream B portal lanes and excluded there, and 1 name that proved not to denote a distinct corpus, giving 7 + 3 + 1 = 11. Section S4 counts only the first of these three groups, which is why its funnel and this log cannot be reconciled by subtraction. With the unnamed EX-0 entry (Section S4b), every resource in the Stream A funnel of Section S4 and in the Stream B lanes carries a unique final disposition, by name in all cases but one, and by reason and stage in that one. The records returned by the second search pass, including seven eligible candidates deferred with condition (3) unverified, are accounted for in Section S4b.

## S7 Datasheets for Non-Paper Stream-B Datasets (Gebru et al. 2021 style)

(Supports manuscript Section 2.2.)

The Stream-B portal-discovered datasets are documented with abbreviated datasheets following the Gebru et al. (2021) Datasheets for Datasets schema (Motivation, Composition, Collection, Preprocessing/Labelling, Uses, Distribution, Maintenance). These three corpora were surfaced primarily through data-portal browsing rather than a conventional seed-paper database hit, so an explicit datasheet is provided for verifiability. (The citation-discovered Stream-B datasets MPED, GAMEEMO and EAV each have a peer-reviewed primary publication and are documented through it.)

### S7.1 IESD (Imagined Emotion Study, OpenNeuro ds003004)

Motivation. Capture self-induced (imagined) emotional states via high-density EEG; foundational study Onton & Makeig (2009), Front. Hum. Neurosci. 3:61.

Composition. 34 subjects; 250-channel BioSemi ActiveTwo (256 Hz); 15 discrete self-induced emotional states (anger, awe, compassion, contentedness, disgust, excitement, fear, frustration, grief, happiness, jealousy, joy, love, relief, sadness); EEG only.

Collection. Audio-guided imagination protocol; eyes-closed emotion-imagination task.

Preprocessing / labelling. Raw + ICA-decomposable; self-reported emotional category per induction. Single-rater self-induced design (no external rater).

Uses. Discrete emotion classification; imagined-affect decoding, directly relevant to imagining/recalling spatial affect.

Distribution. FullyOpen; CC0; BIDS-EEG; OpenNeuro accession ds003004 (DOI 10.18112/openneuro.ds003004.v1.1.1); mirrored on NEMAR.

Maintenance. OpenNeuro repository; SCCN, UC San Diego. Versioned (v1.1.0 2020 → v1.1.1 2022).

### S7.2 EmoEEG-MC (Multi-Context Emotional EEG, OpenNeuro ds005540)

Motivation. Enable cross-context emotion decoding (video-induced vs imagery-induced); descriptor Xu et al. (2025), Nature Sci. Data 12:1142.

Composition. 60 participants; 64-channel EEG + peripheral physiology; 7 emotion categories (joy, inspiration, tenderness, fear, disgust, sadness, neutral) + dimensional labels; two elicitation contexts (video, imagery).

Collection. Southern University of Science and Technology (SUSTech, NCC Lab, Quanying Liu); video and mental-imagery elicitation; subjective validation reports.

Preprocessing / labelling. BIDS-EEG sidecar metadata; mixed discrete + dimensional labels; cross-context design.

Uses. Cross-context emotion decoding; transfer-learning base; imagery context ≈ imagined spatial affect.

Distribution. FullyOpen; CC-type; BIDS-EEG; OpenNeuro ds005540 (DOI 10.18112/openneuro.ds005540.v1.0.7); ScienceDB DOI 10.57760/sciencedb.14025; code at github.com/ncclab-sustech/EmoEEG-MC.

Maintenance. OpenNeuro + ScienceDB; SUSTech NCC Lab. The corpus closest to L4 in the included set (formal BIDS schema + rich mixed labels + datasheet); scored L3 because a standard data schema is not a semantic model.

### S7.3 OpenNeuro Film-Clips / Daly Affective-Music family (ds002721 / ds002722; joint EEG-fMRI ds002725)

Motivation. Investigate neural/physiological correlates of affect induced by music; descriptor Daly et al. (2020), Nature Sci. Data 7:177.

Composition. 31 subjects; 19-channel 10-20 EEG (FCz reference) in the scored deposit ds002721; companion accessions ds002722 (EEG only) and ds002725 (joint EEG-fMRI, EEG present); 8 self-rated induced-emotion dimensions (pleasantness, energy, sadness, anger, tenderness, happiness, fear, tension).

Collection. Affective musical-stimulus listening with synchronised physiological/neurological recording and self-reported affect.

Preprocessing / labelling. BIDS-EEG; per-trial self-reported ratings on the eight dimensions (9-point scale), embedded as trigger codes in the event files; multi-dimensional rating profile.

Uses. Music-affect decoding; high-FAIR transfer anchor; music-induced affect ratings, from which valence and arousal must be derived, transferable to ambient-environment mood for spatial-emotion modelling.

Distribution. FullyOpen; CC0; BIDS-EEG; OpenNeuro ds002721 (DataCite DOI 10.18112/openneuro.ds002721.v1.0.3), the scored unit. The companion accession ds002722 (DataCite DOI 10.18112/openneuro.ds002722.v1.0.0, registry title “phBCMIcalibration”) belongs to the same Daly Affective-Music family and is recorded in Section S20 but not scored separately.

Maintenance. OpenNeuro repository; 7-author roster matched across descriptor and DataCite.

Ontology terms in the descriptor record. The data descriptor of the Daly family deposits a machine-readable ISA-Tab record on figshare (10.6084/m9.figshare.12326519). Its ontologySourceReferences block names ENVO, OBI, EFO, MMO, ERO, VT, CHMO and NCBITAXON, and its terms resolve as PURLs under http://purl.obolibrary.org/obo/ (VT_0002572, EFO_0004464, MMO_0000023 and GO_0050896). The record nonetheless fails the Level 4 test on its own content, for three independent reasons. Its terms annotate measurement type, technology type and factor type, not the affect label vocabulary. It is shared across the six OpenNeuro deposits listed by the descriptor record (ds002720 to ds002725) rather than describing ds002721. It is lodged with the article on figshare, not inside the deposit. Within ds002721, a search of the event sidecars for ontology, HED, EmotionML and MFOEM terms returns nothing, and neither the eight emotion adjectives nor the nine-point response scale is mapped to any external term. The Level 4 gate therefore closes on positive evidence, not on a failure to retrieve. L4 = 0 is accordingly a finding about the corpus (manuscript Section 3.4), not a limit on what could be inspected.

## S8 Datasets Included via Citation Searching (Stream B, n = 3)

(Supports manuscript Section 2.2.)

These three datasets were added through citation searching (forward citation traversal from the Stream A anchors), de-duplicated against the Stream A seed/anchor corpora, and then verified for existence and universal accessibility. The discovery-channel column records the full provenance.

| **ID** | **Dataset** | **Year** | **Discovery channel** | **Access class** | **Persistent ID** |
| --- | --- | --- | --- | --- | --- |
| 12 | MPED | 2019 | citation forward (from SEED/DEAP network) + GitHub portal | Academic (GitHub request) | 10.1109/ACCESS.2019.2891579 |
| 13 | GAMEEMO | 2020 | citation forward + Mendeley Data portal | FullyOpen (CC-BY 4.0) | 10.17632/b3pn4kwpmn.3 |
| 18 | EAV | 2024 | citation forward + Zenodo portal | Conditional (CC-BY-4.0, DUA) | 10.5281/zenodo.10205702 |

The three OpenNeuro/portal-discovered datasets (IESD, EmoEEG-MC, Daly ds002721) are attributed to Stream B data-portal browsing, not citation searching, and are documented in Section S7. The five anchor corpora reached by citation searching (DEAP, SEED family, AMIGOS, DREAMER, FACED) are attributed to Stream A and are not re-counted here.

## S9 PRISMA 2020 Checklist Mapping (27 items)

(Supports the whole manuscript.)

| **#** | **PRISMA 2020 item** | **Reported location** |
| --- | --- | --- |
| 1 | Title, identify as systematic review | Title |
| 2 | Abstract, structured summary | Abstract |
| 3 | Rationale | Section 1 (Introduction) |
| 4 | Objectives (RQ1–RQ3) | Section 1; Section 2.1 |
| 5 | Eligibility criteria | Section 2.3; Supp S0, S13 |
| 6 | Information sources | Section 2.2; Supp S1, S2, S3, S3b |
| 7 | Search strategy (full strings) | Section 2.2; Supp S1, S2 (PRISMA-S Item 4) |
| 8 | Selection process | Section 2.2; Supp S4, S4b, S5 |
| 9 | Data collection process | Sections 2.4 and 2.6 |
| 10 | Data items | Section 2.4 and Table 1; Supp S5, S16 |
| 11 | Study/dataset risk-of-bias assessment | Section 2.3; Supp S12 (QUADAS-2-adapted) |
| 12 | Effect measures (quality axes: FAIR / AI-readiness / OFI) | Section 2.4; Sections 3.2 to 3.5 |
| 13 | Synthesis methods | Section 2.4; Section 3 |
| 14 | Reporting-bias assessment | Section 2.3; Supp S12, domain D3 (selective documentation by dataset releasers, the dataset analogue of selective outcome reporting) |
| 15 | Certainty assessment (reliability κ; weight sensitivity) | Sections 2.4.2 and 2.5; Supp S11, S14 |
| 16a | Study/dataset selection results | Section 3.1; Supp S4, S4b, S5; Figure 3 (PRISMA flow) |
| 16b | Excluded resources with reasons | Sections 2.2 and 3.1; Supp S0, S4b, S6 |
| 17 | Dataset characteristics | Section 3.1 and Table 1; Supp S16 |
| 18 | Risk-of-bias results | Sections 2.3 and 4.6; Supp S12 |
| 19 | Results of individual datasets | Sections 3.2 to 3.5; Supp S15, S17, S18, S19 |
| 20 | Results of syntheses (FAIR / AI-readiness / ontology) | Sections 3.2 to 3.5 |
| 21 | Reporting-bias results | Section 2.3; Supp S12, domain D3 ratings (Sections S12.1 and S12.2) |
| 22 | Certainty-of-evidence results | Sections 2.5 and 4.6; Supp S11 (codebook reproducibility by three language-model rater personas, n = 27: mean item-level Fleiss’ κ = 0.947 over the 49 items with a defined coefficient, bootstrap 95% interval 0.918 to 0.963; per-item coefficients in workbook sheet S11_reliability; raw codings in workbook sheet S11_codings and in data/s11_codings_v2.json of the reproducibility package (Reproducibility_package.zip), from which scripts/reliability.py regenerates every coefficient); S14 (weight sensitivity: Spearman ρ ≥ 0.982 under ±0.10 perturbation of the equal weights) |
| 23a | Discussion, summary of evidence | Section 4 |
| 23b, 23c | Limitations | Section 4.4 (retrieval coverage) and Section 4.6 (limitations, incl. time-specific scoring evidence, the equal-weighting default and weight sensitivity, codebook reproducibility, correlational validity and selection validity); Supp S4 and S4b (counts reported at the stage at which they were recorded); S21 (external coverage reconciliation) |
| 23d | Conclusions and implications | Section 5 (Conclusion) |
| 24 to 27 | Registration / protocol / funding / interests; availability of data, code and other materials | Section 2.1 (not registered; no protocol deposited); front matter / declarations; Data Availability Statement; transparency workbook; reproducibility package (Reproducibility_package.zip), contents described in Supp S24.5 |

## S10 PICOD Framework Application Record

(Supports manuscript Section 2.1; concept diagram = Figure 1.)

Traditional PICO was built for clinical-intervention reviews. For a dataset-resource review we extend it with an explicit D (Dataset constraints) facet. The facet-by-facet application record follows.

### S10.1 P (Population): entities the dataset records

Human emotional / affective states captured in scalp EEG during emotion elicitation (film clips, music videos, images, VR/360° scenes, naturalistic interaction/walking), labelled with discrete emotion categories or with dimensional affect ratings (for example valence, arousal and dominance). Multi-subject cohorts (cross-subject generalisation is a population property) and optional concurrent peripheral/behavioural channels (ECG, GSR/EDA, eye-tracking, facial video) are admissible when EEG is present. Application result: all 27 included corpora contain scalp EEG with emotion/affect labels; subject counts range from 8 (SEED-GER, SEED-FRA) to 123 (FACED).

### S10.2 I (Intervention): AI/ML methods the dataset supports

EEG emotion classification (discrete n-class), V/A(/D) regression, cross-subject generalisation, cross-corpus / transfer learning, multimodal fusion, and continuous affect tracking; support for standard benchmark splits and training-framework readers (TorchEEG, EEGain, braindecode, MNE). Application result: scored on the Task-Alignment (TAS) axis (corpus mean 2.719; led by FACED at 3.5, followed by AMIGOS, DEAP, DENS, DREAMER, EmoEEG-MC, MAHNOB-HCI and SEED-V at 3.2).

### S10.3 C (Comparator): inter-dataset comparison

Prior benchmark EEG emotion corpora (DEAP, SEED family, AMIGOS, DREAMER, FACED) and inter-dataset comparison of scale (subjects × channels × trials), montage density (14 / 32 / 62 / 128 / 250-channel), modality breadth, label richness, and licensing/openness. Application result: comparison is by quality axis, not by reported model accuracy (performance is not a fixed dataset property).

### S10.4 O (Outcome): multidimensional dataset quality

The three-axis quality profile, openness (FAIR) + AI-readiness (AS + TAS + OMS, equally weighted) + ontology maturity (5-level + OFI), plus a QUADAS-2-adapted risk-of-bias profile. Application result (canonical): FAIR means F = 3.311, A = 3.511, I = 2.044, R = 2.563 (composite 2.857); AI-readiness median = 2.60, mean = 2.684, reported as a continuous composite with its rank ordering (manuscript Sections 2.4.2 and 3.3); ontology levels L1 4 / L2 7 / L3 16 / L4 0 / L5 0. Outcome is dataset readiness, never model accuracy.

### S10.5 D (Dataset constraints): explicit eligibility thresholds

Must contain (1) scalp EEG AND (2) emotion/affect labels; released with verifiable persistent documentation (peer-reviewed paper / datasheet / persistent ID); and universally accessible regardless of nationality (a universally grantable subscription, registration, licence or DUA route is permitted; nationality- or geography-based gating is not). No fixed sample-size floor (subject and trial counts are scored under AS/TAS, not used as a cut-off). Application result: 11 named resources failed the D facet and were excluded or held (Section S6): EX-1 no EEG (CASE, WESAD, VREED, MVRS), EX-2 non-EEG neuroimaging (DECAF, NeuroEmo), EX-3 no emotion label (SEED-VIG, SEED-VLA/VRW, SEED-DV), EX-4 / held on accessibility (EEGEMO), and one name that does not denote a distinct corpus (SEED-Multimodal/MULTI-SEED).

### S10.6 PICOD process flow

Define facets: P, I, C, O, D fixed before searching (Figure 1 concept diagram, 5 facet boxes).

Apply D-gate at identification: EEG + emotion-label precondition applied at screening (Stream A: 29 → 23 after 6 screening exclusions).

Apply the two inclusion criteria of Section S0 at full census: persistent documentation + universal accessibility verified per resource (Stream A: 23 → 16; 7 full-text exclusions, 0 accessibility losses).

Augment via Stream B: citation searching (3) + portal browsing (3) add net-new resources, de-duplicated against Stream A anchors.

Extend via the second search pass: the recorded Stream A strings, executed again on 2026-09-10 under database-level document-type limits, add 5 eligible resources (Emognition, K-EmoCon, ECSMP, LLaMAC and Mixed Emotion), each verified against the same criteria and attributed to Stream A (Sections S1 and S4b).

Score on O axes: only the 27 included resources scored on FAIR / AI-readiness / ontology; excluded resources never scored.

Synthesise against I and C: outcomes compared across datasets and clusters, framed for the neuroarchitecture / spatial-emotion transfer question (RQ1–RQ3).

PICOD is proposed as a standard reporting facet for this class of dataset-resource review; this record fulfils the facet-by-facet application required by manuscript Section 2.1.

# Supplementary Material, Sections S11–S19

EEG-based emotion-recognition datasets for neuroarchitecture / spatial-emotion research. Companion to the main manuscript (PRISMA 2020 systematic review). Author-year citations used in this supplement resolve against the reference list of the main manuscript. All statistics are derived deterministically from the canonical corpus (N = 27 included EEG emotion datasets; 11 excluded or held). Excluded datasets are never scored, listed among the appraised corpora, or entered into any cell.

This part reports the codebook-reproducibility experiment in which three language-model rater personas coded all 27 datasets (S11), the per-dataset QUADAS-2-adapted risk-of-bias matrix (S12), the per-dataset eligibility assessment (S13), the AI-readiness criterion weighting and weight-sensitivity analysis (S14), the per-dataset AI-readiness scorecard (S15), a structured overview of all 27 datasets (S16) and the per-dataset FAIR, AI-readiness and ontology-formalisation scores (S17 to S19).

## **S11 Codebook-Reproducibility Experiment with Three Language-Model Rater Personas (n = 27)**

Back-reference: manuscript Section 2.5 (Verification of identifiers, coverage and codebook reproducibility), Section 2.6 (Use of AI tools) and Section 4.6 (Limitations).

**Design.**

Unit of rating = dataset, n = 27, a complete census of the final corpus with no sampling.

Raters = three language-model rater personas (E1 EEG and affective computing, E2 machine-learning task design, E3 knowledge-based design), run in separate contexts, blind to one another, blind to the codings recorded by the review, and given no answer key. The personas were run on Anthropic Claude, accessed through the Claude Code environment (manuscript Section 2.6).

Evidence = the frozen canonical attribute record for each dataset. No web lookup was permitted, so the three language-model raters read identical evidence.

Point values were masked in the rater prompts, so a rater selects a state and never sees what the state is worth. Selecting states while reading their point values would bias the coding towards a target score.

Completion = 4,212 criterion codings (27 datasets × 3 raters × 52 atomic criteria) plus one ontology-level assignment per rater and dataset (81 further judgements), giving 4,293 judgements over 53 items with no missing cell.

Measurement layer. The experiment reports agreement on the atomic states of the codebook in Section S24, not on the scores. Under the deterministic codebook a score is an arithmetic function of the states, so agreement on scores would be 1.0 by construction and would say nothing about the coding. The state layer is the only layer at which the question has content.

**Results.**

Primary coefficient: mean Fleiss kappa over the 49 items with a defined coefficient = 0.947, with a percentile bootstrap 95% interval of [0.918, 0.963] from 10,000 replicates at seed 42, the resampling unit being the dataset. Mean observed agreement p_o = 0.974.

Prevalence-resistant coefficients: Gwet AC1 = 0.962, Brennan-Prediger = 0.958, pooled Fleiss kappa = 0.941.

Non-unanimous items: excluding the items on which the three language-model raters were unanimous, the mean kappa is 0.895. This figure shows how unanimity affects the overall mean and is reported alongside the headline; it is not a statistical lower confidence bound.

Undefined items: 4 of 53 items (F3, A3, SRS-4, ONT-C1) carry no variance, every rater having chosen the same state for every dataset. Their kappa is reported as undefined rather than inflated to 1.00.

Weakest item: RFS-5 at kappa = 0.769. The pre-specified single-item review trigger (kappa < 0.40) did not fire on any item, and the pre-specified acceptance threshold (mean kappa ≥ 0.60) is met.

Interpretation. The coefficient describes agreement on atomic criterion states that three language-model raters read from one shared extracted record. It is evidence of codebook reproducibility, that is, that the written decision rules lead independent applications to the same states. It is not evidence of extraction reliability, because an error in the shared record would be repeated by all three language-model raters, and it is not a human validation of the instrument.

Declared limitation, stated before the coding and retained regardless of the result. The three personas share one base model, so their errors are not independent in the sense the Fleiss model assumes. This coefficient is the reproducibility of the codebook under repeated application, not human inter-rater reliability, and this review does not present it as the latter.

**Supplementary Table S11. Per-item agreement, three language-model raters over 27 datasets. Categories is the number of distinct states selected for an item across all raters and datasets; LEVEL is the ontology-level assignment.**

| **Item** | **Categories** | **p_o** | **Fleiss kappa** | **Gwet AC1** | **Brennan-Prediger** |
| --- | --- | --- | --- | --- | --- |
| F1 | 3 | 1.000 | 1.000 | 1.000 | 1.000 |
| F2 | 2 | 1.000 | 1.000 | 1.000 | 1.000 |
| F3 | 1 | 1.000 | undefined (no variance) | undefined | undefined |
| F4 | 2 | 1.000 | 1.000 | 1.000 | 1.000 |
| A1 | 3 | 1.000 | 1.000 | 1.000 | 1.000 |
| A2 | 3 | 1.000 | 1.000 | 1.000 | 1.000 |
| A3 | 1 | 1.000 | undefined (no variance) | undefined | undefined |
| A4 | 2 | 1.000 | 1.000 | 1.000 | 1.000 |
| A5 | 4 | 1.000 | 1.000 | 1.000 | 1.000 |
| I1 | 3 | 0.951 | 0.920 | 0.928 | 0.926 |
| I2 | 3 | 1.000 | 1.000 | 1.000 | 1.000 |
| I3 | 2 | 0.951 | 0.827 | 0.931 | 0.901 |
| I4 | 3 | 0.951 | 0.904 | 0.934 | 0.926 |
| I5 | 3 | 0.951 | 0.918 | 0.929 | 0.926 |
| R1 | 5 | 1.000 | 1.000 | 1.000 | 1.000 |
| R2 | 2 | 0.975 | 0.914 | 0.965 | 0.951 |
| R3 | 3 | 0.926 | 0.844 | 0.903 | 0.889 |
| R4 | 2 | 1.000 | 1.000 | 1.000 | 1.000 |
| AS1 | 4 | 1.000 | 1.000 | 1.000 | 1.000 |
| AS2 | 2 | 1.000 | 1.000 | 1.000 | 1.000 |
| AS3 | 4 | 0.951 | 0.920 | 0.938 | 0.934 |
| AS4 | 5 | 0.975 | 0.967 | 0.970 | 0.969 |
| AS5 | 5 | 0.975 | 0.920 | 0.973 | 0.969 |
| TAS-1 | 2 | 1.000 | 1.000 | 1.000 | 1.000 |
| TAS-2 | 2 | 1.000 | 1.000 | 1.000 | 1.000 |
| TAS-3 | 2 | 1.000 | 1.000 | 1.000 | 1.000 |
| TAS-4 | 2 | 1.000 | 1.000 | 1.000 | 1.000 |
| TAS-5 | 4 | 0.901 | 0.864 | 0.870 | 0.868 |
| TAS-6 | 4 | 0.975 | 0.963 | 0.968 | 0.967 |
| OMS-1 | 4 | 1.000 | 1.000 | 1.000 | 1.000 |
| OMS-2 | 3 | 0.926 | 0.862 | 0.899 | 0.889 |
| OMS-3 | 3 | 1.000 | 1.000 | 1.000 | 1.000 |
| OMS-4 | 3 | 1.000 | 1.000 | 1.000 | 1.000 |
| OMS-5 | 2 | 1.000 | 1.000 | 1.000 | 1.000 |
| SRS-1 | 3 | 0.975 | 0.955 | 0.966 | 0.963 |
| SRS-2 | 3 | 0.951 | 0.913 | 0.931 | 0.926 |
| SRS-3 | 2 | 0.975 | 0.951 | 0.951 | 0.951 |
| SRS-4 | 1 | 1.000 | undefined (no variance) | undefined | undefined |
| RFS-1 | 4 | 0.975 | 0.959 | 0.969 | 0.967 |
| RFS-2 | 3 | 0.926 | 0.859 | 0.900 | 0.889 |
| RFS-3 | 3 | 0.926 | 0.884 | 0.891 | 0.889 |
| RFS-5 | 2 | 0.901 | 0.769 | 0.828 | 0.802 |
| INT-1 | 3 | 1.000 | 1.000 | 1.000 | 1.000 |
| INT-2 | 3 | 1.000 | 1.000 | 1.000 | 1.000 |
| INT-3 | 3 | 0.951 | 0.920 | 0.928 | 0.926 |
| INT-4 | 3 | 1.000 | 1.000 | 1.000 | 1.000 |
| ONT-C1 | 1 | 1.000 | undefined (no variance) | undefined | undefined |
| ONT-C2 | 6 | 0.914 | 0.865 | 0.901 | 0.896 |
| ONT-C3 | 3 | 1.000 | 1.000 | 1.000 | 1.000 |
| ONT-C4 | 4 | 0.889 | 0.816 | 0.861 | 0.852 |
| ONT-C5 | 5 | 0.963 | 0.940 | 0.956 | 0.954 |
| ONT-C7 | 4 | 0.901 | 0.778 | 0.884 | 0.868 |
| LEVEL | 3 | 0.975 | 0.955 | 0.966 | 0.963 |

The transparency workbook publishes the per-item coefficients in sheet S11_reliability and the 4,293 raw codings, one row per rater, dataset and item, in sheet S11_codings. The same codings are supplied as data/s11_codings_v2.json in the reproducibility package (Reproducibility_package.zip), and the coefficients as data/s11_reliability_v2.json. Every coefficient above can therefore be regenerated from the raw codings rather than read from a summary: from the package root, python3 scripts/reliability.py --check recomputes the per-item coefficients and compares them with data/s11_reliability_v2.json, and python3 scripts/reliability.py --boot 10000 --seed 42 reproduces the bootstrap interval.

## S12 Per-Dataset QUADAS-2-Adapted Risk-of-Bias Matrix (n = 27)

Back-reference: manuscript Section 2.3 (Eligibility and risk of bias).

Each included dataset is appraised across four QUADAS-2-adapted risk-of-bias (RoB) domains plus one applicability-concern judgement. Levels: Low = transparent, standards-conformant, fully reproducible; Moderate = partial gaps or proprietary/EULA-gated elements; High = substantive gap threatening reproducibility. Applicability concern reflects fit of the corpus to the neuroarchitecture / spatial-emotion review question (RQ scope), not signal quality. Excluded datasets are not scored.

RoB domains adapted from QUADAS-2 to a dataset-resource unit of analysis:

D1: Dataset construction (≈ Patient Selection): sampling/cohort design, montage density and standardisation, elicitation-paradigm validity, preprocessing transparency.

D2: Annotation process (≈ Index Test): label model, rater design (self / external / multi-rater), inter-annotator agreement, label provenance.

D3: Documentation / transparency (≈ Reference Standard): datasheet / data descriptor, metadata completeness, verified persistent ID, montage/format documentation. Selective outcome reporting does not arise when the unit of appraisal is a dataset; its analogue, selective documentation by the dataset releaser, is assessed in this domain (reporting bias, manuscript Section 2.3).

D4: Accessibility / reproducibility (≈ Flow & Timing): open vs gated access, licence clarity, format openness/interoperability (BIDS/CC0 vs proprietary .mat-only), retrievability.

| **ID** | **Dataset** | **D1 Construction** | **D2 Annotation** | **D3 Documentation** | **D4 Accessibility** | **Applicability concern** |
| --- | --- | --- | --- | --- | --- | --- |
| 1 | DEAP | Low | Low | Moderate | Moderate | Low |
| 2 | SEED | Low | Moderate | Moderate | Moderate | Low |
| 3 | SEED-IV | Low | Moderate | Moderate | Moderate | Low |
| 4 | SEED-V | Low | Low | Moderate | Moderate | Low |
| 5 | SEED-VII | Low | Low | Moderate | Moderate | Low |
| 6 | SEED-GER | Moderate | Moderate | Moderate | Moderate | Moderate |
| 7 | SEED-FRA | Moderate | Moderate | Moderate | Moderate | Moderate |
| 8 | DREAMER | Moderate | Low | Low | Moderate | Moderate |
| 9 | AMIGOS | Moderate | Low | Moderate | Moderate | Moderate |
| 10 | MAHNOB-HCI | Low | Low | Moderate | Moderate | Low |
| 11 | FACED | Low | Low | Low | Moderate | Low |
| 12 | MPED | Low | Moderate | Moderate | High | Low |
| 13 | GAMEEMO | Moderate | Low | Moderate | Low | Moderate |
| 14 | DENS | Low | Low | Moderate | Moderate | Low |
| 15 | IESD (OpenNeuro ds003004) | Low | Moderate | Moderate | Low | Moderate |
| 16 | EmoEEG-MC (ds005540) | Low | Low | Low | Low | Low |
| 17 | EVA-MED | Low | Low | Moderate | Moderate | Moderate |
| 18 | EAV | Low | Low | Low | Moderate | Moderate |
| 19 | Voice-UI EEG | Low | Low | Low | Moderate | Moderate |
| 20 | OpenNeuro Film Clips / Daly (ds002721/ds002722) | Low | Low | Low | Low | Low |
| 21 | SEED-MYA | Low | Moderate | Moderate | Moderate | Moderate |
| 22 | SEED-SD | Low | Low | Moderate | Moderate | Low |
| 23 | Emognition | Moderate | Low | Low | Moderate | Moderate |
| 24 | K-EmoCon | Moderate | Low | Low | Moderate | Moderate |
| 25 | ECSMP | Moderate | Low | Low | Moderate | Moderate |
| 26 | LLaMAC | Moderate | Low | Low | Low | Moderate |
| 27 | Mixed Emotion (Tsinghua) | Low | Low | Low | Moderate | Low |

### S12.1 Per-domain rationale (condensed)

The rules below are applied as written to the source-verified attribute record (Section S16 and data/attributes.json). Where a corpus meets both a Moderate condition and the description of Low, the Moderate condition governs, and where a rule leaves a term undefined, its most literal reading is applied and stated with the rule.

D1 Moderate where cohort is small / culturally narrow or montage is low-density: SEED-GER and SEED-FRA (n = 8 each), DREAMER and AMIGOS (14-ch consumer Emotiv), GAMEEMO (14-ch Emotiv, game-induced surrogate labels). Among the five corpora identified by the second search pass (2026-09-10), the same trigger fires for Emognition (4 dry channels on a Muse 2 headband), K-EmoCon (consumer NeuroSky single-channel headset in a naturalistic setting), ECSMP (seven forehead channels, with per-subject variation in the released channel set) and LLaMAC (14-ch consumer Emotiv), whereas Mixed Emotion (Tsinghua) is Low on a 21-channel 10-20 montage with a released preprocessing pipeline. All other corpora are full-density / standard-montage with documented preprocessing → Low, including EVA-MED (64 channels, 64 participants) and Voice-UI EEG (63 channels acquired and 57 released, 44 participants deposited), whose channel counts are those of the source-verified attribute record (Section S16). The rule states no numeric threshold and is applied at its anchors: the cohorts rated Moderate on size have 8 participants and the smallest cohort rated Low has 15; the 14-channel consumer headsets and smaller montages are low-density, whereas the 21-channel 10-20 montage of Mixed Emotion is not, and the 19-channel Daly montage, the standard 10-20 electrode set, is rated Low as a standard montage. A culturally narrow cohort is not applied as a trigger on its own, because its most literal reading, recruitment in a single country, would describe almost every corpus, including Mixed Emotion, which the rule rates Low.

D2 Moderate where labels are flat discrete with single-rater self-report and no reported inter-annotator agreement: SEED / SEED-IV (3- and 4-class flat), SEED-GER/FRA/MYA, MPED (7-class single-rater), IESD (15 self-induced imagined states, no external rater); a class code defined by the stimulus and released without any rating is read as meeting this condition. D2 Low where multi-rater (self + external: AMIGOS) or rich mixed discrete + dimensional with continuous intensity (SEED-VII, FACED, EmoEEG-MC, EVA-MED, EAV, MAHNOB-HCI keywords plus V-A-D, DREAMER full V-A-D, DENS per-clip V-A-D plus event-localised discrete choices, SEED-V 5-class with a per-trial self-rated induction score (0 to 5), SEED-SD 4-class with self-rated intensity, Daly 8-dim, DEAP V-A-D). Because this list includes purely dimensional label sets (DREAMER, Daly, DEAP), Low is read as applying to every released label set that is not flat discrete, and on that reading two further corpora are D2 Low: GAMEEMO, whose four stimulus-defined game conditions are released with five 1 to 10 ratings and 5-point SAM valence and arousal ratings per session, although only as PDF forms (the rule does not distinguish machine-readable from scanned rating forms), and Voice-UI EEG, whose labels are single-rater 7-point ratings on nine emotion-related adjective pairs with two derived factor scores. All five corpora from the second search pass are D2 Low: K-EmoCon on multi-rater design (self, partner and five external observers), Emognition, LLaMAC and Mixed Emotion on rich mixed discrete plus dimensional labels, and ECSMP on six discrete classes, each with a self-rated intensity.

D3 Moderate where no formal datasheet / data descriptor accompanies the release or the PID is a preprint / portal-only record: DEAP, the SEED family (BCMI portal, no datasheet), AMIGOS and MAHNOB-HCI (laboratory portal, no dataset-level identifier), MPED (GitHub README only), DENS (bioRxiv PID) and IESD (BIDS sidecar metadata, but no datasheet or data descriptor). The portal-only records, with no dataset-level identifier and the data reached only through a laboratory or project page, are DEAP, the eight SEED-family corpora, AMIGOS, MAHNOB-HCI and MPED; several of them have a peer-reviewed descriptor, SEED-VII, AMIGOS and MAHNOB-HCI among them, but the rule states the portal-only condition without exception, so a descriptor does not make a portal-only record Low. D3 Low where a peer-reviewed data descriptor (Nature Sci Data / IEEE TAFFC) and complete metadata exist, read as a descriptor in any peer-reviewed venue together with an EEG channel count and EEG sampling rate declared by the releaser (FACED, EmoEEG-MC / Daly BIDS sidecar metadata, DREAMER Zenodo record, EAV Zenodo, Voice-UI EEG figshare). EVA-MED and GAMEEMO have descriptors but do not meet the complete-metadata condition and are D3 Moderate: EVA-MED reports no EEG sampling rate and holds its channel list only inside the restricted deposit, and the GAMEEMO descriptor is closed access while its Mendeley deposit carries no README, no data dictionary and no EEG sampling rate. Of the five corpora from the second search pass, Emognition, K-EmoCon, LLaMAC and Mixed Emotion are D3 Low on peer-reviewed Scientific Data descriptors with resolvable deposit identifiers, and ECSMP is D3 Low on its Data in Brief descriptor, its Mendeley Data identifier and the event-code dictionary (event.txt) distributed with the data.

D4 High = MPED only: GitHub-hosted with an unverified data-availability request route → reproducibility not demonstrably guaranteed. D4 Moderate for EULA / registration / application-gated academic corpora (DEAP, SEED family, AMIGOS, MAHNOB-HCI, EAV DUA-approval) and for FACED, whose Synapse deposit is listable without an account but requires a registered, certified account before any file can be downloaded (S20). The published routes of DEAP, AMIGOS and MAHNOB-HCI did not function at the re-check of 2026-09-11 (Section S20.1(c)); the rule rates access gating and states no separate level for a route found non-functional, so the three are rated Moderate. D4 Low for fully open CC-BY/CC0 with persistent DOI/accession and open formats (GAMEEMO, IESD, EmoEEG-MC, Daly), joined by LLaMAC, which returns data to an unauthenticated request under CC-BY 4.0; the Daly deposit states CC0 in its dataset description and CC BY 4.0 in its README, and both statements fall within this condition. Emognition (signed EULA returned to the data holder), K-EmoCon and Mixed Emotion (both approval-gated) are D4 Moderate on the same rule as the other gated corpora. DENS, although fully open on OpenNeuro, does not meet the Low condition, because its deposit carries two irreconcilable licence statements (CC0 in its dataset description, the Open Database License in its LICENSE file; Section S17), and is D4 Moderate. Voice-UI EEG and ECSMP, although fully open, do not meet it either, because their EEG is distributed only as MATLAB .mat files, the proprietary format that the D4 definition above sets against open formats, and both are D4 Moderate. DREAMER and EVA-MED are also gated and are D4 Moderate on the same rule: DREAMER releases its files only on approval of a Zenodo access request (Section S13), and EVA-MED, which the access check reclassified from fully open to conditional, is restricted-access with no licence stated (Section S20.1(c) and (d)).

Applicability Moderate flags reduced fit to the spatial-emotion question: cross-cultural SEED-GER/FRA (n = 8 each) and SEED-MYA (n = 20), consumer-grade DREAMER/AMIGOS/GAMEEMO, conversational EAV, Voice-UI, imagined-emotion IESD (imagined ≠ environment-evoked), stress-paradigm EVA-MED. Applicability Low for film/music/video-elicited dimensional or rich-discrete corpora whose V-A-D substrate transfers directly to VR-walkthrough / MoBI affect decoding (DEAP, SEED, SEED-IV, SEED-V, SEED-VII, SEED-SD, MPED, MAHNOB-HCI, FACED, DENS, EmoEEG-MC, Daly), together with Mixed Emotion, which pairs a full V-A-D substrate with peripheral channels. Applicability is Moderate for K-EmoCon (conversational), ECSMP (sleep and cognition design), Emognition (consumer Muse 2 headband) and LLaMAC (consumer-grade sensors). Where a corpus meets both descriptions, the reduced-fit reason governs, as for the film-elicited but consumer-grade DREAMER, AMIGOS and Emognition. EmoEEG-MC, which interleaves video-elicited and imagery trials, and SEED-SD, which presents film clips under three sleep conditions, are Low, because the nearest reduced-fit reasons, imagined emotion (IESD) and a sleep and cognition design (ECSMP), each describe only part of these two designs.

### S12.2 Compliance summary

**Number (percentage) of datasets at each risk level, by domain (n = 27):**

| **Domain** | **Low** | **Moderate** | **High** |
| --- | --- | --- | --- |
| D1: Dataset construction | 18 (66.7%) | 9 (33.3%) | 0 (0.0%) |
| D2: Annotation process | 20 (74.1%) | 7 (25.9%) | 0 (0.0%) |
| D3: Documentation / transparency | 11 (40.7%) | 16 (59.3%) | 0 (0.0%) |
| D4: Accessibility / reproducibility | 5 (18.5%) | 21 (77.8%) | 1 (3.7%) |
| Applicability concern | 13 (48.1%) | 14 (51.9%) | 0 (0.0%) |

Aggregate RoB profile:

Datasets rated Low across all four domains: 2/27 = 7.4% (EmoEEG-MC, OpenNeuro Film Clips/Daly, both fully open BIDS/CC corpora with peer-reviewed data descriptors).

Datasets with no High-risk domain: 26/27 = 96.3%.

Datasets with ≥ 1 High-risk domain: 1/27 = 3.7% (MPED, D4 accessibility).

Of the 108 (dataset × 4-domain) appraisal cells, 54 are Low-risk (50.0% of domain judgements); only 1 cell (0.9%) is High-risk, with the remaining 53 cells (49.1%) Moderate. The compliance gradient is strongest at annotation (74.1% Low) and construction (66.7% Low) and weakest at accessibility / reproducibility (18.5% Low, the single High), and it is not monotonic across the pipeline, since annotation sits above construction, while documentation (40.7% Low) falls between construction and accessibility, indicating that the dominant residual bias in published EEG emotion corpora is access gating and proprietary-format / missing-datasheet documentation, not flawed signal acquisition or annotation. This mirrors the openness (FAIR) finding of RQ1 (manuscript Section 3.2): EEG emotion datasets are technically well-constructed but under-documented and access-gated relative to FAIR best practice.

## S13 Per-Dataset Eligibility Assessment Against Criteria (a) to (e), by Type Stratum

Back-reference: manuscript Section 2.3 (Eligibility and risk of bias); summary in Supplementary Section S0.

Each of the 27 included datasets is shown against the five eligibility criteria that operationalise the single public-obtainability inclusion rule plus the EEG-specific precondition gate. All 27 satisfy every criterion (✓); excluded datasets fail at least one and are recorded in Supplementary Section S6 with their EX-code (never enumerated here). Entries 23 to 27 were identified by the second search pass (2026-09-10) and were assessed against the same five criteria; criterion (d) was evaluated for each of them on 2026-09-11 under the two-route rule stated in Section S0.

Eligibility criteria:

(a) Scalp EEG signal present: the resource records scalp EEG (precondition gate; failures = EX-1 no EEG / EX-2 non-EEG neuroimaging).

(b) Emotion / affect label present: discrete emotion categories or dimensional affect ratings (for example valence, arousal and dominance); failures = EX-3 no emotion label.

(c) Verifiable persistent documentation: a peer-reviewed paper, archived datasheet, or repository/registry record with a persistent identifier (DOI / OpenNeuro accession / Zenodo / Mendeley).

(d) Universal accessibility regardless of nationality: open download or a subscription/registration/DUA pathway any researcher worldwide can satisfy; nationality- or geography-gated access fails (EX-4).

(e) Distinct verified resource: a single, existing, non-duplicate corpus (not a re-label of another entry; not an unverified or non-existent name).

Type strata: Dimensional-VAD, Discrete multi-emotion, Multimodal, Continuous-naturalistic, Wearable-field, Dialogue-social (the task clusters of manuscript Table 1, defined in its footnote a).

Datasets 1 to 20 are grouped by type stratum in the order listed above, in ascending ID order within each stratum; datasets 21 to 27 follow in ascending ID order, with their stratum given in the Type stratum column.

| **ID** | **Dataset** | **Type stratum** | **(a) EEG** | **(b) Emotion label** | **(c) Persistent doc** | **(d) Universal access** | **(e) Distinct** |
| --- | --- | --- | --- | --- | --- | --- | --- |
| 1 | DEAP | Dimensional-VAD | ✓ | ✓ V-A-D + liking/familiarity | ✓ DOI 10.1109/T-AFFC.2011.15 | ✓ signed EULA (universal) | ✓ |
| 8 | DREAMER | Dimensional-VAD | ✓ | ✓ V-A-D (5-pt SAM) | ✓ DOI 10.1109/JBHI.2017.2688239 + Zenodo record 546113 | ✓ Zenodo access request (universal) | ✓ |
| 17 | EVA-MED | Dimensional-VAD | ✓ | ✓ V/A enhanced + stress | ✓ DOI 10.1038/s41597-025-06214-y | ✓ Conditional (restricted ScienceDB deposit, universal) | ✓ |
| 2 | SEED | Discrete multi-emotion | ✓ | ✓ 3-class (pos/neu/neg) | ✓ DOI 10.1109/TAMD.2015.2431497 | ✓ BCMI EULA (universal) | ✓ |
| 3 | SEED-IV | Discrete multi-emotion | ✓ | ✓ 4-class | ✓ DOI 10.1109/TCYB.2018.2797176 | ✓ BCMI EULA (universal) | ✓ |
| 4 | SEED-V | Discrete multi-emotion | ✓ | ✓ 5-class | ✓ DOI 10.1109/TCDS.2021.3071170 | ✓ BCMI EULA (universal) | ✓ |
| 5 | SEED-VII | Discrete multi-emotion | ✓ | ✓ 7-state + continuous intensity | ✓ DOI 10.1109/TAFFC.2024.3485057 | ✓ BCMI request (universal) | ✓ |
| 6 | SEED-GER | Discrete multi-emotion | ✓ | ✓ 3-class | ✓ DOI 10.1088/1741-2552/ac5c8d | ✓ BCMI EULA (universal) | ✓ |
| 7 | SEED-FRA | Discrete multi-emotion | ✓ | ✓ 3-class | ✓ DOI 10.1088/1741-2552/ac5c8d | ✓ BCMI EULA (universal) | ✓ |
| 11 | FACED | Discrete multi-emotion | ✓ | ✓ 9-class + dimensional | ✓ DOI 10.1038/s41597-023-02650-w | ✓ registration (Synapse syn50614194, certified account, universal) | ✓ |
| 12 | MPED | Discrete multi-emotion | ✓ | ✓ 7-class | ✓ DOI 10.1109/ACCESS.2019.2891579 | ✓ research agreement (GitHub request, universal) | ✓ |
| 15 | IESD (ds003004) | Discrete multi-emotion | ✓ | ✓ 15 imagined states | ✓ DOI 10.18112/openneuro.ds003004.v1.1.1 | ✓ FullyOpen (CC0 / OpenNeuro) | ✓ |
| 9 | AMIGOS | Multimodal | ✓ | ✓ V-A + basic emotions, multi-rater | ✓ DOI 10.1109/TAFFC.2018.2884461 | ✓ research agreement (universal) | ✓ |
| 10 | MAHNOB-HCI | Multimodal | ✓ | ✓ V-A-D + keywords | ✓ DOI 10.1109/T-AFFC.2011.25 | ✓ registration (universal) | ✓ |
| 16 | EmoEEG-MC (ds005540) | Multimodal | ✓ | ✓ 7-class + dimensional | ✓ DOI 10.1038/s41597-025-05349-2 | ✓ FullyOpen (OpenNeuro BIDS) | ✓ |
| 14 | DENS | Continuous-naturalistic | ✓ | ✓ V-A-D per clip + discrete choices on localised events | ✓ DOI 10.1101/2021.08.04.455041 | ✓ FullyOpen (OpenNeuro ds003751) | ✓ |
| 20 | OpenNeuro Film Clips / Daly (ds002721) | Continuous-naturalistic | ✓ | ✓ 8 induced-emotion ratings (9-point scale) | ✓ DOI 10.1038/s41597-020-0507-6 | ✓ FullyOpen (CC0 / OpenNeuro) | ✓ |
| 13 | GAMEEMO | Wearable-field | ✓ | ✓ 4 game conditions + SAM valence/arousal | ✓ DOI 10.17632/b3pn4kwpmn.3 | ✓ FullyOpen (CC-BY 4.0 / Mendeley) | ✓ |
| 18 | EAV | Dialogue-social | ✓ | ✓ 5-class + self-rated V-A | ✓ DOI 10.5281/zenodo.10205702 | ✓ Conditional (CC-BY-4.0 + DUA, universal) | ✓ |
| 19 | Voice-UI EEG | Dialogue-social | ✓ | ✓ 9 adjective-pair ratings + 2 derived factors (stability, favourability) | ✓ DOI 10.1038/s41597-024-03887-9 | ✓ FullyOpen (CC0 / figshare) | ✓ |
| 21 | SEED-MYA | Discrete multi-emotion | ✓ 62-ch scalp EEG | ✓ 3-class (positive/neutral/negative) | ✓ DOI 10.1109/TAFFC.2025.3586444 + EMBC 2024 companion | ✓ BCMI request (universal) | ✓ |
| 22 | SEED-SD | Discrete multi-emotion | ✓ 62-ch scalp EEG | ✓ 4-class + intensity 1-5 | ✓ DOI 10.1109/TAFFC.2025.3572504 | ✓ BCMI request (universal) | ✓ |
| 23 | Emognition | Wearable-field | ✓ | ✓ 9 discrete emotions + SAM valence/arousal/motivation | ✓ DOI 10.1038/s41597-022-01262-0 | ✓ signed EULA (Harvard Dataverse, universal) | ✓ |
| 24 | K-EmoCon | Dialogue-social | ✓ | ✓ arousal/valence + categorical, multi-rater | ✓ DOI 10.1038/s41597-020-00630-y | ✓ Conditional (Zenodo request + user agreement, universal) | ✓ |
| 25 | ECSMP | Multimodal | ✓ | ✓ 6-class + self-rated intensity | ✓ DOI 10.1016/j.dib.2021.107660 | ✓ FullyOpen (CC-BY 4.0 / Mendeley) | ✓ |
| 26 | LLaMAC | Multimodal | ✓ | ✓ V-A-D + 5-class with intensity | ✓ DOI 10.1038/s41597-025-06165-4 | ✓ FullyOpen (CC-BY 4.0 / figshare) | ✓ |
| 27 | Mixed Emotion (Tsinghua) | Multimodal | ✓ | ✓ V-A-D + PANAS + positive/negative/mixed | ✓ DOI 10.1038/s41597-024-03676-4 | ✓ Conditional (Zenodo request + DUA, universal) | ✓ |

All 27 datasets satisfy criteria (a) to (e). Borderline cases are noted for transparency. EAV passes (d) as Conditional (CC-BY-4.0 with a universally completable DUA), and EVA-MED (id 17) is also classed Conditional, because its deposit reverted to restricted access, as recorded by the identifier audit (Section S20); neither classification disturbs eligibility, which turns on universal grantability rather than on class. Voice-UI EEG (id 19) satisfies all five criteria; its descriptor reports 63 EEG channels acquired at 1,000 Hz, of which 57 are retained in the preprocessed release, and no montage coordinate file is deposited, which affects montage-related scoring rather than eligibility. DEAP (id 1), AMIGOS (id 9) and MAHNOB-HCI (id 10) satisfied (d) at corpus assembly but their access routes did not respond at the access re-check of 2026-09-11; they are retained under the time-dimension rule of Section S0 and flagged on the access_status axis rather than re-judged on (d).

## S14 AI-Readiness Criterion Weighting and Weight-Sensitivity Analysis

Back-reference: manuscript Section 2.4.2 (AI-readiness) and Table 2 (criteria and applied weights); Section 3.5 (weight sensitivity and corpus composition), whose details are in Sections S14.1 and S14.3.

Rationale for equal weighting. The composite AI-readiness score is the unweighted mean of the three criteria, AIR = (AS + TAS + OMS) / 3, so each criterion carries a weight of exactly one third. The weight is the exact fraction, not the rounded decimal 0.333 used for display in the tables: three weights of 0.333 sum to 0.999, so a composite formed from them does not equal the mean of the sub-scores. Equal weighting is a deliberate no-preference default rather than an estimated quantity, and this review makes no claim that it is optimal. Its justification is negative: no published evidence establishes a defensible priority among annotation richness, task breadth and label structure for a general-purpose readiness index, and different communities would in any case weight them differently. Weighting the criteria equally therefore avoids asserting a priority the evidence does not support. Each of the three sub-scores that enter the composite is computed by executable code from the atomic criterion states coded from the canonical attribute table under the deterministic codebook (Section S24), so the weighting is the only discretionary element in the composite, and the robustness question is confined to it. Because equal weighting is a choice rather than an estimate, the relevant robustness question is whether the ordering survives alternative weights, which S14.1 answers directly.

Method. Robustness is assessed by perturbing the equal weights by ±0.10 on one criterion at a time and offsetting the change by ∓0.05 across the other two, which yields six alternative schemes alongside the primary equal-weight scheme. For each scheme the 27 datasets are re-scored and re-ranked, and two quantities are reported: the Spearman rank correlation of the perturbed ordering against the primary ordering, and the number of discordant dataset pairs out of the 351 possible pairs. Two conventions are fixed. Weights are exact fractions rather than printed decimals, and rank statistics are computed on unrounded composites, since rounding to two decimal places creates ties that understate discordance. The composite is reported as a continuous score, so the quantity of interest is the stability of the rank ordering.

### S14.1 Weight-sensitivity analysis (±0.10 perturbation of the equal weights)

| **Scheme** | **w(AS)** | **w(TAS)** | **w(OMS)** | **Spearman ρ vs primary** | **Discordant pairs (of 351)** | **Concordance** |
| --- | --- | --- | --- | --- | --- | --- |
| Equal weights (primary) | 0.333 | 0.333 | 0.333 | 1.000 | 0 | 100.0% |
| TAS +0.10 | 0.283 | 0.433 | 0.283 | 0.992 | 7 | 98.0% |
| AS +0.10 | 0.433 | 0.283 | 0.283 | 0.995 | 5 | 98.6% |
| OMS +0.10 | 0.283 | 0.283 | 0.433 | 0.985 | 12 | 96.6% |
| TAS −0.10 | 0.383 | 0.233 | 0.383 | 0.982 | 13 | 96.3% |
| AS −0.10 | 0.233 | 0.383 | 0.383 | 0.996 | 4 | 98.9% |
| OMS −0.10 | 0.383 | 0.383 | 0.233 | 0.991 | 8 | 97.7% |

The rank ordering is the robust result: Spearman ρ is at least 0.982 against the primary ordering under every perturbation, and the worst case is 13 discordant pairs of 351, which is 96.3% concordance. The worst case arises when task alignment is down-weighted (TAS −0.10), and the next largest disturbance, 12 pairs, when ontology maturity is up-weighted (OMS +0.10), which is what would be expected of a corpus whose sharpest differentiation lies on the ontology axis.

### S14.2 Applied weights

| **Criterion** | **Applied weight** |
| --- | --- |
| TAS (Task Alignment Score) | 1/3 exact |
| AS (Annotation Specificity) | 1/3 exact |
| OMS (Ontology-Maturity Sub-score) | 1/3 exact |

The applied weights give the composite AI-readiness score AIR = (AS + TAS + OMS) / 3 on the 1.0–4.0 scale. Across the corpus this yields a mean AIR of 2.684 and a median of 2.60, with a range of 2.10 to 3.33 (Supplementary Table S18).
 The composite is reported as a continuous score, together with its three sub-scores and the resulting rank ordering. The arithmetic ceiling of the codebook is 4.00, since each axis reaches 4.0 at its raw maximum, and the highest composite in the corpus (3.33) lies well below it. The five top states that no corpus attains all record one and the same absence, that of a controlled vocabulary or a formal ontology, which is the L4 = L5 = 0 finding this review reports (Section S19). These states keep their specified maxima, because lowering them to bring the ceiling within reach of the corpus would remove that finding from the scores.

### S14.3 Corpus-composition sensitivity

The SEED line contributes 8 of the 27 appraised corpora, recorded in one laboratory under one protocol on one 62-channel montage, so the 27 corpora are not fully independent units. Collapsing the eight SEED variants into a single averaged unit leaves 20 units. The AI-readiness mean then moves from 2.684 to 2.77 and the median from 2.60 to 2.785, because the SEED variants score below the corpus mean (family mean 2.44). The correlation between ontology level and AI-readiness falls from ρ = 0.68 to ρ = 0.56. No reported conclusion reverses. The collapsed corpus stays concentrated at Level 3 (16 of the 20 units, the other 4 at Level 2), no unit reaches Level 4 or Level 5, and every association keeps its direction. The 27-corpus enumeration remains the primary analysis, because each SEED variant is distributed as a separate corpus (Section S24.1, Unit of analysis). The effective number of independent units is nevertheless nearer 20, and the correlations should be read against that count. The script scripts/score_engine.py in the reproducibility package reproduces these values (output field seed_collapse).

## S15 Per-Dataset AI-Readiness Scorecard, Ordered by Composite

Back-reference: manuscript Section 3.3 (AI-readiness, RQ2) and Figure 7.

AIR = (AS + TAS + OMS) / 3 with weights fixed at exactly one third each; scale 1.0 to 4.0. Corpus mean 2.684, median 2.60, range 2.10 to 3.33. The composite is reported as a continuous score, with its three sub-scores and the resulting rank ordering (manuscript Section 2.4.2). Rank is the mean rank: tied composites share the mean of the positions they occupy and are listed in ascending dataset-ID order.

### S15.1 All 27 datasets ordered by AI-readiness composite

| **Rank** | **ID** | **Dataset** | **AS** | **TAS** | **OMS** | **AIR** |
| --- | --- | --- | --- | --- | --- | --- |
| 1 | 16 | EmoEEG-MC (ds005540) | 3.2 | 3.2 | 3.6 | 3.33 |
| 2 | 9 | AMIGOS | 4.0 | 3.2 | 2.5 | 3.23 |
| 3.5 | 14 | DENS | 3.0 | 3.2 | 3.2 | 3.13 |
| 3.5 | 24 | K-EmoCon | 3.9 | 3.0 | 2.5 | 3.13 |
| 5 | 11 | FACED | 2.8 | 3.5 | 2.9 | 3.07 |
| 6.5 | 1 | DEAP | 3.1 | 3.2 | 2.7 | 3.00 |
| 6.5 | 10 | MAHNOB-HCI | 3.1 | 3.2 | 2.7 | 3.00 |
| 8 | 5 | SEED-VII | 3.2 | 3.0 | 2.7 | 2.97 |
| 9 | 27 | Mixed Emotion (Tsinghua) | 3.2 | 3.0 | 2.5 | 2.90 |
| 10 | 8 | DREAMER | 3.0 | 3.2 | 2.3 | 2.83 |
| 11 | 20 | OpenNeuro Film Clips EEG (ds002721 / Daly Affective Music family) | 3.1 | 2.2 | 3.1 | 2.80 |
| 12 | 17 | EVA-MED (Enhanced Valence-Arousal Multimodal Emotion Dataset) | 2.7 | 2.5 | 3.1 | 2.77 |
| 13 | 26 | LLaMAC | 3.2 | 2.5 | 2.5 | 2.73 |
| 14.5 | 4 | SEED-V | 2.8 | 3.2 | 1.8 | 2.60 |
| 14.5 | 19 | Voice-UI EEG Emotion Dataset | 3.1 | 2.2 | 2.5 | 2.60 |
| 16 | 22 | SEED-SD | 3.0 | 2.8 | 1.9 | 2.57 |
| 17 | 12 | MPED | 2.7 | 3.0 | 1.9 | 2.53 |
| 18.5 | 3 | SEED-IV | 2.8 | 2.8 | 1.9 | 2.50 |
| 18.5 | 13 | GAMEEMO | 3.0 | 2.0 | 2.5 | 2.50 |
| 20.5 | 2 | SEED | 2.5 | 3.0 | 1.8 | 2.43 |
| 20.5 | 23 | Emognition | 3.0 | 2.0 | 2.3 | 2.43 |
| 23 | 15 | Imagined Emotion Study (IESD, ds003004) | 2.7 | 1.8 | 2.5 | 2.33 |
| 23 | 18 | EAV (EEG-Audio-Video) | 2.6 | 2.5 | 1.9 | 2.33 |
| 23 | 25 | ECSMP | 2.7 | 2.0 | 2.3 | 2.33 |
| 25 | 7 | SEED-FRA | 2.7 | 2.5 | 1.4 | 2.20 |
| 26 | 6 | SEED-GER | 2.5 | 2.5 | 1.4 | 2.13 |
| 27 | 21 | SEED-MYA | 2.5 | 2.2 | 1.6 | 2.10 |

## S16 Overview of All 27 Datasets

Back-reference: manuscript Section 3.1 (The appraised corpus) and Table 1, which condenses this table; per-dataset identification stream and provenance in Supplementary Section S5. Each cell other than Year summarises the source-verified attribute record for that dataset; the full verbatim evidence, with its source and, where one was recorded, its check date, is in data/attributes.json of the reproducibility package (Reproducibility_package.zip). The Year column records the publication year of the corpus descriptor record, that is, the dated reference by which the corpus is cited; it is not the repository release year, and for corpora deposited after the original acquisition the two differ (IESD is the clearest case, carrying a 2009 acquisition lineage but a 2022 descriptor record, and the Year column takes the descriptor record). The Year values are those of the corpus record (data/corpus.json), which the adoption ledger of Section S22 also uses; the separate year field of data/attributes.json differs for seven datasets (IDs 1, 4, 5, 8, 10, 14 and 15) and gives 2009 for IESD. The Access column gives the operational class label of the scored ledger. FullyOpen is fully open (anonymous self-service retrieval). Academic is registration- or licence-gated: retrieval needs a free account, an institutional approval or an end-user licence agreement, and any eligible researcher can obtain it irrespective of nationality or region. Conditional requires a data-use agreement or a discretionary approval decision. No dataset is closed, that is, without a universally grantable route. Where an end-user licence agreement (EULA) is named, it identifies the licence instrument in use, not a separate access class.

| **ID** | **Dataset** | **Year** | **Subjects** | **EEG channels** | **Emotion label** | **Modalities** | **Access** |
| --- | --- | --- | --- | --- | --- | --- | --- |
| 1 | DEAP | 2011 | 32 (22 with frontal face video) | 32, BioSemi ActiveTwo, active Ag/AgCl electrodes, 10-20 placement | Dimensional: valence, arousal, dominance and liking (continuous 1 to 9) per trial, plus familiarity (1 to 5); no discrete classes | EEG plus eight peripheral channels (EOG, EMG, GSR, respiration, plethysmograph, skin temperature), face video and self-report | Academic |
| 2 | SEED | 2015 | 15 (7 male, 8 female); 12 with eye-movement data | 62, ESI NeuroScan, international 10-20 system | Three discrete classes (positive, neutral, negative), stimulus-defined per film clip | EEG and eye movements (SMI eye-tracking glasses), plus derived EEG features | Academic |
| 3 | SEED-IV | 2018 | 15 | 62, ESI NeuroScan | Four discrete classes (happy, sad, fear, neutral), one per trial; no dimensional ratings | EEG and eye movements (SMI eye-tracking glasses), both released | Academic |
| 4 | SEED-V | 2022 | 16 (6 male, 10 female), three sessions each | 62, ESI NeuroScan | Five discrete classes (happy, sad, fear, disgust, neutral), one per film-clip trial | EEG and eye movements (SMI eye-tracking glasses); eye images recorded for a subset are not distributed | Academic |
| 5 | SEED-VII | 2024 | 20 (10 male, 10 female) | 62, ESI NeuroScan, active Ag/AgCl cap, 10-20 system | Seven discrete classes (six basic emotions plus neutral) with per-trial self-rated intensity and a continuous intensity trace | EEG, eye movements (Tobii Pro Fusion) and behavioural self-report | Academic |
| 6 | SEED-GER | 2022 | 8 German participants (7 male, 1 female) | 62, ESI NeuroScan | Three discrete classes (positive, neutral, negative); no dimensional ratings | EEG and eye movements (SMI eye-tracking glasses), with per-trial self-report | Academic |
| 7 | SEED-FRA | 2022 | 8 French participants (5 male, 3 female), three sessions each | 62, ESI NeuroScan | Three discrete classes (positive, neutral, negative) at film-clip level | EEG and eye movements (SMI eye-tracking glasses), with per-trial self-report | Academic |
| 8 | DREAMER | 2017 | 23 (14 male, 9 female) | 14, Emotiv EPOC wireless headset | Dimensional: valence, arousal and dominance (1 to 5, SAM) per film clip; no discrete classes | EEG and ECG (two channels) | Academic |
| 9 | AMIGOS | 2018 | 40 (short videos); 37 of them also in the long-videos experiment | 14, Emotiv EPOC wireless headset | Valence, arousal, dominance, liking and familiarity (1 to 9) plus basic-emotion selection; external valence and arousal annotation | EEG, ECG, GSR, accelerometry, head gyroscope, frontal face video and full-body RGB and depth video | Academic |
| 10 | MAHNOB-HCI | 2011 | 30 recorded, 27 analysed | 32, BioSemi Active II, active Ag/AgCl electrodes, 10-20 placement | Nine emotion keywords plus arousal, valence, dominance and predictability (1 to 9) per clip | EEG, ECG, GSR, respiration, skin temperature, six-camera face video, audio and eye gaze | Academic |
| 11 | FACED | 2023 | 123 (75 female); no exclusions | 32, international 10-20 system, wet Ag/AgCl electrodes (NeuSen.W32) | Nine discrete classes per clip (stimulus-defined) plus 12 self-report ratings per clip (0 to 7) | EEG only, with behavioural self-report | Academic |
| 12 | MPED | 2019 | 23 | 62, ESI NeuroScan, international 10-20 layout | Seven discrete classes (joy, funny, anger, fear, disgust, sadness, neutral) plus a resting-state code | EEG, GSR, respiration and ECG, with self-report ratings | Academic |
| 13 | GAMEEMO | 2020 | 28; no exclusions | 14, Emotiv EPOC+ wireless headset | Four game conditions (boring, calm, horror, funny) plus SAM self-report on scanned forms; no machine-readable label file | EEG only; game screen-capture videos document the stimuli | FullyOpen |
| 14 | DENS | 2021 | 43 recruited; 40 with EEG; 34 with self-assessment labels | 129 (EGI 128-electrode geodesic net, vertex reference) | Valence, arousal and dominance (1 to 9) per clip plus discrete emotion choices on localised events | EEG, ECG, EMG and behavioural self-report | FullyOpen |
| 15 | Imagined Emotion Study (IESD, ds003004) | 2022 | 34 in the deposit (32 analysed in the 2009 study) | Custom high-density BioSemi montage: 250 scalp electrodes acquired, 134 to 235 retained per subject | 15 discrete imagined emotion categories (8 positive, 7 negative), one per imagery period | EEG only | FullyOpen |
| 16 | EmoEEG-MC (ds005540) | 2025 | 63 recruited; 60 analysed and deposited | 64 wet electrodes, international 10-20 system (g.HIamp) | Seven discrete stimulus classes plus 10 self-ratings (0 to 7) covering six emotions, arousal, valence, familiarity and liking | EEG, GSR and PPG, with behavioural ratings and stimulus features | FullyOpen |
| 17 | EVA-MED (Enhanced Valence-Arousal Multimodal Emotion Dataset) | 2025 | 64 (33 male, 31 female) | 64, NeuroScan cap, 10-20 system; channel list inside the restricted deposit; EEG sampling rate not reported | Valence (four classes) and arousal (calm or stress) labels plus SAM valence and arousal ratings | EEG, EOG, ECG, wrist pulse interval, self-report and psychometric questionnaires | Conditional |
| 18 | EAV (EEG-Audio-Video) | 2024 | 42; no attrition | 30, Ag/AgCl, BrainAmp, 10-20 core with 10-10 extensions | Five discrete classes (neutral, anger, happiness, sadness, calmness) in listening and speaking tasks; supplementary self-rated valence and arousal | EEG, audio (speaking task), facial video and behavioural ratings | Conditional |
| 19 | Voice-UI EEG Emotion Dataset | 2024 | 50 recruited; 44 deposited; 38 in the classification benchmark | 63 acquired (actiCHamp, 10-10 system); 57 in the preprocessed release | Nine adjective-pair ratings (-3 to +3) per trial, two derived factor scores (stability, favourability) and a four-level stimulus condition | EEG, ECG, PPG, GSR and facial video, with a per-trial questionnaire | FullyOpen |
| 20 | OpenNeuro Film Clips EEG (ds002721 / Daly Affective Music family) | 2020 | 31 (18 female), aged 18 to 66 | 19, international 10-20 system, FCz reference | Eight induced-emotion ratings per trial (9-point scale), non-exclusive | EEG only; self-reported ratings are embedded as trigger codes | FullyOpen |
| 21 | SEED-MYA | 2025 | 20 Myanmar participants (11 male, 9 female) | 62, ESI NeuroScan, international 10-20 system | Three discrete classes (positive, neutral, negative); no dimensional ratings | EEG and eye movements (Tobii Pro Lab screen-based eye tracker) | Academic |
| 22 | SEED-SD | 2025 | 40 (20 male, 20 female), three sleep-condition sessions each | 62, ESI NeuroScan wet cap | Four discrete classes (happiness, sadness, fear, neutral) with self-rated intensity (1 to 5), under three sleep conditions | EEG and eye movements (SMI glasses); ECG and EOG channels in the raw recording | Academic |
| 23 | Emognition | 2022 | 43 (21 female) | 4 dry electrodes (AF7, AF8, TP9, TP10), Muse 2 headband | Nine discrete emotion ratings (1 to 5) plus SAM valence, arousal and motivation (1 to 9) per film clip | EEG plus wristband and smartwatch physiology (BVP, EDA, skin temperature, heart rate, motion) and upper-body video | Academic |
| 24 | K-EmoCon | 2020 | 32 in 16 dyads | 1 (Fp1), NeuroSky MindWave; released as band powers | Arousal and valence plus categorical emotions every 5 s, from self, partner and five external raters | EEG band powers, wrist physiology (Empatica E4), ECG-derived heart rate, debate audio and video | Conditional |
| 25 | ECSMP | 2021 | 89 (87 with valid EEG) | 7 prefrontal channels, ear reference (Neuroscan SynAmps RT) | Six discrete emotions, stimulus-defined, plus self-rated intensity (1 to 7) for each | EEG, ECG (experiment and overnight sleep), wrist PPG, EDA, skin temperature and accelerometry | FullyOpen |
| 26 | LLaMAC | 2025 | 114 recruited; 108 valid | 14, Emotiv EPOC X consumer headset, 10-20 placement | Valence, arousal and dominance (1 to 5) plus five discrete classes with intensity (1 to 6), liking and familiarity | EEG, EDA, PPG, skin temperature and respiration | FullyOpen |
| 27 | Mixed Emotion (Tsinghua) | 2024 | 80 recruited; 73 released | 21, dry-electrode DSI-24, international 10-20 system | Valence, arousal and dominance (1 to 9), short PANAS, amusement and disgust ratings, plus a positive, negative or mixed stimulus category | EEG, GSR, PPG, frontal face video and self-report | Conditional |

Two-stream provenance: Stream A = 16 datasets (databases and registers); Stream B = 6 net-new (citation searching: MPED, GAMEEMO, EAV; data-portal browsing: IESD, EmoEEG-MC, OpenNeuro Film Clips/Daly). Together with the five corpora identified by the second search pass of the Stream A strings (2026-09-10), the total included is 27. For EVA-MED (id 17) the descriptor states 64 EEG channels but no EEG sampling rate, and the channel list is held inside the restricted deposit; for Voice-UI EEG (id 19) the descriptor reports 63 channels acquired at 1,000 Hz, of which 57 are retained in the preprocessed release.

## S17 FAIR Principle Scores for All 27 Datasets

Back-reference: manuscript Section 3.2 (Openness, RQ1). Supplementary Figure S1 (Section S25) displays the scores in the table below.

Scores 0.0 to 4.0 per principle; composite = mean(F, A, I, R). Corpus means: F = 3.311, A = 3.511, I = 2.044, R = 2.563 (composite 2.857). Access class, recorded on a separate axis, is fully open 8, registration- or licence-gated 15, conditional 4, closed 0. All values are produced by the deterministic codebook from the canonical attribute table (Supplementary Section S24). Access class is given per dataset in Supplementary Table S16 and access status in the table below, with the dated access checks behind both reported in Supplementary Section S20.1; three resources (DEAP, AMIGOS, MAHNOB-HCI) carry the status route-non-functional-at-recheck.

| **ID** | **Dataset** | **F** | **A** | **I** | **R** | **Composite** | **Access status** |
| --- | --- | --- | --- | --- | --- | --- | --- |
| 1 | DEAP | 2.6 | 1.6 | 2.0 | 2.8 | 2.250 | route-non-functional-at-recheck |
| 2 | SEED | 2.6 | 3.6 | 1.8 | 2.2 | 2.550 | route-live |
| 3 | SEED-IV | 2.6 | 3.6 | 2.0 | 2.8 | 2.750 | route-live |
| 4 | SEED-V | 2.6 | 3.6 | 1.8 | 2.2 | 2.550 | route-live |
| 5 | SEED-VII | 2.6 | 3.6 | 1.5 | 2.2 | 2.475 | route-live |
| 6 | SEED-GER | 2.0 | 3.6 | 1.2 | 2.2 | 2.250 | route-live |
| 7 | SEED-FRA | 2.0 | 3.6 | 1.2 | 2.2 | 2.250 | route-live |
| 8 | DREAMER | 3.4 | 3.6 | 2.0 | 1.0 | 2.500 | route-live |
| 9 | AMIGOS | 2.6 | 1.6 | 2.0 | 2.8 | 2.250 | route-non-functional-at-recheck |
| 10 | MAHNOB-HCI | 2.6 | 1.6 | 1.8 | 1.8 | 1.950 | route-non-functional-at-recheck |
| 11 | FACED | 4.0 | 4.0 | 1.5 | 1.5 | 2.750 | route-live |
| 12 | MPED | 2.6 | 4.0 | 2.0 | 2.8 | 2.850 | route-live |
| 13 | GAMEEMO | 4.0 | 4.0 | 1.5 | 3.2 | 3.175 | route-live |
| 14 | DENS | 4.0 | 4.0 | 3.5 | 3.0 | 3.625 | route-live |
| 15 | Imagined Emotion Study (IESD, ds003004) | 4.0 | 4.0 | 3.2 | 3.8 | 3.750 | route-live |
| 16 | EmoEEG-MC (ds005540) | 4.0 | 4.0 | 3.8 | 4.0 | 3.950 | route-live |
| 17 | EVA-MED (Enhanced Valence-Arousal Multimodal Emotion Dataset) | 4.0 | 3.6 | 2.0 | 1.0 | 2.650 | route-live |
| 18 | EAV (EEG-Audio-Video) | 4.0 | 3.6 | 1.8 | 2.0 | 2.850 | route-live |
| 19 | Voice-UI EEG Emotion Dataset | 4.0 | 4.0 | 1.5 | 3.2 | 3.175 | route-live |
| 20 | OpenNeuro Film Clips EEG (ds002721 / Daly Affective Music family) | 4.0 | 4.0 | 3.8 | 3.8 | 3.900 | route-live |
| 21 | SEED-MYA | 2.6 | 3.6 | 1.2 | 2.2 | 2.400 | route-live |
| 22 | SEED-SD | 2.6 | 3.6 | 1.5 | 2.2 | 2.475 | route-live |
| 23 | Emognition | 4.0 | 3.6 | 2.0 | 2.8 | 3.100 | route-live |
| 24 | K-EmoCon | 4.0 | 3.6 | 2.0 | 2.5 | 3.025 | route-live |
| 25 | ECSMP | 4.0 | 4.0 | 1.8 | 3.8 | 3.400 | route-live |
| 26 | LLaMAC | 4.0 | 4.0 | 2.0 | 3.2 | 3.300 | route-live |
| 27 | Mixed Emotion (Tsinghua) | 4.0 | 3.2 | 2.8 | 2.0 | 3.000 | route-live |

Interoperability (I = 2.044) is the weakest FAIR principle corpus-wide, driven by .mat/.csv-centric distribution; the four BIDS-EEG OpenNeuro corpora (IESD, EmoEEG-MC, Daly ds002721 and DENS ds003751), three of them under CC0 or a CC-type licence and DENS carrying conflicting CC0 and Open Database License statements, anchor the high-FAIR end with the four highest composites in the corpus (3.625 to 3.950), consistent with the access-gating diagnosis in S12.

## S18 AI-Readiness Scores for All 27 Datasets

Back-reference: manuscript Section 3.3 (AI-readiness, RQ2) and Figure 7. The table repeats the ordered scorecard of Supplementary Section S15 so that the FAIR, AI-readiness and ontology scores can be read together across Sections S17 to S19.

AIR = (AS + TAS + OMS) / 3 with weights fixed at exactly one third each; scale 1.0 to 4.0. Corpus mean 2.684, median 2.60, range 2.10 to 3.33. The composite is reported as a continuous score, with its three sub-scores and the resulting rank ordering (manuscript Section 2.4.2). Rank is the mean rank: tied composites share the mean of the positions they occupy and are listed in ascending dataset-ID order.

| **Rank** | **ID** | **Dataset** | **AS** | **TAS** | **OMS** | **AIR** |
| --- | --- | --- | --- | --- | --- | --- |
| 1 | 16 | EmoEEG-MC (ds005540) | 3.2 | 3.2 | 3.6 | 3.33 |
| 2 | 9 | AMIGOS | 4.0 | 3.2 | 2.5 | 3.23 |
| 3.5 | 14 | DENS | 3.0 | 3.2 | 3.2 | 3.13 |
| 3.5 | 24 | K-EmoCon | 3.9 | 3.0 | 2.5 | 3.13 |
| 5 | 11 | FACED | 2.8 | 3.5 | 2.9 | 3.07 |
| 6.5 | 1 | DEAP | 3.1 | 3.2 | 2.7 | 3.00 |
| 6.5 | 10 | MAHNOB-HCI | 3.1 | 3.2 | 2.7 | 3.00 |
| 8 | 5 | SEED-VII | 3.2 | 3.0 | 2.7 | 2.97 |
| 9 | 27 | Mixed Emotion (Tsinghua) | 3.2 | 3.0 | 2.5 | 2.90 |
| 10 | 8 | DREAMER | 3.0 | 3.2 | 2.3 | 2.83 |
| 11 | 20 | OpenNeuro Film Clips EEG (ds002721 / Daly Affective Music family) | 3.1 | 2.2 | 3.1 | 2.80 |
| 12 | 17 | EVA-MED (Enhanced Valence-Arousal Multimodal Emotion Dataset) | 2.7 | 2.5 | 3.1 | 2.77 |
| 13 | 26 | LLaMAC | 3.2 | 2.5 | 2.5 | 2.73 |
| 14.5 | 4 | SEED-V | 2.8 | 3.2 | 1.8 | 2.60 |
| 14.5 | 19 | Voice-UI EEG Emotion Dataset | 3.1 | 2.2 | 2.5 | 2.60 |
| 16 | 22 | SEED-SD | 3.0 | 2.8 | 1.9 | 2.57 |
| 17 | 12 | MPED | 2.7 | 3.0 | 1.9 | 2.53 |
| 18.5 | 3 | SEED-IV | 2.8 | 2.8 | 1.9 | 2.50 |
| 18.5 | 13 | GAMEEMO | 3.0 | 2.0 | 2.5 | 2.50 |
| 20.5 | 2 | SEED | 2.5 | 3.0 | 1.8 | 2.43 |
| 20.5 | 23 | Emognition | 3.0 | 2.0 | 2.3 | 2.43 |
| 23 | 15 | Imagined Emotion Study (IESD, ds003004) | 2.7 | 1.8 | 2.5 | 2.33 |
| 23 | 18 | EAV (EEG-Audio-Video) | 2.6 | 2.5 | 1.9 | 2.33 |
| 23 | 25 | ECSMP | 2.7 | 2.0 | 2.3 | 2.33 |
| 25 | 7 | SEED-FRA | 2.7 | 2.5 | 1.4 | 2.20 |
| 26 | 6 | SEED-GER | 2.5 | 2.5 | 1.4 | 2.13 |
| 27 | 21 | SEED-MYA | 2.5 | 2.2 | 1.6 | 2.10 |

## S19 OFI Scores and Upgrade Potential for All 27 Datasets

Back-reference: manuscript Section 3.4 (Ontology maturity, RQ3) and Figure 10. Supplementary Figure S2 (Section S25) displays the sub-indices in the table below, grouped by task cluster.

OFI = mean(SRS, RFS, Integration), each 1.0 to 4.0; corpus mean OFI 2.385. Corpus ontology-level distribution: L1 4 (14.8%), L2 7 (25.9%), L3 16 (59.3%), L4 0 (0.0%), L5 0 (0.0%). Cluster OFI means: continuous-naturalistic 3.233 (n = 2); wearable-field 3.050 (n = 2); multimodal 2.889 (n = 6); dimensional-VAD 2.556 (n = 3); dialogue-social 2.478 (n = 3); discrete-multi-emotion 1.764 (n = 11).

| **ID** | **Dataset** | **Level** | **SRS** | **RFS** | **Integration** | **OFI** | **Task cluster** |
| --- | --- | --- | --- | --- | --- | --- | --- |
| 1 | DEAP | L3 | 3.0 | 3.3 | 1.5 | 2.600 | dimensional-VAD |
| 2 | SEED | L1 | 1.0 | 1.0 | 1.5 | 1.167 | discrete-multi-emotion |
| 3 | SEED-IV | L2 | 2.0 | 1.3 | 1.5 | 1.600 | discrete-multi-emotion |
| 4 | SEED-V | L2 | 2.5 | 2.0 | 1.3 | 1.933 | discrete-multi-emotion |
| 5 | SEED-VII | L2 | 3.0 | 3.0 | 1.3 | 2.433 | discrete-multi-emotion |
| 6 | SEED-GER | L1 | 1.0 | 1.0 | 1.3 | 1.100 | discrete-multi-emotion |
| 7 | SEED-FRA | L1 | 1.0 | 1.0 | 1.3 | 1.100 | discrete-multi-emotion |
| 8 | DREAMER | L3 | 2.5 | 2.3 | 1.8 | 2.200 | dimensional-VAD |
| 9 | AMIGOS | L3 | 3.0 | 3.3 | 1.5 | 2.600 | multimodal |
| 10 | MAHNOB-HCI | L3 | 3.0 | 3.3 | 1.3 | 2.533 | multimodal |
| 11 | FACED | L3 | 3.5 | 3.3 | 1.8 | 2.867 | discrete-multi-emotion |
| 12 | MPED | L2 | 2.5 | 1.7 | 1.5 | 1.900 | discrete-multi-emotion |
| 13 | GAMEEMO | L3 | 3.5 | 3.0 | 2.4 | 2.967 | wearable-field |
| 14 | DENS | L3 | 3.5 | 3.3 | 2.9 | 3.233 | continuous-naturalistic |
| 15 | Imagined Emotion Study (IESD, ds003004) | L3 | 2.5 | 1.7 | 2.9 | 2.367 | discrete-multi-emotion |
| 16 | EmoEEG-MC (ds005540) | L3 | 3.5 | 3.3 | 3.5 | 3.433 | multimodal |
| 17 | EVA-MED (Enhanced Valence-Arousal Multimodal Emotion Dataset) | L3 | 3.0 | 3.0 | 2.6 | 2.867 | dimensional-VAD |
| 18 | EAV (EEG-Audio-Video) | L2 | 2.0 | 1.7 | 2.1 | 1.933 | dialogue-social |
| 19 | Voice-UI EEG Emotion Dataset | L3 | 2.5 | 3.0 | 2.4 | 2.633 | dialogue-social |
| 20 | OpenNeuro Film Clips EEG (ds002721 / Daly Affective Music family) | L3 | 3.5 | 2.7 | 3.5 | 3.233 | continuous-naturalistic |
| 21 | SEED-MYA | L1 | 1.0 | 1.0 | 1.3 | 1.100 | discrete-multi-emotion |
| 22 | SEED-SD | L2 | 2.5 | 1.7 | 1.3 | 1.833 | discrete-multi-emotion |
| 23 | Emognition | L3 | 3.5 | 3.3 | 2.6 | 3.133 | wearable-field |
| 24 | K-EmoCon | L3 | 3.5 | 3.0 | 2.1 | 2.867 | dialogue-social |
| 25 | ECSMP | L2 | 2.5 | 3.0 | 2.4 | 2.633 | multimodal |
| 26 | LLaMAC | L3 | 3.5 | 3.3 | 2.4 | 3.067 | multimodal |
| 27 | Mixed Emotion (Tsinghua) | L3 | 3.5 | 3.3 | 2.4 | 3.067 | multimodal |

EmoEEG-MC is the corpus closest to L4 (formal BIDS schema + cross-context design) but is scored L3, since a standard data schema is not a semantic model; the highest OFI value (3.433) is carried by EmoEEG-MC, with the fully open BIDS corpora DENS (ds003751) and Daly (ds002721) next, tied at 3.233, echoing the openness gradient in S17: ontological formalisation is currently bottlenecked by Integration (interoperability/linkage), not by the richness of the emotion representations themselves.

Cluster OFI means, by member. The six task clusters are the descriptive labels of manuscript Table 1, whose footnote a defines each cluster by its members; the labels enter no score. Continuous-naturalistic: 3.233 (n = 2; DENS, Daly (ds002721)). Wearable-field: 3.050 (n = 2; GAMEEMO, Emognition). Multimodal: 2.889 (n = 6; AMIGOS, MAHNOB-HCI, EmoEEG-MC, ECSMP, LLaMAC, Mixed Emotion). Dimensional-VAD: 2.556 (n = 3; DEAP, DREAMER, EVA-MED). Dialogue-social: 2.478 (n = 3; EAV, Voice-UI, K-EmoCon). Discrete-multi-emotion: 1.764 (n = 11; SEED, SEED-IV, SEED-V, SEED-VII, SEED-GER, SEED-FRA, SEED-MYA, SEED-SD, MPED, FACED, IESD). The means are descriptive only. The clusters hold 2 to 11 datasets and their OFI ranges overlap: multimodal runs from 2.533 to 3.433, spanning the means of the two clusters placed above it, and discrete-multi-emotion from 1.100 to 2.867. The means therefore do not form a separable ranking. Both wearable-field datasets reach SRS 3.5 on consumer headsets of 14 channels (GAMEEMO) and 4 channels (Emognition), so a sparse montage does not entail a thin emotion representation. Within discrete-multi-emotion, the four flat SEED variants lie at 1.100 to 1.167, while FACED (2.867), SEED-VII (2.433) and IESD (2.367) lie well above the cluster mean. That mean therefore describes the composition of the cluster, not a property of its task type. Supplementary Figure S2 (Section S25) shows the sub-indices grouped by these clusters.

### Integrity notes (Sections S11–S19)

All 27 scored datasets are the verified included-corpus entries; the 11 excluded or held datasets (CASE, WESAD, DECAF, VREED, MVRS, NeuroEmo, SEED-VIG, SEED-VLA/VRW, SEED-DV, SEED-Multimodal/MULTI-SEED, EEGEMO) are not scored and do not enter any cell of Sections S11 to S19. Their disposition codes are recorded in Supplementary Section S6; the canonical excluded count is 11.

The three AI-readiness criteria are weighted equally (S14), which is a no-preference default rather than an estimated quantity, and no claim of an evidenced criterion priority is made. Weight-insensitivity is reported as the robustness property of the rank ordering (S14.1).

Every statistic below is computed from the attribute table as corrected by the identifier audit (S20), is recomputed from the data files of the reproducibility package (Reproducibility_package.zip), and is stated identically in the manuscript (including its figure legends), this supplement and the transparency workbook: N = 27; FAIR means F 3.311 / A 3.511 / I 2.044 / R 2.563 (composite 2.857); AIR mean 2.684 / median 2.60 / range 2.10 to 3.33, reported as a continuous score and rank ordering; criterion weights exactly one third each; ontology levels L1 4 / L2 7 / L3 16 / L4 0 / L5 0; access class fully open 8 / gated 15 / conditional 4 / closed 0. The script scripts/verify_docs.py in the reproducibility package recomputes these statistics from its data/ directory and checks that they appear in the manuscript, this supplement and the transparency workbook.

# Supplementary Material, Sections S20–S21

Sections S20 and S21 report the cross-verification stage specified in manuscript Section 2.5 and reported in manuscript Sections 3.1, 3.2 and 4.4. S20 is the persistent-identifier resolution ledger; S21 is the external coverage reconciliation against a separately built acquisition-level landscape.

## S20 Persistent-Identifier Resolution Ledger (Crossref / DataCite / OpenAlex)

Scope: every persistent identifier associated with one of the 27 appraised corpora, comprising each corpus descriptor record and, where one exists, its dataset-level deposit. Methodological references and excluded or held resources lie outside the scope of this ledger and are not listed or counted in it. Each identifier was submitted to three independent public metadata registries. An identifier counts as verified when it resolves in at least two of the three and the returned title matches the recorded title. Registry responses were also compared against one another. The identifiers were checked on two dates: on 1 August 2026, the 32 identifiers of the 22 corpora identified at the first search pass; and on 12 September 2026, the ten identifiers of the five corpora added at the second search pass (Section S20.1(f)). The access routes of all 27 corpora were re-tested separately on 11 September 2026 (Section S20.1(c)). The audited scope is 42 identifiers: 27 descriptor-level records (one for each corpus, except that SEED-GER and SEED-FRA share one descriptor DOI, IESD is represented by its OpenNeuro accession and SEED-MYA also carries a companion conference record), 12 further dataset-level deposits, and three supplement-only deposits (rows labelled SUPP-ONLY DOI, one of which, ds005540, is the dataset-level deposit of EmoEEG-MC), with methodological references and excluded or held resources outside the scope as stated above. Of those 42, 41 resolve in at least two of the three registries with a matching title; no identifier fails to resolve, and no two registries return conflicting titles for the same identifier. The single exception is the K-EmoCon Zenodo deposit (10.5281/zenodo.3931963), which resolves in DataCite with a matching title but is not indexed in Crossref or OpenAlex; it is reported as verified-in-one-registry rather than as a failure, because non-indexing of a repository deposit by a citation registry is a property of the registry rather than of the deposit. The table below lists the 32 identifiers checked on 1 August 2026, and the ten checked on 12 September 2026 are listed below it. In the Registries column, Cr denotes Crossref, Da DataCite and Op OpenAlex; in the Title check column, n/a marks a dataset-level deposit for which no separate title had been recorded, and the returned title is shown for comparison with the corpus name. The full ledger, with complete registry titles and containers, is also published in the transparency workbook (sheet S20_DOI_verification).

| **Role / label (truncated)** | **Identifier** | **Registries** | **Year** | **Registry title (truncated)** | **Title check** |
| --- | --- | --- | --- | --- | --- |
| DATASET 1: DEAP \| REF: DEAP: A Database for Emotion Analys | 10.1109/T-AFFC.2011.15 | Cr/Op | 2012 | DEAP: A Database for Emotion Analysis ;Using Physiological Sig | match |
| DATASET 2: SEED \| REF: Investigating Critical Frequency Ba | 10.1109/TAMD.2015.2431497 | Cr/Op | 2015 | Investigating Critical Frequency Bands and Channels for EEG-Ba | match |
| DATASET 3: SEED-IV \| REF: EmotionMeter: A Multimodal Frame | 10.1109/TCYB.2018.2797176 | Cr/Op | 2019 | EmotionMeter: A Multimodal Framework for Recognizing Human Emo | match |
| DATASET 4: SEED-V \| REF: Comparing Recognition Performance | 10.1109/TCDS.2021.3071170 | Cr/Op | 2022 | Comparing Recognition Performance and Robustness of Multimodal | match |
| DATASET 5: SEED-VII \| REF: SEED-VII: A Multimodal Dataset | 10.1109/TAFFC.2024.3485057 | Cr/Op | 2025 | SEED-VII: A Multimodal Dataset of Six Basic Emotions With Cont | match |
| DATASET 7: SEED-FRA \| DATASET 6: SEED-GER \| REF: Identifyi | 10.1088/1741-2552/ac5c8d | Cr/Op | 2022 | Identifying similarities and differences in emotion recognitio | match |
| DATASET 8: DREAMER \| REF: DREAMER: A Database for Emotion | 10.1109/JBHI.2017.2688239 | Cr/Op | 2018 | DREAMER: A Database for Emotion Recognition Through EEG and EC | match |
| DATASET 9: AMIGOS \| REF: AMIGOS: A Dataset for Affect, Per | 10.1109/TAFFC.2018.2884461 | Cr/Op | 2021 | AMIGOS: A Dataset for Affect, Personality and Mood Research on | match |
| DATASET 10: MAHNOB-HCI \| REF: A Multimodal Database for Af | 10.1109/T-AFFC.2011.25 | Cr/Op | 2012 | A Multimodal Database for Affect Recognition and Implicit Tagg | match |
| DATASET 11: FACED \| REF: A Large Finer-grained Affective C | 10.1038/s41597-023-02650-w | Cr/Op | 2023 | A Large Finer-grained Affective Computing EEG Dataset | match |
| DATASET 12: MPED \| REF: MPED: A Multi-Modal Physiological | 10.1109/ACCESS.2019.2891579 | Cr/Op | 2019 | MPED: A Multi-Modal Physiological Emotion Database for Discret | match |
| DATASET 13: GAMEEMO \| REF: Database for an emotion recognition system based on E | 10.1016/j.bspc.2020.101951 | Cr/Op | 2020 | Database for an emotion recognition system based on EEG signal | match |
| DATASET 14: DENS \| REF: Dataset on Emotion with Naturalist | 10.1101/2021.08.04.455041 | Cr/Op | 2021 | Dataset on Emotion with Naturalistic Stimuli (DENS) on Indian | match |
| DATASET 15: Imagined Emotion Study (IESD, ds003004) \| REF: | 10.18112/openneuro.ds003004.v1.1.1 | Da/Op | 2022 | Imagined Emotion Study | match |
| DATASET 16: EmoEEG-MC (ds005540) \| REF: A Multi-Context Em | 10.1038/s41597-025-05349-2 | Cr/Op | 2025 | A Multi-Context Emotional EEG Dataset for Cross-Context Emotio | match |
| DATASET 17: EVA-MED (Enhanced Valence-Arousal Multimodal E | 10.1038/s41597-025-06214-y | Cr/Op | 2025 | An Enhanced Valence-Arousal Multimodal Emotion Dataset for Emo | match |
| DATASET 18: EAV (EEG-Audio-Video) \| REF: EAV: EEG-Audio-Video Dataset for Emotion Recognition | 10.1038/s41597-024-03838-4 | Cr/Op | 2024 | EAV: EEG-Audio-Video Dataset for Emotion Recognition in Conver | match |
| DATASET 19: Voice-UI EEG Emotion Dataset \| REF: EEG Datase | 10.1038/s41597-024-03887-9 | Cr/Op | 2024 | EEG Dataset for the Recognition of Different Emotions Induced | match |
| DATASET 20: OpenNeuro Film Clips EEG (ds002721 / Daly Affe | 10.1038/s41597-020-0507-6 | Cr/Op | 2020 | Neural and physiological data from participants listening to a | match |
| DATASET 13: GAMEEMO | 10.17632/b3pn4kwpmn.3 | Da/Op | 2020 | Database for Emotion Recognition System Based on EEG Signals a | n/a |
| DATASET 18: EAV (EEG-Audio-Video) | 10.5281/zenodo.10205702 | Da/Op | 2023 | EAV: EEG-Audio-Video Dataset for Emotion Recognition in Conver | n/a |
| SUPP-ONLY DOI | 10.18112/openneuro.ds002722.v1.0.0 | Da/Op | 2020 | phBCMIcalibration | repository label |
| SUPP-ONLY DOI | 10.18112/openneuro.ds005540.v1.0.7 | Da/Op | 2025 | EmoEEG-MC: A Multi-Context Emotional EEG Dataset for Cross-Con | n/a |
| SUPP-ONLY DOI | 10.57760/sciencedb.14025 | Da/Op | 2025 | EmoEEG-MC: A Multi-Context Emotional EEG Dataset for Cross-Con | n/a |
| DENS data DOI | 10.18112/openneuro.ds003751.v1.0.2 | Da/Op | 2021 | Dataset on Emotion with Naturalistic Stimuli (DENS) | n/a |
| FACED data DOI (Synapse) | 10.7303/syn50614194 | Da/Op | 2023 | THU_EP | repository label |
| EVA-MED data DOI (ScienceDB) | 10.57760/sciencedb.23231 | Da/Op | 2025 | EVA-MED: An Enhanced Valence-Arousal Multimodal Emotion Datase | n/a |
| Voice-UI data DOI (figshare) | 10.6084/m9.figshare.c.7207839 | Da/Op | 2025 | EEG Dataset for the Recognition of Different Emotions Naturall | n/a |
| Daly ds002721 latest version | 10.18112/openneuro.ds002721.v1.0.3 | Da/Op | 2024 | An EEG dataset recorded during affective music listening | n/a |
| DATASET 21: SEED-MYA (descriptor, IEEE TAFFC) | 10.1109/TAFFC.2025.3586444 | Cr/Op | 2025 | SEED-MYA: A Novel Myanmar Multimodal Dataset for Enhancing Emo | match |
| DATASET 21: SEED-MYA (companion, IEEE EMBC) | 10.1109/EMBC53108.2024.10782660 | Cr/Op | 2024 | A Multimodal Myanmar Emotion Dataset for Emotion Recognition | match |
| DATASET 22: SEED-SD (descriptor, IEEE TAFFC) | 10.1109/TAFFC.2025.3572504 | Cr/Op | 2025 | Investigating the Effects of Sleep Conditions on Emotion Respo | match |

Two identifiers resolve to a repository-internal project label rather than the corpus title (ds002722 → “phBCMIcalibration”; syn50614194 → “THU_EP”); corpus identity was confirmed against the descriptor record in both cases, and neither is counted as a cross-registry conflict.

Identifiers checked on 12 September 2026, namely the descriptor record and the dataset-level deposit of each of the five corpora added at the second search pass (registries; registry year; title check): Emognition, 10.1038/s41597-022-01262-0 (Cr/Op; 2022; match) and Harvard Dataverse deposit 10.7910/DVN/R9WAF4 (Da/Op; 2020; match); K-EmoCon, 10.1038/s41597-020-00630-y (Cr/Op; 2020; match) and Zenodo deposit 10.5281/zenodo.3931963 (Da; 2020; match; verified in one registry, not indexed by Crossref or OpenAlex); ECSMP, 10.1016/j.dib.2021.107660 (Cr/Op; 2021; match) and Mendeley Data deposit 10.17632/vn5nknh3mn (Da/Op; 2021; match); LLaMAC, 10.1038/s41597-025-06165-4 (Cr/Op; 2025; match) and figshare deposit 10.6084/m9.figshare.28748696 (Da/Op; 2025; match); Mixed Emotion (Tsinghua), 10.1038/s41597-024-03676-4 (Cr/Op; 2024; match) and Zenodo deposit 10.5281/zenodo.11194571 (Da/Op; 2024; match).

### S20.1 Findings that materially qualify the openness results

(a) Dataset-level identifier absence. 13 of the 27 corpora (48.1%) carry no dataset-level persistent identifier and are reachable only through a descriptor-paper DOI or a laboratory landing page: DEAP, SEED, SEED-IV, SEED-V, SEED-VII, SEED-GER, SEED-FRA, SEED-MYA, SEED-SD, DREAMER, AMIGOS, MAHNOB-HCI, MPED. All five corpora added at the second search pass carry a dataset-level identifier, so all thirteen are among the 22 corpora identified at the first search pass. The absence is consequential rather than bibliographic: as S20.1(c) records, all three corpora whose access route had failed by the re-check are drawn from this list, and none of them had an alternative route to fail back to. The Zenodo deposit associated with DREAMER (record 546113) carries the journal article DOI 10.1109/JBHI.2017.2688239 rather than minting its own, so it is recorded here as a landing page rather than as a dataset identifier.

(b) Identifier collision. SEED-GER and SEED-FRA are counted as two resources but are backed by a single shared descriptor DOI (10.1088/1741-2552/ac5c8d, J. Neural Eng. 2022) and are distinguished only by separate BCMI landing pages. The collision is recorded rather than resolved by merging the two resources.

(c) Access-class corrections. Corrections were made at two dated checks and are reported separately, because they are of different kinds.
First check, 1 August 2026, class corrections against live repository evidence. DENS: OpenNeuro GraphQL returns public = true, License = CC0, latest snapshot v1.0.6 for accession ds003751, and DataCite resolves the versioned identifier 10.18112/openneuro.ds003751.v1.0.2, which is the deposit version cited here rather than the latest snapshot; DENS is therefore fully open, not gated. FACED: the Synapse entity bundle for syn50614194 returns restrictionLevel = OPEN with hasUnmetAccessRequirement = false and canPublicRead = true, but canDownload = false and isCertificationRequired = true for an anonymous caller, and the file inventory (Data.zip, EEG_Features.zip, Code.zip and others) is listable without an account while retrieval is not; FACED is therefore registration-gated rather than fully open.
Second check, 11 September 2026, three access-classification corrections. In each of the three the recorded route no longer functions, so the correction concerns route status rather than universality. DEAP and AMIGOS: the Queen Mary host answers on its root with HTTP 200 under a browser user agent, so the failure is not bot filtering, but /mmv/datasets/deap/ and /amigos/ both return HTTP 503 from the server's own Apache error page, and anaxagoras.eecs.qmul.ac.uk, the host that issues credentials, refuses the TCP connection outright. MAHNOB-HCI: mahnob-db.eu/hci-tagging/ returns HTTP 404, the application having been removed with only static files left behind. The Internet Archive dates the DEAP failure to 2025-12 at the latest and the MAHNOB-HCI failure to some time after 2024-05, and the control host bcmi.sjtu.edu.cn/home/seed/ answered HTTP 200 in the same check, so the result is not an artefact of the testing conditions. None of the three carries a dataset-level persistent identifier, so no alternative route exists to fall back on. All three are retained in the corpus and re-classified on a time dimension rather than excluded: the access_status field records route-live for the rest of the corpus and route-non-functional-at-recheck for DEAP, AMIGOS and MAHNOB-HCI (Supplementary Table S17), which preserves both the fact that the eligibility condition was met at corpus assembly and the fact that it was not met at the re-check. Excluding them would have removed the evidence for the finding.
A further correction changes an access class rather than a route status. EVA-MED had been recorded as fully open, but its deposit reverted to restricted at version 6 on 2025-12-18, and the class is corrected to conditional accordingly. With these corrections applied, and with the five corpora added at the second search pass classed against their recorded access routes, the access distribution of the 27 appraised corpora is fully open 8 (29.6%), registration- or licence-gated 15 (55.6%), conditional 4 (14.8%) and closed 0, as recorded in (f) below and in Supplementary Table S17.

(d) Licence corrections. Three, of which one was made at the first check and two at the re-check. EAV: the Zenodo record (10.5281/zenodo.10205702) returns access_right = ‘restricted’ with licence id ‘cc-by-4.0’, so the conditional access class is confirmed and the licence previously recorded (CC-BY-NC-ND) is corrected to CC-BY-4.0. FACED: the DataCite record returns an empty rightsList, so no licence is stated at the deposit, and the licence value previously recorded for it is withdrawn rather than replaced. EVA-MED: no licence is stated at the deposit either, on the same evidence. A corpus that states no licence is not thereby permissive, and both withdrawals lower Reusability; the direction is recorded here so that the reader can see that these two movements are attribute corrections and not consequences of the scoring rules.

(e) Held resources re-checked. EEGEMO (Zenodo 7398263) resolves and returns access_right = ‘restricted’ with no anonymous file enumeration, which confirms the decision to hold rather than score it. SEED-SD, previously held pending access verification, was released from hold and appraised: its official BCMI landing page and its IEEE Transactions on Affective Computing 2025 descriptor (10.1109/TAFFC.2025.3572504, verified) establish the same universally grantable application route accepted for the other SEED variants. SEED-MYA (10.1109/TAFFC.2025.3586444, verified) was added at the same check of the BCMI portal.

(f) Identifiers of the corpora added at the second search pass. The five corpora added at the second search pass were each resolved before appraisal, and each carries a dataset-level deposit rather than a descriptor record alone: Emognition 10.7910/DVN/R9WAF4 (Harvard Dataverse), K-EmoCon 10.5281/zenodo.3931963, ECSMP 10.17632/vn5nknh3mn (Mendeley Data), LLaMAC 10.6084/m9.figshare.28748696 and Mixed Emotion (Tsinghua) 10.5281/zenodo.11194571. Their ten identifiers, descriptor and deposit, were checked in this ledger on 12 September 2026 and are listed below the ledger table. Their access classes were adjudicated against the recorded access route and are entered in Supplementary Table S17 and in the corpus access distribution: Emognition registration- or licence-gated, K-EmoCon conditional, ECSMP fully open, LLaMAC fully open, Mixed Emotion (Tsinghua) conditional. No item of this ledger remains open.

(g) Identifier choice and the dataset life cycle. One dataset may carry several persistent identifiers minted at different points in its life cycle, and the richness of the registry record can differ between them. This review tested whether that difference is present in the appraised corpus and whether it affects any score. For the four OpenNeuro corpora in the corpus the DataCite record of the OpenNeuro accession was compared with the DataCite record of the corresponding NEMAR mirror on 12 September 2026. Each OpenNeuro record carries no subject terms, no description, no related identifiers and no rights statement. Each NEMAR record carries between 8 and 15 subject terms, a description of between 527 and 838 characters, between 4 and 7 related identifiers and a rights statement, and the EmoEEG-MC and Daly mirrors add funding references. The difference is therefore real and large. It does not move any score reported here, and the reason bounds the issue rather than dismissing it: the Findability criteria of this codebook ask whether a dataset-level identifier exists, resolves, is accompanied by a registered and indexed descriptor-level identifier, and is unique, and all four are satisfied by the OpenNeuro deposit alone, so all four corpora already score 4.0 on Findability and cannot rise. A criterion that scored registry metadata richness would be affected, and this review does not contain one. The scored-unit rule is stated in manuscript Section 2.1 and in full in Section S24.1 (Unit of analysis).

## S21 External Coverage Reconciliation (102-Unit Acquisition-Level Landscape)

The 27 appraised corpora were matched, name by name and identifier by identifier, against a separately built acquisition-level landscape of 102 affective-EEG resources assembled by the present authors under a different protocol, a different search architecture and a different cut-off (inclusive cut-off 31 July 2026); the landscape is therefore not an independent third-party source. The comparison was run over all 27 appraised corpora on 12 September 2026, so the containment result below covers every appraised corpus. The crosswalk of all 102 landscape units, recording for each unit the appraised corpus it matches, if any, the basis of the match (name, identifier or both, or membership of the Daly family), its status as matched or unmatched and any other contact it had with this review, was checked unit by unit on 25 September 2026 and is provided as data/landscape_crosswalk_102.csv in the reproducibility package (Reproducibility_package.zip). Because the landscape's unit is the acquisition, not the distributed corpus, screening its unmatched residual against this review's eligibility rule required re-extracting each unit at the corpus level; that screen is reported in Section S21.2, where each unmatched unit carries an eligibility disposition. This reconciliation tests coverage and exposes resources that this review's own search did not return. It tests the execution of the search as much as its scope: five of the 27 corpora are resources that this review's own recorded search strings return but that the first search pass did not enumerate (see the coverage statement below). The reconciliation does not enlarge the appraised corpus by itself; the corpus is 27 because of the two search passes recorded in Section S1, not because of anything the landscape contributed.

| **Reconciliation outcome** | **n** | **Note** |
| --- | --- | --- |
| Appraised corpora present in the landscape | 26 / 27 | Matched over all 27 appraised corpora on 12 September 2026. Twenty-six of the 27 appraised corpora are present. The single absentee is K-EmoCon, which is not silently missing: it appears in the landscape's own exclusion log under EX-FEATURE, on the ground that the release distributes eight relative band-power series at 32 Hz rather than sensor-by-time voltage EEG. |
| Appraised corpora absent from the landscape | 1 | K-EmoCon, excluded by the landscape protocol under EX-FEATURE. This review's eligibility criterion (a) asks whether scalp EEG is present, not whether voltage time series are distributed, which is why the two protocols differ on this one resource. The divergence is reported in manuscript Section 4.4 rather than resolved in this review's favour. |
| Landscape units matching no appraised corpus | 72 | Of the 102 acquisition-level units, 30 match an appraised corpus (the Daly family supplies five of those matches against one appraised corpus), leaving 72 unmatched. The 72 include EEGEMO, which this review holds rather than scores (Section S6), and six units from five records that the second search pass identified as eligible candidates and deferred because condition (3) was unverified (Section S4b). |
| Landscape units matching one appraised corpus each | 25 of the 30 matches | One-to-one at the canonical-name level for 25 matches; the remaining 5 matches are the Daly acquisition units, which map many-to-one onto a single appraised corpus. |
| Unit-of-analysis divergence | Daly family | This review treats the Daly resources as one canonical unit; the landscape splits them into five acquisition-level units (ds002721 to ds002725). |

### S21.1 Test of the field-application finding against the landscape

The landscape units not matched to an appraised corpus were screened for spatially situated elicitation, and because none of the five corpora added at the second search pass uses a spatial or immersive paradigm, the screen's yield is the same for the 72 units that remain unmatched. A resource was counted as spatially situated when the built or spatial environment itself, physical or virtual, was the eliciting factor; releases that use virtual reality only to present media or a performance (StageEmo-VR, DEJA-VU and MUSMET) and simulated driving (EmoRoad) fall outside this criterion and are not listed. The five resources below record scalp EEG with affect-relevant labels during spatially situated exposure; their identifiers, DataCite publication years and licences were verified against their registry and repository records on 25 September 2026. None is a benchmark corpus; all are single-study releases, none cited more than once in OpenAlex by 12 September 2026; none supplies benchmark splits, a standard schema or a training harness; and none appears in the reuse-dominant tier appraised in this review. The field-application finding of manuscript Section 4.3 is therefore stated as absence from the shared benchmark tier rather than absolute absence.

| **Resource** | **Year** | **Identifier (verified)** | **Licence** | **Spatial / built-environment paradigm; released labels; OpenAlex citations on 12 September 2026** |
| --- | --- | --- | --- | --- |
| Human Experience in Regulated Offices (HERO) | 2025 | 10.5281/zenodo.17957768 | CC-BY-4.0 (access request) | 24 adults, controlled indoor thermal regimes (~22 °C and ~30 °C) during office-like work; EEG + electrodermal activity + skin temperature; labels: phase-aligned valence, arousal, comfort and workload self-reports; citations: 0 |
| Stream-sound augmentation of traffic noise in VR | 2026 | 10.17632/v77ydkjwvn.1 | CC-BY-4.0 (derived EEG indices; raw EEG on request) | 50 participants (39 with usable EEG), virtual-reality reconstruction of a historic urban square under four traffic-sound levels with and without stream-sound augmentation; DataCite subject terms Psychology and Architecture; labels: ISO soundscape pleasantness and eight bipolar ratings; citations: 0 |
| EEG Dataset of Exploring Navigation in Virtual Reality | 2026 | 10.57760/sciencedb.23155 | CC0-1.0 | Virtual-reality spatial navigation and social exploration; labels: trial-level anxiety and willingness ratings (1 to 7) aligned to reward or punishment and to approach, interaction and avoidance conditions; citations: 1 |
| Audiovisual virtual reality for emotion induction | 2023 | 10.17632/y76vbw92y9.3 | CC-BY-4.0 | Virtual environments with a digital human speaker, eliciting anger and happiness with physiological recording; labels: Self-Assessment Manikin valence, arousal and dominance ratings; citations: 1 |
| The Emotional Rescue Dataset (SPACE) | 2026 | 10.17632/bxkgxg3ng4.1 | CC-BY-4.0 | 63 participants, immersive virtual spaces varied in colour pairing, form and area proportion (24 conditions per participant), with Self-Assessment Manikin pleasure, arousal, dominance and liking ratings; DataCite subject term Environmental Psychology; citations: 0 |

### S21.2 Screening of the unmatched landscape units against this review's eligibility criteria

Each of the 72 unmatched units was screened against this review's own eligibility criteria, and the resulting disposition record is reported here in full. Each unit was tested against criteria (a) to (e) of Section S13 (manuscript Section 2.3) using the charted fields of the landscape record rather than a fresh judgement, so the screen is reproducible: criteria (a), (b) and (e) are satisfied by construction, because the landscape protocol's own gates 1, 3 and 5 are equivalent to them; criterion (c) was tested on the presence of a resolvable persistent identifier; and criterion (d) was tested on the recorded access class and access evidence. The outcome is that 69 of the 72 unmatched units satisfy criteria (a) to (e) and 3 do not, all 3 failing criterion (d) because their access class is recorded as restricted or unavailable. Because criterion (d) was tested on that recorded access class, the result does not reflect the landscape's own access audit of 1 August 2026, which records 13 of the 69 eligible units as excluded for access (EX-ACCESS: no functioning public or application route to the sensor-time EEG was verified); the 69 / 3 result is reported on the recorded access class and is not adjusted for that audit, which conversely records two of the three units that fail criterion (d) here, both Science Data Bank deposits, as obtainable. This review therefore states plainly that a substantial body of eligible affective-EEG resources was not appraised here, and the count is measured rather than estimated. On the recorded access class, what separates those 69 resources from the appraised corpus is not eligibility but reuse. Adoption was measured for them on the same basis as Supplementary Section S22, namely the OpenAlex citation count of the resource's registered record, queried on 12 September 2026 (on 25 September 2026 for AFFEC and EEGEMO, which that query did not cover): 66 of the 69 are indexed, their median citation count is 0 with 52 of them at zero, three exceed ten citations and none exceeds one hundred, against a median of 94 and a maximum of 5044 for the 27 appraised corpora. The purposive bound this review states, namely the benchmark-relevant corpora that dominate reuse, is therefore supported by a measurement rather than by assertion, and the reader can see exactly which resources fall outside it. The difference describes the composition of the appraised sample; it does not remove the coverage limitation stated below. The per-unit disposition record, with name, first-release year as recorded in the landscape, access class, licence, persistent identifier, label summary, eligibility verdict, the criterion failed by each of the three ineligible units, and the OpenAlex citation count with its query date, is provided as data/landscape_unreached_screen.csv in the reproducibility package (Reproducibility_package.zip); the crosswalk that defines the 72 unmatched units is provided as data/landscape_crosswalk_102.csv (Section S21).

Coverage statement. This review does not claim exhaustive retrieval, nor does it claim that the unmatched landscape units lie beyond its search architecture: five eligible resources, Emognition, K-EmoCon, ECSMP, LLaMAC and Mixed Emotion (Tsinghua), are returned by the Scopus and Web of Science strings recorded in Section S1 under the document-type limits reported there, the recorded PubMed string returns all five without any limit, and the first search pass did not enumerate them; all five were identified and appraised at the second search pass. For those five the cause of non-retrieval at the first search pass was the execution of the search and the keeping of its record, not the boundary of the design, and two of them, K-EmoCon and Emognition, appear in the working notes of the first-pass census of 17 June 2026, Emognition with an explicit include decision, so the loss there occurred after the eligibility judgement rather than before it. This is a limitation of the first-pass retrieval and is reported as one. What this review does claim is a purposively bounded appraisal of the benchmark-relevant, persistently documented corpora that dominate reuse in EEG emotion recognition, together with the acknowledgement that the bound was not applied cleanly at the first search pass. The purposive-bounding defence covers resources outside the stated criteria; it does not cover resources inside them, and none of the five was outside them. The coverage check is reported so that the boundary and the failure to hold it are both visible rather than implied, and the corresponding limitation is stated in manuscript Section 4.4. Counts, proportions and conclusions in this review therefore describe the 27 appraised corpora, not the entire affective-EEG landscape.

# Supplementary Material, Sections S22–S25

## S22 Adoption Metric for All 27 Appraised Corpora

Supplementary Table S22. Adoption metric for all 27 appraised corpora. Adoption is operationalised as the citation count of each corpus descriptor record in OpenAlex, queried as a single batch on 12 September 2026 and reported alongside a count normalised by years since the descriptor publication year. Where the recorded identifier is a deposit accession rather than a descriptor paper, as for GAMEEMO and EAV, the descriptor paper is queried instead, and the substitution is noted in the transparency workbook (sheet S22_adoption); IESD has no descriptor paper and is indexed through its OpenNeuro accession, so all 27 corpora carry an adoption value. Across the 27, Spearman rho between adoption and the FAIR composite is -0.504 on raw counts and -0.571 on the age-normalised counts, and between adoption and AI-readiness is +0.176 and +0.203; the two openness correlations reach conventional significance (p = 0.007 and p = 0.002) and the two readiness correlations do not (p = 0.38 and p = 0.31). Splitting at the median citation count (94), the more-cited half (n = 14) has a mean FAIR composite of 2.586 against 3.150 for the less-cited half (n = 13). SEED-GER and SEED-FRA share one descriptor paper and therefore carry the same count. The per-corpus values are also provided as data/adoption_v2.json in the reproducibility package (Reproducibility_package.zip). Back-reference: manuscript Section 3.2.

| **ID** | **Dataset** | **Descriptor year** | **Citations (OpenAlex)** | **Citations per year** | **FAIR composite** | **AI-readiness** |
| --- | --- | --- | --- | --- | --- | --- |
| 1 | DEAP | 2011 | 5044 | 336.3 | 2.250 | 3.00 |
| 2 | SEED | 2015 | 2587 | 235.2 | 2.550 | 2.43 |
| 3 | SEED-IV | 2018 | 1262 | 157.8 | 2.750 | 2.50 |
| 4 | SEED-V | 2022 | 444 | 111.0 | 2.550 | 2.60 |
| 5 | SEED-VII | 2024 | 54 | 27.0 | 2.475 | 2.97 |
| 6 | SEED-GER | 2022 | 95 | 23.8 | 2.250 | 2.13 |
| 7 | SEED-FRA | 2022 | 95 | 23.8 | 2.250 | 2.20 |
| 8 | DREAMER | 2017 | 1111 | 123.4 | 2.500 | 2.83 |
| 9 | AMIGOS | 2018 | 702 | 87.8 | 2.250 | 3.23 |
| 10 | MAHNOB-HCI | 2011 | 1669 | 111.3 | 1.950 | 3.00 |
| 11 | FACED | 2023 | 96 | 32.0 | 2.750 | 3.07 |
| 12 | MPED | 2019 | 339 | 48.4 | 2.850 | 2.53 |
| 13 | GAMEEMO | 2020 | 183 | 30.5 | 3.175 | 2.50 |
| 14 | DENS | 2021 | 15 | 3.0 | 3.625 | 3.13 |
| 15 | Imagined Emotion Study (IESD, ds003004) | 2022 | 3 | 0.8 | 3.750 | 2.33 |
| 16 | EmoEEG-MC (ds005540) | 2025 | 6 | 6.0 | 3.950 | 3.33 |
| 17 | EVA-MED (Enhanced Valence-Arousal Multimodal Emotion Dataset) | 2025 | 5 | 5.0 | 2.650 | 2.77 |
| 18 | EAV (EEG-Audio-Video) | 2024 | 28 | 14.0 | 2.850 | 2.33 |
| 19 | Voice-UI EEG Emotion Dataset | 2024 | 8 | 4.0 | 3.175 | 2.60 |
| 20 | OpenNeuro Film Clips EEG (ds002721 / Daly Affective Music family) | 2020 | 20 | 3.3 | 3.900 | 2.80 |
| 21 | SEED-MYA | 2025 | 3 | 3.0 | 2.400 | 2.10 |
| 22 | SEED-SD | 2025 | 5 | 5.0 | 2.475 | 2.57 |
| 23 | Emognition | 2022 | 94 | 23.5 | 3.100 | 2.43 |
| 24 | K-EmoCon | 2020 | 169 | 28.2 | 3.025 | 3.13 |
| 25 | ECSMP | 2021 | 23 | 4.6 | 3.400 | 2.33 |
| 26 | LLaMAC | 2025 | 0 | 0.0 | 3.300 | 2.73 |
| 27 | Mixed Emotion (Tsinghua) | 2024 | 31 | 15.5 | 3.000 | 2.90 |

## S23 Construct-Overlap Matrix for the Appraisal Instruments

Across the 27 appraised corpora, scored under the codebook of Section S24, the openness-to-readiness association is close to zero (Spearman rho = 0.08) and turns negative once ontology level is held constant (partial rho = -0.32), while ontology level itself is the stronger correlate of readiness (rho = 0.68). The reduced composite (AS + TAS) / 2, which leaves out the Ontology-Maturity Sub-score (OMS), gives rho = -0.21 against the FAIR composite and rho = 0.40 against ontology level. These values are consistent with the review's argument that open access alone is inadequate as an index of reuse readiness.

Supplementary Table S23. Construct-overlap matrix for the appraisal instruments. Each row is a raw input recorded in the canonical attribute table; each column is an index reported in this review. A cell marked Yes indicates that the input feeds at least one of the atomic criteria of the scoring codebook (Section S24) that compose that index, so that correlations between indices sharing an input are partly definitional rather than wholly empirical. Two aspects of this overlap are stated here.
First, several raw inputs feed more than one index. BIDS-EEG compliance feeds FAIR Interoperability, OMS and the OFI Integration sub-index at once, and distribution format feeds FAIR Interoperability and the OFI Integration sub-index. Training-framework support feeds both FAIR Interoperability and TAS, so FAIR and the reduced composite (AS + TAS) / 2 still share that input. The persistent identifier feeds both FAIR Findability and the OFI Integration sub-index. The structure of the emotion representation feeds AS, OMS, the OFI and the ontology level. OMS is one third of the AI-readiness composite and is also the basis of the level assignment, which is why the ontology-readiness association of manuscript Section 3.5 is reported against both the full composite and the reduced composite (AS + TAS) / 2. The reduced composite does not remove every input shared with the ontology axis: label type is the largest single input to AS, at 41.7% of it, and label type also feeds TAS, the OFI and the ontology level. The reduced composite therefore attenuates the shared measurement rather than eliminating it, and the association it yields, rho = -0.21 against the FAIR composite, should be read with that residual in mind.
Second, the codebook makes one further overlap visible. Five criteria drawn from four codebook indices (OMS, SRS, RFS and Integration) read one and the same underlying fact: OMS-1 (formal ontology or knowledge graph), OMS-5 (external machine-readable vocabulary or ontology mapping), SRS-4 (controlled vocabulary mapped), RFS-1 (formal ontology or graph) and INT-1 (ontology mapped). No corpus in the appraised set attains any of them, because no corpus maps its affect labels to a controlled vocabulary or ontology. The L4 = L5 = 0 result, the unreached top of the OMS axis and the unreached top of the OFI Integration sub-index are accordingly three readings of one absence rather than three independent findings, and this review reports them as such. The observed sub-score correlations are consistent with the overlap disclosed here: AS and OMS correlate at 0.61, against 0.35 for AS and TAS and 0.28 for TAS and OMS.
Under the definition in manuscript Section 2.4.3, BIDS-EEG compliance does not contribute to the ontology level, which requires an explicit ontology or formal semantic mapping. Back-reference: manuscript Sections 2.4.1 to 2.4.3 and 3.5.

| **Raw input** | **FAIR-F** | **FAIR-A** | **FAIR-I** | **FAIR-R** | **AS** | **TAS** | **OMS** | **OFI** | **Ontology level** |
| --- | --- | --- | --- | --- | --- | --- | --- | --- | --- |
| Persistent identifier (DOI, OpenNeuro/Zenodo/Mendeley accession) | Yes |  |  |  |  |  |  | Yes |  |
| Access route clarity and universal grantability |  | Yes |  |  |  |  |  |  |  |
| Licence class (CC0/CC-BY > EULA/DUA > NC-ND) |  | Yes |  | Yes |  |  |  |  |  |
| Distribution format (BIDS-EEG / EDF / .set > .mat/.csv > .bdf/.dat) |  |  | Yes |  |  |  |  | Yes |  |
| BIDS-EEG compliance specifically |  |  | Yes |  |  |  | Yes | Yes | No (excluded by definition) |
| Datasheet present |  |  |  | Yes |  |  |  |  |  |
| 10-20 or dense montage metadata |  |  |  |  |  |  | Yes |  |  |
| Label type (discrete / dimensional / continuous) |  |  |  |  | Yes | Yes |  | Yes | Yes |
| Rater design (self / external / multi-rater) |  |  |  |  | Yes |  |  |  |  |
| Number of trials |  |  |  |  | Yes |  |  |  |  |
| Inter-annotator agreement reported |  |  |  |  | Yes |  |  |  |  |
| Supported-task breadth |  |  |  |  |  | Yes |  |  |  |
| Benchmark-split availability |  |  |  |  |  | Yes |  |  |  |
| Training-framework support (TorchEEG / EEGain / braindecode / MNE) |  |  | Yes |  |  | Yes |  |  |  |
| Structure of the emotion representation |  |  |  |  | Yes |  | Yes | Yes | Yes |
| Controlled vocabulary |  |  |  |  |  |  | Yes | Yes | Yes |
| Explicit ontology / formal semantic mapping |  |  |  |  |  |  | Yes | Yes | Yes |

## S24 Deterministic Scoring Codebook

This section specifies the conversion from source attributes to sub-scores. It sets out every atomic criterion, its category thresholds, the points assigned, the aggregation formula, the treatment of missing information, the rounding rules and the scale constraints.

The appraisal has two layers, set out below; this section specifies the second.

| **Layer** | **What it does** | **Status** |
| --- | --- | --- |
| Aggregation | sub-scores to composites and to every reported statistic | defined by the formulas in the main text; reproduced by scripts/score_engine.py, which regenerates the FAIR means, the AI-readiness mean and median, the level distribution, the cluster OFI means, the sub-score correlations, the weight-sensitivity table and the corpus-composition analysis |
| Conversion | source attributes to sub-scores | specified in this section and executed by scripts/codebook_score.py |

Index names and aggregation formulas are those defined in the main text, and the construct overlaps are those disclosed in Section S23. This codebook formalises those definitions as enumerated criteria; it does not alter what any index measures.

### S24.1 Binding policy layer

#### Missing information

Three states are distinguished, and the distinction is what prevents a resource from improving its score by reporting less.

| **Token** | **Meaning** | **Effect on the score** |
| --- | --- | --- |
| NA | the property cannot exist for this design | the criterion leaves the denominator |
| NR | the property could exist and the sources are silent | the criterion takes its floor and stays in the denominator |
| ABSENT | the sources positively state the property is missing | floor, stays in the denominator |

Worked demonstration. Take a resource scoring the floor on every Annotation Specificity criterion. Coding AS5 as NR gives a raw total of 0 over a denominator of 12.0, that is 1.0 on the scale. Coding AS5 as NA gives 0 over 10.0, which is also 1.0. Reporting less cannot raise the score. This property is asserted as a test in scripts/codebook_score.py --selftest rather than claimed in prose.

NA is admissible only where the design makes the property undefinable. The clearest case is inter-annotator agreement where the released labels are self-reported by the single recorded participant, so there are no annotators between whom agreement could be computed.

A fourth case is kept apart from NA, NR and ABSENT: a field not yet extracted at the frozen check date. That is a property of the coding pass, not of the resource, so the index that reads the field is withheld for that dataset and flagged; it is never imputed and never coded NA. Where a missing-information policy in Section S24.2 says that ABSENT withholds an index or makes it not scorable, it refers to this case. Where it scores the floor, it refers to the ABSENT state of the table above. No dataset is in this state, because every cell of the fields the codebook reads is filled (Section S24.4).

#### Rounding

| **Layer** | **Decimal places** | **Note** |
| --- | --- | --- |
| Sub-scores | 1 | Python round(): to the nearest value, a value lying exactly halfway in binary floating point going to the even digit (for example 2.25 to 2.2 and 3.25 to 3.2) |
| FAIR composite, OFI | 3 | mean of the sub-scores |
| AI-readiness | 2 | (AS + TAS + OMS) / 3 |
| Weights | exact fractions | one third, never the rounded 0.333, which sums to 0.999 and moves boundary cases |
| Rank statistics | unrounded inputs | rounding creates ties that understate discordance |

#### Bounds

Each sub-score is an affine map of a bounded point total, so it cannot leave its scale. Where every criterion takes its floor the result is the scale minimum exactly, and where every criterion takes its maximum the result is the scale maximum exactly. Both endpoints are verified in code for all ten point-based dimensions.

#### Unit of analysis

One row is one distributed corpus under one access route. SEED-GER and SEED-FRA share a descriptor identifier but are distributed as two corpora under two landing pages, so they are two rows, and the shared identifier is scored as a collision under FAIR-F. The Daly family spans several OpenNeuro accessions; the scored unit is the deposit named in the corpus record, with companion accessions recorded but not scored separately. More generally, a dataset may carry several persistent identifiers minted at different points in its life cycle, so the scored deposit is stated rather than inferred. It is the deposit that the corpus’s own data descriptor cites and that later reuse cites, which is normally the original deposit. A later mirror, or a redistribution preprocessed or reformatted for a particular model input, is a different artefact and is not scored. The identifiers that are not scored are listed in Section S20, and the registry differences between the OpenNeuro records and their NEMAR mirrors are reported in Section S20.1(g).

Deduplication was applied to resources before any scoring. Records were merged into one scored unit when they describe the same corpus. That covers several papers about one corpus, a mirror of a deposit, and companion deposits that the corpus descriptor presents as one data release, as for the Daly family, which is scored through ds002721. Corpora that share a descriptor paper but are released as separately named datasets with their own participants stay separate units, as SEED-GER and SEED-FRA do. Stream A was enumerated first, and Stream B contributed only resources that Stream A had not returned. Seed and anchor corpora reached by both streams are therefore attributed to Stream A (Sections S1 and S2).

#### Attribute provenance

Every cell of the attribute table carries a value, a source and a check date, with three exceptions. The fields year, condition3 and access_status have no __src or __date companion columns. In 27 cells the check date reads “undated (manuscript value)” (access_mode 13, descriptor_doi 9, dataset_pid 5): the value was taken from the supplementary section named in the __src cell and has no check date of its own. The fields raw_or_preprocessed and versioning are empty, with their companion cells, for the same 18 datasets (IDs 1 to 10, 12 and 21 to 27); the codebook does not read them. The table is supplied as data/attributes.csv in the reproducibility package (Reproducibility_package.zip), with the same content in data/attributes.json and with __src and __date companion columns, so any score can be traced to the page it was read from and the day it was read.

### S24.2 Criteria by index

Each index below is given with its scale, its number of criteria, its raw maximum and its aggregation formula, followed by one table row per criterion: the question the coder answers, the attribute field read, the ordered states with their points, the maximum points and the missing-information policy. The cells are abridged for layout: each state is shown by its label in data/codebook_v2.json, without its closing full stop and cut after its defining term or first clause where the label continues with a fuller definition or with observation notes; long questions are shortened; and each missing-information policy is condensed to its operative rule. In these cells and in Section S24.3, section numbers without an S prefix and Tables 2 and 3 refer to the manuscript. The complete text of every question, state definition, decision rule and missing-information policy is in data/codebook_v2.json in the reproducibility package.

#### FAIR-F

Feeds: FAIR composite. Scale 0 to 4. Criteria: 4. Raw maximum: 7.

Aggregation: score = 0 + (4 - 0) x (raw / denominator), where the denominator is the sum of the maxima of the criteria that remain after NA removal.

| **Criterion** | **Question** | **Attribute field** | **States and points** | **Max** | **Missing policy** |
| --- | --- | --- | --- | --- | --- |
| F1 | Does a persistent identifier exist that designates the DATA resource itself, and in what registration class? | dataset_pid | registered-dataset-PID = 4 / repository-accession-no-minted-DOI = 3 / portal-only-ID = 2 / no-locator = 0 | 4 | NR (registry searches run and returned nothing) is evidence of absence, not missingness: score the state the remaining evidence supports (portal-only if a locator exists, no-locator if none does). |
| F2 | When the identifier of record is resolved on the check date, does it land on the data resource, somewhere else, or nowhere? | pid_resolves | yes = 1 / redirects-elsewhere = 0.5 / no = 0 | 1 | NA when F1 = 0.0 (no-locator): there is nothing to resolve, so award 0.0 and record NA rather than pretending a check was run; F1 has already floored the score, so no second penalty is applied. |
| F3 | Is there a registered, index-resolvable identifier for the data descriptor, so the resource is discoverable through a scholarly index? | descriptor_doi | indexed-DOI = 1 / unindexed-or-informal = 0.5 / none = 0 | 1 | NR (no descriptor DOI recorded and none found by a Crossref query on the resource name) scores 0.0, since a descriptor that cannot be found by search is not indexed. |
| F4 | Does the identifier of record designate this dataset and no other resource in the corpus? | descriptor_doi | unique = 1 / collision = 0 | 1 | NR is not reachable: uniqueness is computed from identifiers already recorded, not observed in the field, so it is derivable whenever F1 and F3 are scorable. |

#### FAIR-A

Feeds: FAIR composite. Scale 0 to 4. Criteria: 5. Raw maximum: 10.

Aggregation: score = 0 + (4 - 0) x (raw / denominator), where the denominator is the sum of the maxima of the criteria that remain after NA removal.

| **Criterion** | **Question** | **Attribute field** | **States and points** | **Max** | **Missing policy** |
| --- | --- | --- | --- | --- | --- |
| A1 | Can an independent reader, starting from what the holder publishes, enumerate every operative step and every endpoint of the retrieval procedure without first contacting the holder to learn what the procedure is? | access_route | fully-specified-route = 3 / route-with-unstated-step = 2 / pointer-or-unpublished-procedure = 1 / no-route-recorded = 0 | 3 | NR (the field was examined and the holder publishes no procedure) scores 0, because silence is evidence about the resource. NA does not arise: every resource in scope has some retrieval concept, so a coder who writes NA here has mis-coded and must resolve to one of the four states. |
| A2 | Before applying, can a prospective requester read a statement of every condition they must satisfy, and know from that statement whether satisfying it yields access? | licence | conditions-published-and-sufficient = 3 / conditions-published-grant-discretionary = 2 / conditions-unpublished-or-generic = 1 / no-conditions-recorded-or-contradictory = 0 | 3 | NA is admissible here and is NOT a zero: it is the coding for a verified anonymous route with no eligibility condition, and it resolves to 3 through limb (b) of the top state. |
| A3 | Do the published terms and the operative steps of the route leave the resource obtainable by a requester in any country, on the same conditions? | access_route | universal = 2 / region-contingent-in-practice = 1 / nationality-or-region-restricted = 0 | 2 | NR (the terms were examined and are silent on nationality and region) resolves to the top state, per the decision rule of that state in data/codebook_v2.json; the guard is a restriction test. |
| A4 | At the frozen check date, was the documented route confirmed against the live repository or registry, rather than inferred from the descriptor? | access_status | route-live = 2 / route-non-functional-at-recheck = 0 | 2 | NR or ABSENT both score 0 here and are reported as unverified, which is the one place in this index where an unfilled cell scores rather than blocking. |
| A5 | What does the access-mode class contribute to FAIR-A? Nothing, in every state. This criterion states an exact score contribution for every possible access_mode state, as the codebook does for every attribute, so that no coder silently re-imports access mode as a tie-breaker. | access_mode | FullyOpen = 0 / Academic = 0 / Conditional = 0 / closed = 0 / pending-or-missing = 0 | 0 | Every state, including NR, NA, ABSENT and pending, contributes exactly 0.0 points. This criterion can neither block nor alter the index; it is a declaration, and it is the only criterion in the set that reads access_mode at all. |

#### FAIR-I

Feeds: FAIR composite. Scale 0 to 4. Criteria: 5. Raw maximum: 16.

Aggregation: score = 0 + (4 - 0) x (raw / denominator), where the denominator is the sum of the maxima of the criteria that remain after NA removal.

| **Criterion** | **Question** | **Attribute field** | **States and points** | **Max** | **Missing policy** |
| --- | --- | --- | --- | --- | --- |
| I1 | Taking every format in which EEG signal data is actually distributed, which is the highest-ranked band on the manuscript's published ordering, where the ordering is standard EEG interchange container > generic numeric or text container > device-native or runtime-serialised binary? | distribution_format | STANDARD_TIMESERIES = 6 / GENERIC_TIMESERIES = 3 / DEVICE_NATIVE_ONLY = 1 / NOT_DISTRIBUTED_OR_NR = 0 | 6 | NR (distribution_format unrecorded or no retrievable source): score 0, raise flag NR_I1, keep the 6 points in the denominator. NA: never applicable, every appraised resource distributes something or is recorded as distributing nothing. |
| I2 | Is the deposit organised as a BIDS-EEG dataset, and what compliance evidence was retrievable at the check date? | bids_compliant | VALIDATED = 4 / DECLARED = 3 / PARTIAL = 2 / NO = 0 / NR = 0 | 4 | NR: score 0, raise flag NR_I2, denominator unchanged. NA: never applicable, since every deposit either is or is not organised as BIDS. ABSENT (state NO): score 0 with no flag; NO and NR are both 0 points but must be recorded as different states, because NO is a finding and NR is a gap in the harvest. |
| I3 | Does the band selected at I1 by itself deliver the complete EEG release (all subjects, all sessions, all trials, all EEG channels), or is a lower-ranked container needed for part of it? | distribution_format | FULL = 2 / PARTIAL = 1 / NOT_DETERMINABLE = 0 | 2 | NR: score 0, raise flag NR_I3, denominator unchanged. NA: never applicable; where I1 found nothing distributed the criterion resolves to the ABSENT branch of NOT_DETERMINABLE at 0 points rather than being dropped, so that a resource distributing nothing cannot raise its normalised score by shrinking its own denominator. |
| I4 | Are the affect ratings, class labels and event or trigger markers distributed in an open, directly parseable file, or only inside a container that must be opened with runtime or vendor software? | distribution_format | OPEN_TABLE_OR_SIDECAR = 2 / IN_CONTAINER = 1 / UNRESOLVABLE_OR_NR = 0 / NA = 0 | 2 | NR: score 0, raise flag NR_I4, denominator unchanged at 16. NA (no labels distributed at all): criterion dropped, denominator 14; this is the only NA-eligible criterion in the index. ABSENT: not distinguishable from NA here, so it is coded NA. |
| I5 | Does code exist, retrievable at the check date, that loads or extracts features from THIS corpus specifically, and is it a mainstream-framework integration or a reader shipped by the depositors? | loader_support | MAINSTREAM_FRAMEWORK = 2 / DATASET_READER = 1 / NONE = 0 | 2 | NR: score 0, raise flag NR_I5, denominator unchanged. NA: never applicable. ABSENT: score 0 with no flag. The two-point, three-state form credits integration in a mainstream framework above a reader shipped by the depositors. |

#### FAIR-R

Feeds: FAIR composite. Scale 0 to 4. Criteria: 4. Raw maximum: 8.

Aggregation: score = 0 + (4 - 0) x (raw / denominator), where the denominator is the sum of the maxima of the criteria that remain after NA removal.

| **Criterion** | **Question** | **Attribute field** | **States and points** | **Max** | **Missing policy** |
| --- | --- | --- | --- | --- | --- |
| R1 | Which licence class governs the DATA FILES (not the descriptor article) at the frozen check date? | licence_class | CC0 / PDDL / equivalent public-domain dedication applied to the data files = 4 / CC-BY (any version) applied to the data files = 4 / CC-BY-SA, ODbL, or another Open-Definition licence whose only added condition is reciprocal (share-alike) = 3.5 / Research EULA or DUA: a named, signable or click-through agreement restricting use to academic/non-commercial research, with clause text published = 2 / CC-BY-NC, CC-BY-ND, CC-BY-NC-ND, or any Creative Commons licence carrying an NC and/or ND element on the data files = 1 / No data licence stated at all = 0 | 4 | NR (coder searched the repository record, any LICENSE/terms file, DataCite and OpenAIRE rights fields, and found nothing): this is NOT a missing code for R1; it IS the substantive state "no data licence stated", 0 points, and must be recorded as such with the negative-evidence string. |
| R2 | Before applying for access, can a reuser read clause text stating both what may be redistributed and whether commercial use is permitted? | licence | Both redistribution status and commercial-use status stated in readable clause text = 1 / One of the two stated, or terms appear only as an access label plus a citation request with no clause text = 0.5 / No reuse or redistribution terms found in any location = 0 | 1 | NR: scores 0 - the construct is about what a prospective reuser can actually read, so unfindable terms are functionally absent, and the negative-evidence string (search targets and check date) is recorded. |
| R3 | Is there documentation that tells a reuser what every distributed file and every variable or column contains? | datasheet | File- and variable-level dictionary distributed with the data, or published verbatim on the resource's own landing page = 2 / Structural documentation only, or the variable-level dictionary exists only inside the descriptor article = 1 / No file-level or variable-level documentation in any location = 0 | 2 | NR (searched landing page, shipped file manifest, descriptor and linked repositories; none found): scores 0, recorded with the negative-evidence string. |
| R4 | Does the documentation cover collection motivation and protocol, subject demographics, ethics or consent scope, known defects or missing data, and maintenance or version history? | datasheet | A formal Datasheets-for-Datasets or model-card document, OR all five elements present as a documented set = 1 / Some but not all five elements present, in any retrievable location = 0.5 / None of the five elements present anywhere = 0 | 1 | NR: scores 0. NA: never admissible; recode as NR, since a descriptor-less resource simply has none of the five. ABSENT (datasheet cell never extracted): R4 not scorable, FAIR-R not computed. |

#### AS

Feeds: AI-readiness (AIR). Scale 1 to 4. Criteria: 5. Raw maximum: 12.

Aggregation: score = 1 + (4 - 1) x (raw / denominator), where the denominator is the sum of the maxima of the criteria that remain after NA removal.

| **Criterion** | **Question** | **Attribute field** | **States and points** | **Max** | **Missing policy** |
| --- | --- | --- | --- | --- | --- |
| AS1 | What affect-label scheme or schemes are actually released for the scored units, and how fine is the class or axis structure? | label_model | mixed (discrete >=4 classes AND dimensional >=2 axes) = 3 / dimensional with >=3 axes, or discrete with >=8 classes = 2.5 / dimensional with exactly 2 axes, or discrete with 4-7 classes = 2 / discrete 3-class = 1 / discrete 2-class (binary or bipolar) = 0.5 / no released per-unit affect label = 0 | 3 | NR (label_model recorded as 'not reported'): 0 points, criterion retained in the denominator, because an undocumented label scheme is a real deficit of annotation specificity. |
| AS2 | At what temporal resolution are the released affect labels attached to the signal? | label_granularity | continuous or interval-resolved timeseries = 2 / one label set per trial, trial boundaries defined = 1 / one label per session or per block only = 0.5 / granularity not documented = 0 | 2 | NR: 0 points, criterion retained, because granularity that cannot be established is granularity that cannot be exploited. NA: not admissible; every released label attaches to some unit. |
| AS3 | Who produced the label that is actually distributed, and how many independent raters contributed per scored unit? | rater_design | multi-rater (>=3 independent annotators of the released units) = 3 / two independent perspectives on the released units = 2.5 / self-report by the recorded participant, released as the label = 2 / stimulus-defined label with a documented per-trial self-report = 1.5 / stimulus-defined only = 1 / algorithmic or automatic labels only = 0.5 / rater design not documented = 0 | 3 | NR: 0 points, criterion retained, because an undocumented rater chain is exactly the reliability deficit AS is meant to register. NA: not admissible; every released label has some producer. |
| AS4 | How many distinct label-bearing units are released per subject? | trials | U >= 60 = 2 / 30 <= U <= 59 = 1.5 / 15 <= U <= 29 = 1 / U < 15 = 0.5 / U not derivable = 0 | 2 | NR (trials recorded as 'not reported'): 0 points, criterion retained, because a corpus whose annotation volume is undocumented cannot evidence annotation specificity. |
| AS5 | Is a between-annotator agreement coefficient reported for the labels that are distributed, and how strong is it? | inter_annotator_agreement | coefficient over released labels, strong = 2 / coefficient over released labels, weak or unquantified = 1.5 / NR: released units multi-annotated, no coefficient published = 0 / NA: one rater per released unit = 0 | 2 | The design test governs. (i) If every released label carries one rater per item, AS5 is not definable, takes the NA sentinel, and is dropped from both numerator and denominator, so raw_max for AS falls from 12.0 to 10.0 for that dataset (23 of 27). (ii) If two or more independent annotators produced the released labels but no coefficient is published, the criterion is floored at 0 and stays in the denominator (NR; FACED and EAV). (iii) A reliability statistic that is not between annotators of the released units, such as a concordance between the stimulus-intended label and the participant's self-report, is recorded under AS5__out_of_scope_statistic and reported descriptively; it does not score. Only an agreement coefficient between annotators of the released units scores, so the decision to report a statistic can neither override the design test nor lower the score. |

#### TAS

Feeds: AI-readiness (AIR). Scale 1 to 4. Criteria: 6. Raw maximum: 12.

Aggregation: score = 1 + (4 - 1) x (raw / denominator), where the denominator is the sum of the maxima of the criteria that remain after NA removal.

| **Criterion** | **Question** | **Attribute field** | **States and points** | **Max** | **Missing policy** |
| --- | --- | --- | --- | --- | --- |
| TAS-1 | Does the distributed label scheme support classification only, or classification and dimensional regression both? | label_model | Numeric affect scale distributed (dimensional or mixed) = 2 / Discrete categories only: two or more named emotion categories, self-reported or stimulus-defined, and no numeric affect rating is distributed = 1 / No emotion label scheme is distributed with the signals, or the scheme cannot be determined from the released files = 0 | 2 | NR (field checked, descriptor and repository silent on the label scheme): score 0, the zero band; absence of a documented target is itself the finding. |
| TAS-2 | Are the affect labels resolved in time within a trial, so that a continuous-tracking (time-resolved regression) target exists? | label_granularity | Time-resolved within trial: a continuous annotation trace, a fixed-interval annotation (for example one label per 5 s), or per-segment or per-event labels with stated onsets = 1 / One label per trial, per clip, per block or per session; the signal is time-resolved but the label is not = 0 | 1 | NR (granularity not stated anywhere): score 0. Silence is not evidence of a continuous target, and the construct credits demonstrated task support only. |
| TAS-3 | Is at least one non-EEG, time-locked stream actually distributed with the EEG? | modalities | At least one non-EEG stream is distributed and time-locked to the EEG = 1 / EEG only, or additional streams were recorded but are not in the distribution = 0 | 1 | NR (modality inventory not stated): score 0. NA: not admitted, because every record has a modality inventory, at minimum EEG alone; a coder entry of NA is recoded NR. |
| TAS-4 | Which of the two transfer tasks named by the manuscript can a reuser actually construct from the release? | supported_tasks | Both transfer tasks admitted. = 2 / Exactly one transfer task admitted. = 1 / Neither transfer task admitted. = 0 | 2 | NR (the supported-task determination was attempted and the evidence does not settle it): score 0 for the unsettled test only; the settled test still scores. |
| TAS-5 | Is an evaluation partition documented, is it subject- or session-wise, and does anything runnable accompany it? | benchmark_splits | Subject-wise or session-wise partition documented AND accompanied by something runnable: baseline code, partition files, or a public leaderboard = 3 / Either (a) a subject-wise or session-wise partition is documented but nothing runnable accompanies it, or (b) runnable baseline code or partition files are distributed that implement a documented within-subject or within-session partition = 2 / An evaluation protocol is reported in the descriptor only: a stated train/test or cross-validation scheme with baseline results, no partition files and no distributed code, OR code is distributed whose fold scheme is nowhere specified = 1 / No partition, no evaluation protocol and no baseline code. Code released for visualisation or technical validation only does not qualify = 0 | 3 | NR (the descriptor and repository were checked and are silent): score 0, identical to a recorded value of "none"; for this criterion silence and absence are the same observable state, and the coder must record which of the two it is in the field text. |
| TAS-6 | How far does an existing EEG machine-learning toolchain carry the corpus, from a corpus-specific dataset class down to bespoke parsing? | loader_support | loader_support records TorchEEG or EEGain: a deep-learning EEG training framework ships a dataset class specific to this corpus = 3 / loader_support records braindecode: a training framework ingests the distributed format through its generic pipeline, but no corpus-specific dataset class exists = 2 / loader_support records MNE: a standard analysis reader opens the distributed files without bespoke parsing, but no training framework is involved = 1 / loader_support records none: no named framework or toolbox reads the corpus; ingestion requires a user-written or depositor-supplied bespoke parser = 0 | 3 | NR (loader availability checked and none found): score 0; this is the same observable state as a recorded value of "none". NA: not admitted, because the question applies to every distributed corpus; a coder entry of NA is recoded NR. |

#### OMS

Feeds: AI-readiness (AIR). Scale 1 to 4. Criteria: 5. Raw maximum: 16.

Aggregation: score = 1 + (4 - 1) x (raw / denominator), where the denominator is the sum of the maxima of the criteria that remain after NA removal.

| **Criterion** | **Question** | **Attribute field** | **States and points** | **Max** | **Missing policy** |
| --- | --- | --- | --- | --- | --- |
| OMS-1 | What is the most formal emotion-representation scheme that is actually distributed with the data, judged from the released label scheme (class names, dimensions, scale ranges, intensity, exclusivity) recorded in label_model? | label_model | L4/L5 formal ontology or knowledge graph = 6 / Co-registered dual representation = 5 / Single dimensional / relational model = 4 / Discrete taxonomy = 2 / Flat label = 0 | 6 | NR (label_model states that the label scheme is not reported, or names only a stimulus category with no released label file): score 0 points, the flat-label state, because an undocumented scheme carries no demonstrable representational structure. |
| OMS-2 | Are the electrode identities referred to a named standard layout, and is a position or channel-order file actually distributed with the data? | electrode_metadata | Standard named and positions/order distributed = 2 / Standard nomenclature only, or standard not named = 1 / Naming absent, non-standard, or internally contradictory = 0 | 2 | NR (the documentation states that electrode metadata is not reported): score 0 points, the naming-absent state. NA: not admissible; an EEG dataset always has a montage. |
| OMS-3 | How many scalp EEG channels are actually distributed, counting signal electrodes only? | channels_montage | Dense array = 2 / Standard-density clinical/research cap = 1 / Low density, or count not stated = 0 | 2 | NR (the count is stated as not reported, or is given only as a device name with no number): score 0 points, which is the state that already names this case, because an unstated density cannot evidence a dense montage. |
| OMS-4 | Is the distribution organised as BIDS-EEG, and at what level of verification? | bids_compliant | yes-validated = 3 / yes-claimed = 2 / partial = 1 / no = 0 | 3 | NR (BIDS status not stated anywhere in the descriptor or on the repository page, and no BIDS structures are visible in the file listing): score 0 points, the "no" state, because BIDS compliance is a positive, inspectable property and its absence from a fully listed distribution is itself the finding. |
| OMS-5 | How formally are the affect-label terms defined: by an external machine-readable vocabulary, by a distributed term dictionary or a graded intensity scale, by prose only, or not at all? | controlled_vocabulary | External machine-readable vocabulary or ontology mapping = 3 / Distributed term dictionary, or continuous-intensity labels = 2 / Closed label list documented in prose only = 1 / Label term semantics not documented = 0 | 3 | NR (controlled_vocabulary = "none" or "not reported"): this is a scored state, not missing data. Score 1 point where label_model documents a closed label list with codes, 2 points where label_model shows graded-intensity labels, and 0 points where neither can be established. |

#### SRS

Feeds: OFI. Scale 1 to 4. Criteria: 4. Raw maximum: 6.

Aggregation: score = 1 + (4 - 1) x (raw / denominator), where the denominator is the sum of the maxima of the criteria that remain after NA removal.

| **Criterion** | **Question** | **Attribute field** | **States and points** | **Max** | **Missing policy** |
| --- | --- | --- | --- | --- | --- |
| SRS-1 | How many mutually distinguishable affect states does the RELEASED label scheme resolve? Count two quantities from label_model. K = the number of distinct named affect classes or emotion terms released as labels attached to signal units (per trial, per segment, per session or continuous). M = the number of released appraisal dimensions. Both counts include only label properties actually distributed with the data and attached to the recorded units. With g = [K>=4] + [K>=7] + [M>=2] + [M>=4], the state is coarse when g = 0, moderate when g = 1 and rich when g >= 2. | label_model | coarse (Table 3 L1 grain) = 0 / moderate = 1 / rich = 2 | 2 | NR (label_model present but states the scheme is not reported at the resolution needed to count K and M): score 0 and flag NR on the criterion. NA: inadmissible, because a dataset with no affect label model fails the inclusion criteria. |
| SRS-2 | What relation, if any, does the released label scheme state among its own labels? Read label_model and classify against the Table 3 anchors. Use the basic-emotion family test: the family is happiness/joy, sadness, fear, disgust, anger, surprise, and a class set is taxonomic when at least three family members appear alongside a neutral or baseline class. | label_model | relational or dimensional (Table 3 L3 or above) = 2 / taxonomic (Table 3 L2) = 1 / flat (Table 3 L1) = 0 | 2 | NR: score 0 (flat) and flag NR. NA: inadmissible, same reason as SRS-1. ABSENT: SRS withheld for that dataset, not imputed. No NR or ABSENT case occurs in the corpus: all 27 label_model values state enough to place the scheme on the Table 3 ladder. |
| SRS-3 | Does the released label scheme record a graded magnitude OVER AND ABOVE the structure already credited in SRS-2? The 'over and above' clause is what keeps this criterion from re-counting the dimensional coordinates that SRS-2 has already rewarded: for a purely dimensional corpus the coordinates are the SRS-2 credit, so they earn nothing here. | label_model | absent = 0 / present = 1 / not reported = 0 | 1 | NR is an explicit coding state, not a silent default: it scores 0 and carries a flag, and any SRS containing an NR flag is reported as a lower bound. |
| SRS-4 | Is any released affect label TERM mapped to an external controlled vocabulary or ontology term, in the distributed files or in the record metadata? | controlled_vocabulary | none = 0 / mapped = 1 / not reported = 0 | 1 | NR -> 0 with flag. NA (a corpus with no label vocabulary that could be mapped) -> 0 with flag, scored identically to 'none' but recorded distinctly. ABSENT -> 0 with flag; uniquely among the four criteria, ABSENT does not withhold the index here, because no corpus reaches L4, the level at which a formal semantic mapping first appears. Because SRS-4 is 0 for all 27 corpora, the highest SRS attainable in this corpus is 3.5. |

#### RFS

Feeds: OFI. Scale 1 to 4. Criteria: 4. Raw maximum: 9.

Aggregation: score = 1 + (4 - 1) x (raw / denominator), where the denominator is the sum of the maxima of the criteria that remain after NA removal.

| **Criterion** | **Question** | **Attribute field** | **States and points** | **Max** | **Missing policy** |
| --- | --- | --- | --- | --- | --- |
| RFS-1 | What is the most formal relational structure over the affect terms that the resource actually distributes with the data? Code the single highest state the distributed materials satisfy. | label_model | formal-ontology-or-graph = 4 / dimensional-coordinate = 3 / graded-discrete = 2 / unordered-discrete-4plus = 1 / flat = 0 / no-affect-labels = 0 | 4 | NR (label_model = not documented at the check date): 0 points, flagged NR in the ledger. The index measures derivability from what the resource provides, so an undocumented scheme provides nothing to derive from; this is a substantive 0, not an imputation. |
| RFS-2 | Is each distributed affect value a number on a scale whose range and endpoint anchors are stated, so that magnitudes and distances between affect terms are computable? | label_model | fully-specified-numeric = 2 / partly-specified-numeric = 1 / nominal-only = 0 | 2 | NR of the scale specification while numeric affect values are visibly present in the distributed files: 1 point, because this is exactly the partly-specified-numeric state, not a missing-data case. |
| RFS-3 | How many distinct affect-representation schemes are recorded on the same annotation units and distributed, so that cross-scheme correspondences can be tabulated? | label_model | two-or-more-schemes = 2 / one-scheme-plus-auxiliary = 1 / single-scheme = 0 | 2 | NR: 0 points, flagged NR. Only schemes verified as distributed and keyed to the annotation units are counted, so an undocumented second stream cannot earn points. |
| RFS-5 | Is the eliciting stimulus identified per annotated unit, so that the emotion-to-stimulus edge of a relation model can be instantiated? | stimulus_paradigm | per-unit-stimulus-identified = 1 / experiment-level-only = 0 | 1 | NR: 0 points, flagged NR. ABSENT in the structural sense (the design has no external eliciting stimulus at all: resting-state, spontaneous affect, or self-induced states with no per-unit cue) is coded NA rather than 0, and the criterion is then dropped from the denominator. No dataset in the corpus takes this path. |

#### Integration

Feeds: OFI. Scale 1 to 4. Criteria: 4. Raw maximum: 11.

Aggregation: score = 1 + (4 - 1) x (raw / denominator), where the denominator is the sum of the maxima of the criteria that remain after NA removal.

| **Criterion** | **Question** | **Attribute field** | **States and points** | **Max** | **Missing policy** |
| --- | --- | --- | --- | --- | --- |
| INT-1 | To what depth are the released terms (affect labels, events, channels) bound to an externally defined, published vocabulary rather than to free text or bare integer codes? | controlled_vocabulary | ONTOLOGY_MAPPED = 3 / STANDARD_VOCAB_APPLIED = 2 / RECORD_LEVEL_CONTROLLED_TERM = 1 / NONE = 0 | 3 | ABSENT (linkage verified not to exist, the usual extraction finding, recorded as "none" / "None found") = 0 points, a true zero, and the record is NOT flagged. |
| INT-2 | Is the dataset distributed as a conformant BIDS-EEG tree, and is that conformance evidenced rather than asserted? | bids_compliant | yes-validated = 3 / yes-claimed = 2 / partial = 1 / no = 0 | 3 | ABSENT (verified non-conformance) = 0 points, a true zero, not flagged. NR (BIDS status not checked) = 0 points AND flag INT-2-NR on the record; do not infer conformance from repository identity unless the repository validates on ingest, which is itself the yes-validated rule and therefore evidence, not imputation. |
| INT-3 | In the highest-ranked format in which the EEG signal itself is actually distributed, how far is that format an open EEG interchange standard rather than a generic or vendor-native container? | distribution_format | EEG_STANDARD = 3 / GENERIC_OPEN_CONTAINER = 2 / VENDOR_NATIVE_DOCUMENTED = 1 / OPAQUE_OR_UNSTATED = 0 | 3 | ABSENT cannot occur: a dataset that distributes no EEG signal is out of corpus by criterion EX-1 and is never scored. NR (format not recorded) = 0 points AND flag INT-3-NR; do not infer a format from the acquisition hardware. |
| INT-4 | Does an identifier for the data itself, distinct from the descriptor-paper DOI, link the dataset into an external identifier registry? | dataset_pid | REGISTERED_DATASET_DOI = 2 / PORTAL_ONLY_ACCESSION = 1 / NONE = 0 | 2 | ABSENT (a registry search was run and returned nothing, the usual extraction finding, recorded as "none - no dataset-level PID exists") = 0 points, a true zero, not flagged. |

#### OntologyLevel

Feeds: reported directly (RQ3). Scale 1 to 5. Criteria: 6. Raw maximum: 2.

The level is an ordered decision procedure, not a point total. The criteria below are evaluated in order and the level is fixed by the first gate that fires.

| **Criterion** | **Question** | **Attribute field** | **States and points** | **Max** | **Missing policy** |
| --- | --- | --- | --- | --- | --- |
| ONT-C1 | Does the corpus distribute an affect graph in which individual emotion, stimulus and signal INSTANCES are linked by typed relations, and over which entailment or graph query is actually supported (an ontology-backed triple store, an RDF/OWL instance graph, a SPARQL endpoint, or a released reasoner/query interface over the affect terms and their instances)? | controlled_vocabulary | INSTANCE-LINKED GRAPH PRESENT = 5 / ONTOLOGY OR RELATION MODEL PRESENT BUT CLASS-LEVEL ONLY = 0 / ABSENT: controlled_vocabulary = none = 0 / NR: controlled_vocabulary not yet coded for this dataset = 0 / NA: the design admits no affect graph = 0 | 5 | NR, NA and ABSENT are all treated identically here: the gate does not fire and the procedure falls through to ONT-C2. L5 never fires on missing information, because it asserts the presence of a reasoning-capable artefact. |
| ONT-C2 | Do the corpus's affect labels carry an explicit emotion ontology, a formal semantic mapping to one, or an equivalent machine-interpretable relation model over the affect labels themselves, that is, a released, machine-parseable artefact that asserts relations BETWEEN the affect terms? | controlled_vocabulary | EXPLICIT EMOTION ONTOLOGY = 4 / FORMAL SEMANTIC MAPPING = 4 / EQUIVALENT MACHINE-INTERPRETABLE RELATION MODEL = 4 / NEAR-MISS, REPOSITORY TAXONOMY = 0 / NEAR-MISS, CITED PSYCHOLOGICAL INSTRUMENT = 0 / NEAR-MISS, NON-AFFECT STANDARD NOMENCLATURE = 0 / NEAR-MISS, THIRD-PARTY ALGORITHMIC ANNOTATION VOCABULARY = 0 / NEAR-MISS, STRUCTURED METADATA RECORD OF UNVERIFIED CONTENT = 0 / ABSENT: controlled_vocabulary = none = 0 / NR: controlled_vocabulary not yet coded = 0 | 4 | Identical to ONT-C1: NR, NA and ABSENT all leave the gate closed, because L4 asserts the presence of a released artefact and absence of evidence is not evidence of presence. |
| ONT-C3 | Is the corpus BIDS-EEG compliant, and what does that contribute to OntologyLevel? | bids_compliant | yes-validated: passes the BIDS validator = 0 / yes-claimed: BIDS asserted but unvalidated = 0 / partial: some BIDS structure, incomplete = 0 / no: not BIDS = 0 / NR: BIDS status not coded = 0 | 0 | Every state, NR and NA included, contributes exactly 0.0 levels. This criterion can neither raise nor lower OntologyLevel and is never a reason to mark a dataset unscorable. BIDS validation therefore contributes nothing to OntologyLevel, although it is credited in FAIR-I (I2) and in OFI Integration (INT-2). |
| ONT-C4 | Does the corpus ship an event-marker or label-column dictionary, and what does that contribute to OntologyLevel? | event_schema | BIDS events.tsv with an emotion-label column and a JSON sidecar = 0 / Plain-text or tabular integer-code dictionary = 0 / Column semantics documented in descriptor prose only = 0 / not reported: no event or label dictionary of any kind = 0 / NR: event_schema not coded = 0 | 0 | Every state, NR and NA included, contributes exactly 0.0 levels; the criterion can neither raise nor lower the level and never makes a dataset unscorable. It is stated separately from ONT-C3 because a machine-readable data schema takes two observable forms in this corpus, a distribution standard and an event dictionary. |
| ONT-C5 | Does the label scheme actually distributed with the EEG records carry (a) at least one named affect dimension recorded as a coordinate independent of any categorical label, or (b) a released multi-term affect profile expressing relations among the labels, or (c) a discrete affect inventory of more than eight distinct categories? | label_model | (a) NAMED AFFECT DIMENSION RELEASED = 3 / (b) RELEASED MULTI-TERM AFFECT PROFILE = 3 / (c) DISCRETE INVENTORY LARGER THAN EIGHT = 3 / NEGATIVE: DISCRETE INVENTORY OF TWO TO EIGHT AFFECT QUALITIES, NO RELEASED AFFECT DIMENSION = 0 / NEGATIVE: VALENCE-POLARITY INVENTORY ONLY = 0 / NEGATIVE: PER-CATEGORY INTENSITY MAGNITUDE = 0 / NEGATIVE: AFFECT DIMENSION COLLECTED IN THE STUDY BUT NOT RELEASED AS A LABEL = 0 / NEGATIVE: NON-AFFECT COVARIATE ONLY = 0 / NR or ABSENT: label_model not recorded = 0 | 3 | NR and ABSENT on label_model make the index NOT SCORABLE for that dataset: the procedure returns L-NOT-SCORABLE, the dataset is excluded from the numerator and the denominator of the reported level distribution, and the S19 row carries the reason. |
| ONT-C7 | Does the released label inventory name two or more distinct affect QUALITIES, or is it exhausted by valence polarity? | label_model | TWO OR MORE NAMED AFFECT QUALITIES = 2 / VALENCE POLARITY ONLY = 1 / SINGLE CLASS, BINARY PRESENCE/ABSENCE, OR UNSTRUCTURED FREE TEXT = 1 / NR or ABSENT on label_model = 0 | 2 | Identical to ONT-C5, since the criterion reads the same field: NR and ABSENT return L-NOT-SCORABLE and exclude the dataset from both the numerator and the denominator of the published distribution, rather than defaulting to the L1 floor. |

Total atomic criteria: 52.

### S24.3 Conformance checks during codebook construction

While the codebook was being constructed, each index was drafted and then checked for conformance with its definition in the manuscript. Eight corrections were made to the drafts where a draft read an input the manuscript does not name for that index, or where a rule failed to partition its answer space. They are listed below as a record of how the instrument was built. Identifiers of removed criteria were not reused, so the criterion numbering in Section S24.2 has gaps (RFS-4 and ONT-C6).

A ninth correction was made after the conformance check and is recorded separately because it changed computed scores rather than the wording of a rule. The missing-data policy stated in Section S24.1, under which a criterion that cannot exist for a design is dropped from both numerator and denominator, was not yet applied to AS5 when the scores were first computed. The coding rows carried the descriptive label of the not-applicable state rather than the sentinel value that the scoring engine reads, so the engine treated the declaration as an ordinary zero-point state and retained the criterion in the denominator for every dataset. For 22 of the 27 corpora, the Annotation Specificity value computed in that state therefore differed from the value the published rules give. The self-test that was intended to guard the policy compared the not-reported and not-applicable branches only at the floor of the scale, where both map to 1.0, so it passed while the defect was present; it was therefore extended to check the branch assignment, and it fails if any missing-data declaration is shadowed by a state label. The correction moved Annotation Specificity for 23 of the 27 corpora (DEAP 2.8 to 3.1; SEED 2.2 to 2.5; SEED-IV 2.5 to 2.8; SEED-V 2.5 to 2.8; SEED-VII 2.9 to 3.2; SEED-GER 2.2 to 2.5), that is, the 22 corpora above together with LLaMAC (3.1 to 3.2), whose agreement statistic falls under the out-of-scope rule of AS5, and moved the corpus mean AI-readiness from 2.596 in the uncorrected computation to the reported 2.684, and the median from 2.50 to 2.60. FAIR, the ontology levels and the OFI are unaffected, because AS5 contributes only to Annotation Specificity. It also changed the head of the ordering: the uncorrected computation tied AMIGOS and EmoEEG-MC at 3.23, whereas with the rule applied EmoEEG-MC stands alone at 3.33; the uncorrected values quoted in this paragraph are not part of the reproducibility package, whose data files and scripts give only the corrected scores.

| **Index** | **Change to the draft** | **Target** | **Reason** |
| --- | --- | --- | --- |
| FAIR-F | criterion removed | F5 | The criterion read versioning while being labelled 'indexed metadata'. Section 2.4.1 does not name version citability as an input to Findability. |
| FAIR-I | state removed | I1/DERIVED_ONLY_OPEN | A derivative-only state inverted the published ranking (a .set derivative scored 2 against a .csv time series at 3). The format ranking in Section 2.4.1 is about the container, not about the kind of content, so derivative status was moved to the I3 coverage criterion. |
| FAIR-R | criterion removed | R5 | The criterion charged for the reachability and survival of documentation. Section 2.4.1 explicitly forbids the access axis from contributing to the FAIR score. |
| RFS | criterion removed | RFS-4 | Event and trigger schemas are not named by Section 2.4.3 as an input to RFS. |
| Integration | criterion removed | INT-5 | pid_resolves is not named by Section 2.4.3 as an input to Integration. Resolution verification is absorbed into the evidence requirement of INT-4. |
| OntologyLevel | criterion removed | ONT-C6 | Time-resolved annotation appears nowhere in the L1 to L5 operational definitions of Table 3. |
| SRS | states redefined | SRS-2 | The three states did not partition the space, so a resource with M = 1 (a single dimensional coordinate) could not be scored. They were replaced by sequential evaluation, first match taken, so that the remaining states close the space. |
| TAS | decision rule revised | TAS-4 | The cross-corpus test was reading montage standardisation. Table 2 names montage as an OMS input, so the test was re-specified on label-target intersectability alone. |

### S24.4 Attribute-table specification

The codebook reads 25 fields of the attribute table across 27 datasets. Every cell of these fields is filled (Coverage column). The attribute table is distributed in the reproducibility package (Reproducibility_package.zip) as data/attributes.csv, with the same content in data/attributes.json; each field except access_status is followed by __src and __date companion columns that give the source it was read from and its check date; in 27 cells of access_mode, descriptor_doi and dataset_pid the check date reads “undated (manuscript value)”, as stated under Attribute provenance in Section S24.1. The field access_status records the outcome of the dated route check and draws on the access_route and pid_resolves columns and their check dates. The criterion states coded from these fields are held in data/codings.json.

| **Field** | **Read by** | **Coverage** |
| --- | --- | --- |
| label_model | AS.AS1, TAS.TAS-1, OMS.OMS-1, SRS.SRS-1, SRS.SRS-2, SRS.SRS-3, RFS.RFS-1, RFS.RFS-2, RFS.RFS-3, OntologyLevel.ONT-C5, OntologyLevel.ONT-C7 | 27/27 |
| controlled_vocabulary | OMS.OMS-5, SRS.SRS-4, Integration.INT-1, OntologyLevel.ONT-C1, OntologyLevel.ONT-C2 | 27/27 |
| distribution_format | FAIR-I.I1, FAIR-I.I3, FAIR-I.I4, Integration.INT-3 | 27/27 |
| bids_compliant | FAIR-I.I2, OMS.OMS-4, Integration.INT-2, OntologyLevel.ONT-C3 | 27/27 |
| dataset_pid | FAIR-F.F1, Integration.INT-4 | 27/27 |
| descriptor_doi | FAIR-F.F3, FAIR-F.F4 | 27/27 |
| access_route | FAIR-A.A1, FAIR-A.A3 | 27/27 |
| licence | FAIR-A.A2, FAIR-R.R2 | 27/27 |
| loader_support | FAIR-I.I5, TAS.TAS-6 | 27/27 |
| datasheet | FAIR-R.R3, FAIR-R.R4 | 27/27 |
| label_granularity | AS.AS2, TAS.TAS-2 | 27/27 |
| pid_resolves | FAIR-F.F2 | 27/27 |
| access_status | FAIR-A.A4 | 27/27 |
| access_mode | FAIR-A.A5 | 27/27 |
| licence_class | FAIR-R.R1 | 27/27 |
| rater_design | AS.AS3 | 27/27 |
| trials | AS.AS4 | 27/27 |
| inter_annotator_agreement | AS.AS5 | 27/27 |
| modalities | TAS.TAS-3 | 27/27 |
| supported_tasks | TAS.TAS-4 | 27/27 |
| benchmark_splits | TAS.TAS-5 | 27/27 |
| electrode_metadata | OMS.OMS-2 | 27/27 |
| channels_montage | OMS.OMS-3 | 27/27 |
| stimulus_paradigm | RFS.RFS-5 | 27/27 |
| event_schema | OntologyLevel.ONT-C4 | 27/27 |

### S24.5 Reproducibility package and executable code

The reproducibility package (Reproducibility_package.zip) holds the canonical data in data/ and nine Python scripts in scripts/, together with README.md, which lists every file with its purpose and gives the exact commands; requirements.txt, which gives the Python version and the minimum versions of the third-party packages (only scripts/verify_docs.py needs one of them, openpyxl, to read the workbook; the other eight scripts use the Python standard library alone); and SHA256SUMS.txt, which carries a checksum for every data file, every script and the README. No script writes into data/: every output goes to output/, so a regenerated file can always be compared with its canonical copy. The canonical data are the codebook (data/codebook_v2.json); the attribute table with its sources and check dates (data/attributes.csv, data/attributes.json); the coded criterion states (data/codings.json); the per-dataset sub-scores (data/scores_v2.json, data/scores_v2.csv); the corpus record and its aggregates (data/corpus.json, data/aggregates_final.json); the access-mode codes of the five corpora added in the second search pass (data/access_mode_final.json); the adoption ledger of Section S22 (data/adoption_v2.json); the figure input file (data/figure_data_v2.json); the two-pass identification funnel (data/funnel_v2.json); the risk-of-bias matrix of Section S12 (data/rob_27.json); the three-rater codings and agreement coefficients of Section S11 (data/s11_codings_v2.json, data/s11_reliability_v2.json); the citation-edge counts of the five seed papers of Section S3b, with their Semantic Scholar paper identifiers and query date (data/S3b_citation_counts.csv); the resource-by-resource Stream A reconciliation ledger of Section S4b (data/S4b_streamA_ledger.csv) and the 32 distinct records of the second search pass with their source databases, dispositions and reasons (data/S4b_pass2_records.csv); the eligibility screen of the 72 landscape units of Section S21.2 that match no appraised corpus, with the OpenAlex citation count and query date of each unit (data/landscape_unreached_screen.csv); and the crosswalk of all 102 landscape units of Section S21 to the appraised corpora (data/landscape_crosswalk_102.csv). The table below groups the scripts by stage.

| **Scripts** | **Input** | **Output** | **Verification** |
| --- | --- | --- | --- |
| scripts/codebook_score.py; scripts/propagate_scores.py | codebook and coded criterion states (data/codebook_v2.json, data/codings.json) | sub-scores; the corpus record with its composites, the score table and the aggregate file (all written to output/) | scripts/codebook_score.py --selftest: scale endpoints exact for all ten point-based dimensions; missing-data exploit demonstrated closed; --check on either script: each output equals its canonical copy (data/scores_v2.json, data/corpus.json, data/scores_v2.csv, data/aggregates_final.json) |
| scripts/score_engine.py; scripts/reliability.py | sub-scores in data/corpus.json; codings of the three language-model rater personas in data/s11_codings_v2.json | every reported summary statistic of the scores (printed as JSON); per-item Fleiss kappa, Gwet AC1 and Brennan-Prediger, their mean over the 49 items with a defined kappa (48 criteria and the ontology level), and the bootstrap interval (--boot 10000 --seed 42) | scripts/score_engine.py --selftest reproduces the published FAIR means, AI-readiness mean and median, level distribution, cluster OFI means, sub-score correlations, weight-sensitivity table and corpus-composition analysis; scripts/reliability.py --check regenerates the coefficients and the interval of Section S11 and compares them with data/s11_reliability_v2.json |
| scripts/gen_tables.py; scripts/gen_s24.py; scripts/build_figure_data.py; scripts/gen_figure4.py | canonical data in data/ | the numeric content of Supplementary Tables S15, S17, S18 and S19, of manuscript Table 3 and of the weight-sensitivity table of Section S14.1, a condensed extract of the attribute table for Supplementary Table S16, the criteria tables of this section and the ledger tables of Section S4b (output/tables/); the per-dataset figure input file; the two-pass identification flow diagram with the counts of manuscript Figure 3 (output/Figure4_PRISMA_flow.html) | the generated tables are written from the same record as the reported statistics and can be compared with the tables in the documents; scripts/build_figure_data.py --check compares the figure input file with data/figure_data_v2.json |
| scripts/verify_docs.py | the manuscript, the supplement and the transparency workbook (--main, --supp, --workbook), read against data/ | a report of each check, with a non-zero exit status if any check fails | recomputes the canonical statistics from data/ and confirms they appear in the documents; confirms the adoption coefficients in sheet S22_adoption, the rank order of sheets S15_scorecard and S18_AIreadiness and the 27-corpus, 42-identifier scope stated in sheet S20_DOI_verification; confirms that every file cited in the documents is present in the package and that the checksums in SHA256SUMS.txt match; and confirms that sheet S11_codings equals data/s11_codings_v2.json row by row, that data/S4b_pass2_records.csv reconciles with the counts of Section S4b and of the legend of manuscript Figure 3, the two-pass flow, and that every numeric cell of Supplementary Tables S15, S17, S18, S19 and S22 equals the data |

The nine scripts form a single chain from the coded criterion states to every score and score-derived statistic reported in the manuscript, the supplement, the transparency workbook and the figure captions. scripts/codebook_score.py converts the coded states in data/codings.json to sub-scores using only the point ladders and missing-data policies published in this section (data/codebook_v2.json). scripts/propagate_scores.py carries those sub-scores into the corpus record, recomputes the composites under the rounding rules stated above and rebuilds the score table and the aggregate file, writing all three to output/. scripts/score_engine.py derives every reported summary statistic and correlation of the scores, every weight-sensitivity row and the corpus-composition sensitivity from the sub-scores in data/corpus.json alone. scripts/gen_tables.py regenerates the numeric content of Supplementary Tables S15, S17, S18 and S19, of manuscript Table 3 and of the weight-sensitivity table of Section S14.1 from the same record, and writes a condensed extract of data/attributes.json for Supplementary Table S16, whose descriptive cells summarise that file rather than reproduce the extract. scripts/gen_s24.py writes the criteria tables of this section from data/codebook_v2.json, with long cells cut at fixed lengths, so that every criterion, attribute field, state, point value and maximum shown here can be checked against the file the scorer reads; it also writes the ledger tables of Section S4b. scripts/build_figure_data.py emits the per-dataset figure input file, scripts/gen_figure4.py draws the two-pass identification flow reported in manuscript Figure 3 from the funnel record (data/funnel_v2.json), scripts/reliability.py regenerates the per-item coefficients and the bootstrap interval of Section S11 from data/s11_codings_v2.json and checks them against data/s11_reliability_v2.json, and scripts/verify_docs.py checks the manuscript, the supplement and the workbook against the canonical data and confirms that every file they cite is present in the package. README.md gives the exact commands in the order in which the record is built, from scripts/codebook_score.py and scripts/propagate_scores.py to scripts/reliability.py, and the expected key outputs; scripts/verify_docs.py is run last, against the documents. Two regression suites guard the chain. scripts/codebook_score.py --selftest confirms that each dimension lands exactly on its scale endpoints when every criterion takes its lowest and its highest state, and checks that the published missing-data policy actually fires: a criterion declared not applicable must be recorded with the sentinel value that removes it from the denominator, because a state label that merely begins with the letters NA is scored as an ordinary zero and leaves the denominator intact. That check was added during construction of the codebook, after this defect was found in AS5, where the not-applicable branch documented here had never been applied to any dataset; the correction and its effect on the reported scores are recorded in Section S24.3. scripts/score_engine.py --selftest reproduces the corpus statistics, the cluster means, the weight-sensitivity rows and the SEED-collapse sensitivity from the canonical record and compares each against the value reported in the manuscript and in this supplement, so a divergence between code and text fails the suite rather than reaching the reader.

## 1.1 S25 Supplementary Figures

**Supplementary Figure S1.** FAIR principle scores of the 27 appraised datasets. Each row is one dataset, numbered by its ID in manuscript Table 1, with its access class, distribution format and data licence. Access class is shown as a shape and a word: circle, fully open; square, gated (registration- or licence-gated); diamond, conditional. The four FAIR principles, Findability (F), Accessibility (A), Interoperability (I) and Reusability (R), are each scored 0.0 to 4.0, and the FAIR composite is their mean, printed to three decimals as in Supplementary Table S17. Cell shading follows one single-hue ramp from light (1.0) to dark (4.0); all 108 principle scores lie in that range, and the figure uses no other colour. Rows are ordered by composite, highest first, with ties in ascending ID, and are grouped under neutral composite-range labels: 3.50 to 4.00 (n = 4), 3.00 to 3.49 (n = 7) and below 3.00 (n = 16). Corpus means: F = 3.311, A = 3.511, I = 2.044, R = 2.563 and composite 2.857. In the format column, BIDS-EEG marks the two deposits that validate against BIDS-EEG (EmoEEG-MC and Daly) and BIDS-partial the two that follow it in part (IESD and DENS). DENS carries conflicting licence statements in one deposit (CC0 in its dataset description, the Open Database License in its LICENSE file) and is scored on the more restrictive, shown as CC-BY-SA. EAV declares CC BY 4.0 but releases its files under a data-use agreement, hence its conditional access class. Access class contributes no points to the FAIR score (manuscript Section 2.4.1).

**Supplementary Figure S2.** Ontology Formalisation Index (OFI) of the 27 appraised datasets with its three sub-indices, each scored 1.0 to 4.0: semantic-relation structure (SRS), representation formalism (RFS) and external-standard linkage (Integration). The OFI is their mean, printed to three decimals as in Supplementary Table S19. Rows are grouped by the six task clusters of manuscript Table 1; a small chip beside each cluster name, in that cluster's colour in Table 1, is the only categorical colour in the figure. Clusters are ordered by mean OFI, and rows within a cluster by OFI, highest first, with ties in ascending ID; numbers are the Table 1 IDs. The badge gives each dataset's ontology level: L1 flat label (n = 4), L2 basic-emotion taxonomy (n = 7) and L3 relational or dimensional affect model (for example valence-arousal-dominance; n = 16); no dataset is at L4 or L5. Cell shading follows one single-hue ramp from light (1.0) to dark (4.0); the values shown run from 1.0 to 3.5, so no sub-index reaches 4.0. Cluster mean OFI: continuous-naturalistic 3.233 (n = 2), wearable-field 3.050 (n = 2), multimodal 2.889 (n = 6), dimensional-VAD 2.556 (n = 3), dialogue-social 2.478 (n = 3) and discrete-multi-emotion 1.764 (n = 11). The clusters are small and their ranges overlap (for example multimodal 2.533 to 3.433 and discrete-multi-emotion 1.100 to 2.867), so these means describe the groups and do not rank them. Corpus means: SRS 2.667, RFS 2.474, Integration 2.015 and OFI 2.385. Semantic-relation structure reaches its observed maximum of 3.5 in nine datasets, whereas external-standard linkage reaches 3.5 only in EmoEEG-MC and the Daly deposit (ds002721).
